# The Genetic Legacy of African Americans from Catoctin Furnace

**DOI:** 10.1126/science.ade4995

**Published:** 2023-08-04

**Authors:** Éadaoin Harney, Steven Micheletti, Karin S. Bruwelheide, William A. Freyman, Katarzyna Bryc, Ali Akbari, Ethan Jewett, Elizabeth Comer, Henry Louis Gates, Linda Heywood, John Thornton, Roslyn Curry, Samantha Ancona Esselmann, Kathryn G. Barca, Jakob Sedig, Kendra Sirak, Iñigo Olalde, Nicole Adamski, Rebecca Bernardos, Nasreen Broomandkhoshbacht, Matthew Ferry, Lijun Qiu, Kristin Stewardson, J. Noah Workman, Fatma Zalzala, Shop Mallick, Adam Micco, Matthew Mah, Zhao Zhang, 23andMe Research Team, Nadin Rohland, Joanna L. Mountain, Douglas W. Owsley, David Reich

**Affiliations:** 1 23andMe, Inc.; Sunnyvale, CA 94043, USA.; 2 Department of Human Evolutionary Biology, Harvard University; Cambridge, MA, 02138, USA.; 3 Department of Anthropology, National Museum of Natural History, Smithsonian Institution; Washington DC 20560, USA.; 4 Department of Genetics, Harvard Medical School; Boston, MA, 02115, USA.; 5 Catoctin Furnace Historical Society; Thurmont, MD, 21788, USA.; 6 Hutchins Center for African and African American Research, Harvard University; Cambridge, MA 02138, USA.; 7 Department of History/African American Studies, Boston University; Brookline, MA 02446, USA.; 8 BIOMICs Research Group, Department of Zoology and Animal Cell Biology, University of the Basque Country UPV/EHU, Vitoria-Gasteiz, Spain; 9 Ikerbasque—Basque Foundation of Science, Bilbao, Spain.; 10 Howard Hughes Medical Institute, Harvard Medical School; Boston, MA, 02115, USA.; 11 Broad Institute of MIT and Harvard; Cambridge, MA, 02142, USA.

## Abstract

Few African Americans have been able to trace family lineages back to ancestors who died before the 1870 United States Census, the first in which all Black people were listed by name. We analyze 27 individuals from Maryland’s Catoctin Furnace African American Cemetery (1774–1850), identifying 41,799 genetic relatives among consenting research participants in 23andMe, Inc.’s genetic database. One of the highest concentrations of close relatives is in Maryland, suggesting that descendants of the Catoctin individuals remain in the area. We find that many of the Catoctin individuals derived African ancestry from the Wolof and Kongo groups and European ancestry from Great Britain and Ireland. This study demonstrates the power of joint analysis of historical DNA and large datasets generated through direct-to-consumer ancestry testing.

## One Page Summary

**Introduction:**

Genetic analysis of historical individuals has the potential to help restore knowledge of people whose stories were omitted from written records. During the late 18^th^ and early 19^th^ centuries, Catoctin Furnace in Maryland relied on a workforce of enslaved individuals to operate the iron furnace and carry out domestic and agricultural tasks. Despite the role that Catoctin Furnace played in early United States history (including supplying munitions to combatants during the Revolutionary War), relatively little is known about the African Americans who labored there or their descendants compared to the furnace’s later predominantly white workforce.

**Rationale:**

We produced whole-genome data for 27 individuals buried in the Catoctin Furnace African American cemetery and compared them to approximately 9.3 million consenting research participants genotyped by 23andMe, Inc. to address the following questions: (i) How were the Catoctin individuals related to each other? (ii) What were the sources of their African and European ancestry? (iii) Where in the United States do their genetic relatives live today, including their direct descendants? (iv) What can their genomes reveal about their health?

**Results:**

**Fig. 0.**
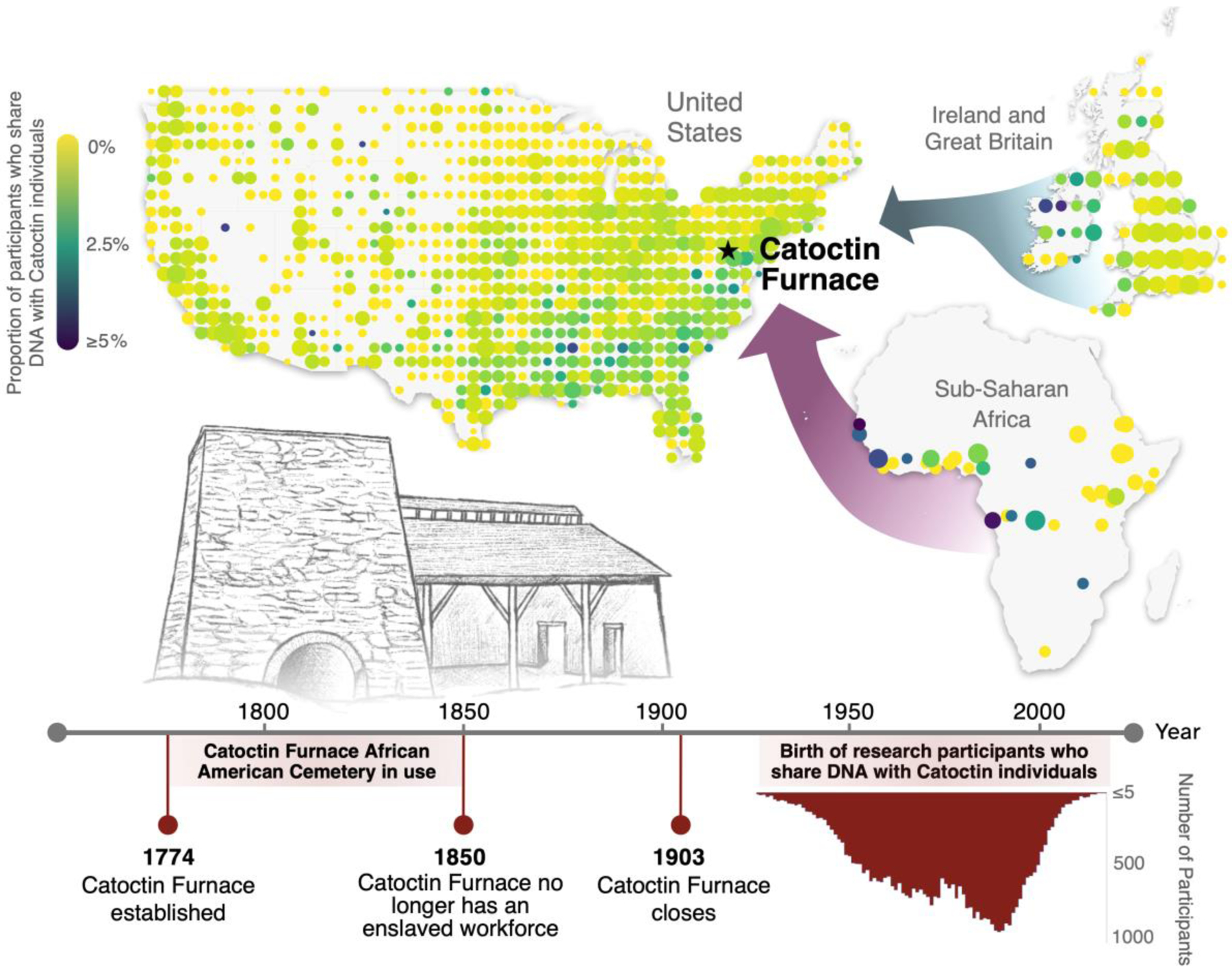
Genetic connections to individuals from Catoctin Furnace African American Cemetery in Maryland. A timeline highlighting major events in the history of Catoctin Furnace and the birth years of research participants in the 23andMe cohort, presented alongside maps showing the proportion of 23andMe research participants associated with geographic coordinates in the United States, Sub-Saharan Africa, and Europe who share genetic connections to the Catoctin individuals.

We identified five genetic families, consisting of biological mothers, children, and siblings, among the Catoctin individuals. In most cases, biological family members were buried in close proximity. All but one of the Catoctin individuals had primarily African ancestry, with variable amounts of European ancestry. To learn more about their ancestry we developed a new approach to detect identical-by-descent segments of the genome shared between the Catoctin individuals and 23andMe research participants. Identical-by-descent segments of DNA are shared by two or more people because they have been inherited from a recent common ancestor. We identified 41,799 close and distant relatives of the Catoctin individuals among 23andMe research participants (Fig 0). Within Africa, we found the highest rates of genetic sharing between Catoctin individuals and research participants who self-identified as belonging to the Wolof or Kongo ethnolinguistic groups. Within Europe, we observed the highest rates of genetic sharing with research participants that have ties to Great Britain and Ireland.

Within the United States, we observed elevated rates of sharing with participants from the South, largely reflecting distant connections to 23andMe research participants with Sub-Saharan African ancestry (possibly tracing back to shared common ancestors in Africa). When we considered genetic relatives who share the most identical DNA with the Catoctin individuals, we observed the highest rates of sharing in Maryland, suggesting that at least some descendants stayed in the region following the furnace’s transition away from enslaved and paid African American labor. Finally, we found that some of the Catoctin individuals carried risk factors for sickle cell anemia and G6PD deficiency, genetic diseases that are common in African Americans today.

**Conclusion:**

These results demonstrate the power of joint analysis of DNA from historical individuals and the extremely large datasets generated through direct-to-consumer ancestry testing, and serve as a model for obtaining direct insights into the genome-wide genetic ancestry of enslaved people in the historical United States.

## One Sentence Summary

Identifying genetic connections between historical African Americans from Catoctin Furnace and present-day people.

The vast majority of the ~45 million self-identified Black and/or African American individuals living in the United States (US) descend from approximately 456,600 enslaved Africans who were forcibly transported to the US from Africa during the transatlantic slave trade between 1501 and 1867 (1, 2). However, African Americans often have little information about these ancestors or their African origins due to a history of inhumane treatment of the enslaved and their descendants, which included marginalization and the obfuscation of family histories (3). In this study, we demonstrate that when combined with genome-wide data from a sufficiently large and diverse genetic database, DNA from historical individuals provides a means for restoring knowledge of familial connections between contemporary peoples and their historical relatives. Specifically, we report on the DNA of enslaved and free African Americans from Catoctin Furnace, Maryland, who lived, worked, died, and were buried there in the late 18^th^ and early 19^th^ centuries.

**Fig. 1.**
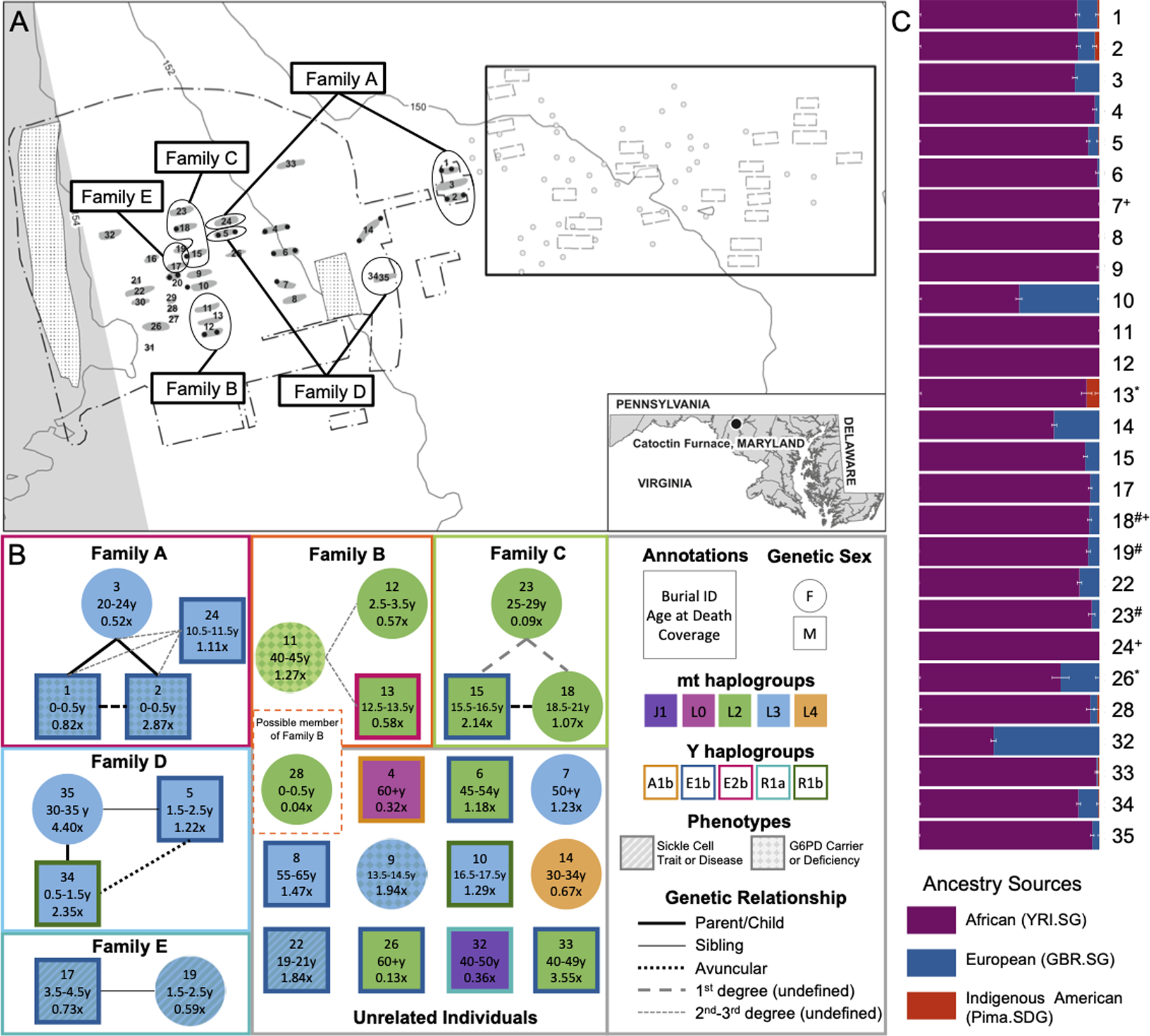
Burial context, genetic kinship, and ancestry of Catoctin individuals. (**A**) Map showing the location of Catoctin Furnace and burials within the cemetery. Burial locations of the five genetic families are circled. The rectangle in the upper right shows a portion of the cemetery with unexcavated burials identified through ground penetrating radar (map adapted from (12), prepared by Robert Wanner). (**B**) Individuals, labeled according to burial ID, are grouped into families based on genetic relationships. Genetic sex, mt and Y haplogroups are indicated by marker shape, fill, and outline color, respectively. The type of genetic relationship is indicated by connector linestyle. Marker fill pattern indicates individuals with one or more copies of an allele associated with sickle cell anemia or glucose-6-phosphate-dehydrogenase (G6PD) deficiency. (**C**) Ancestry proportions assigned to each individual from representative African (YRI), European (GBR) and Indigenous American (Pima) populations drawn from the public dataset according to the qpAdm software. Error bars indicate one standard error. Asterisks (*) indicate cases where damage restricted data were analyzed. Hash symbols (#) and plus signs (+) indicate models with p-values <0.01 or ancestry proportion estimates that fall more than three standard errors outside the range of 0–1, respectively.

As early as December 1768, a tract of land was acquired in order to build an iron works at the foot of Catoctin Mountain near present-day Thurmont, Maryland (4). The furnace was in blast by 1776, producing pig iron, tools, household items, and munitions used during the Revolutionary War. At least 271 enslaved and an unknown number of free African Americans worked at Catoctin within and outside the furnace, as ore miners, colliers, forgemen, fillers, teamsters, and woodcutters, as well as in domestic and agrarian roles in the furnace owners’ households and plantations (5). In the second quarter of the 19^th^ century, the furnace’s labor force switched primarily to wage labor and a primarily white workforce (6). Gradually, the contribution of African Americans in this early industrial complex was largely forgotten. The Catoctin Furnace African American cemetery, near an old ore pit, was excavated in 1979–1980 in advance of highway construction (Fig. 1A) (7–10). The Maryland State Highway Administration transferred stewardship of the recovered remains of deceased humans to the Smithsonian Institution, where curator Dr. J. Lawrence Angel conducted preliminary forensic anthropological investigations (11).

The Catoctin Furnace Historical Society, Inc. (CFHS) was initially founded to save the Catoctin Furnace village and its archaeological and architectural heritage from this highway construction (4). In recent years, its mission expanded to include restorative justice, highlighting the critical role that enslaved and free African Americans played in the furnace’s history and in the growth of industrial wealth and power in the young US. In 2015, a grant from the Maryland Heritage Areas Authority supported further scientific analysis of individuals buried in the cemetery. The first phase of the project involved historical documentary research and osteological re-analysis that refined previous assessments of demography and bone and dental pathology, with testing for stable carbon and nitrogen isotopes, and trace elements to shed light on the life histories of the individuals (12). The project’s second phase used DNA to explore their biogeographic ancestries and relationships to one another, details of which were summarized in a technical note (13) and are expanded upon here. These data address a critical component of CFHS’s mission, pursued jointly with the African American Resources Cultural and Heritage Society (AARCH) of Frederick County, to return knowledge to the African American community and identify descendants of Catoctin’s enslaved and free workers.

Advances in ancient DNA (aDNA) technology have made it possible to use genetic data as a tool for restoring knowledge of enslaved and historically marginalized peoples whose stories were often omitted from or disregarded in written records. Studies of the New York African Burial Ground (14, 15), the Anson Street ancestors (16, 17) and others (18, 19) used a combination of anthropological and biomolecular tools to provide insight into the identity and life history of enslaved individuals through the study of their remains. However, their ability to localize the African origins of these individuals was limited by the exclusive use of mitochondrial (mt) DNA (20) and/or reliance on comparisons with data from publicly available reference datasets (21, 22). This study shows how deeper insights into the precise ancestral origins and genetic legacy of enslaved and free African Americans, such as those buried at Catoctin Furnace, can be obtained from genome-wide aDNA data when compared to a reference database containing genetic data from millions of living people, like the one maintained by 23andMe, Inc.

We recovered genome-wide aDNA from all 27 of the Catoctin individuals who were selected for sampling, targeting ~1.2 million single nucleotide polymorphisms (SNPs) using a capture-based approach (23–26), which we combined with imputation to further increase the amount of available genetic information (Table S1). By comparing the DNA of the Catoctin individuals with genotype data from 9,255,493 participants in the 23andMe cohort, all of whom consented to participate in research, we were able to learn about their biogeographic ancestries, genetic relationships with one another, and to provide insight into their genetic legacy by identifying identical-by-descent (IBD) connections with living relatives.

## Community Engagement and Ethical Considerations

This research analyzed data from deceased individuals who were unable to directly consent to participate in this study, as well as from millions of research participants (including those genotyped by 23andMe) who actively consented to participate in research. The ties between present-day African Americans, their ancestors within the US, and their ancestors in Africa were forcibly severed by the transatlantic slave trade, the centuries-long institution of slavery, and generational systemic racist practices that have endured after the abolition of race-based slavery (27), as illustrated by Frederick Douglas’ famous words: “Genealogical trees do not flourish among slaves” (28). Our objective is to contribute to the restoration of memories of a past community whose legacy was intentionally obscured and to create an avenue for living people to learn about their ancestors. We followed guidelines for the ethical analysis of the DNA of historical and living people (29, 30), including consultation with stakeholder groups, as emphasized in recent discussions on the future of studies involving the remains of African Americans (31–35). Based on interactions with stakeholders, we believe there is interest among African Americans and the public to harness aDNA to learn about historical connections to people who lived in the past, and to leverage this technology to develop accurate methods to identify genetic relationships, many of which were previously unknown. Equally important is the need to communicate the results of these analyses with descendants and others in a sensitive and accurate manner.

In the case of Catoctin Furnace, the goals of CFHS, developed in partnership with AARCH, include identifying descendants and widening the community of stakeholders. To date, researchers at CFHS have traced the lineages of two African Americans (one enslaved and one free) who labored at Catoctin Furnace to their living descendants by utilizing historical documents and genealogical data. Members of CFHS, AARCH, and the recently discovered descendants all expressed support for the use of genetic approaches that could identify a larger descendant community. Based on conversations with these stakeholders about their research interests, we sought not only to identify living genetic relatives of the Catoctin individuals, but also to conduct analyses to shed light on their life stories, such as identifying family relationships shared between the Catoctin individuals, exploring their African and European origins, and identifying biologically significant variants. In collaboration with CFHS and AARCH we also held a series of public events to directly return research results at various phases of the project (36). One of the ways in which stakeholders chose to honor the legacy of enslaved individuals at Catoctin during these events was through the act of reading the names of individuals that could be abstracted from a variety of sources, including land records, probate inventories, church records, diaries, and freedom-seeker ads (although not associated with specific burials) (5). We include these names in the supplementary text (36). Establishing family connections to living people through genetics contributes to remembering and honoring those buried at Catoctin.

In this study, we show that the joint analysis of DNA extracted from the remains of deceased humans and millions of living people in a re-contactable research cohort (i.e., a cohort in which participants can be asked follow-up questions or receive results), like the one maintained by 23andMe, makes it possible to recover previously unknown connections between present-day people and historical individuals from sites like Catoctin Furnace. Not all members of the Catoctin stakeholder community have a known genetic connection to Catoctin. While this study is responsive to community requests to use genetic approaches to identify descendants, future studies applying these methods should be cautious about the danger of contributing to the biologization of notions of identity, as genetic connections represent one of many ways in which people may feel connected to historical individuals. A full ethics statement is provided in (36). Additional ethical considerations involving the co-analysis of aDNA and data from private genetic databases are discussed in (37).

## Multiple families buried at Catoctin Furnace

Among the 27 Catoctin individuals, we identified five distinct genetic families (labeled A–E) that are primarily composed of mothers, children, and siblings; in this study, the term “genetic family” refers to a group of people who are closely related biologically. Similarly, specific relationship terms (e.g., mother, son, or daughter) are used in a biological sense and based on genetic sex as inferred by the presence or absence of sex chromosomes; thus, they may not reflect the actual kin-based relationships, biological presentation, or gender identities recognized by the Catoctin individuals. Fifteen Catoctin individuals could be assigned to one of the five genetic families, while the remaining 12 individuals appear genetically unrelated, except for the individual from Burial 28, whose coverage was insufficient to confidently assign to Family B. Some unrelated individuals share mt or Y-chromosome haplogroups, which may indicate more distant relationships that fall outside the limits of our resolution. We used information about genetic sex and mt and Y haplogroups to further resolve these family groupings (Fig. 1B). Close genetic relatives tended to be buried near one another (Fig. 1A), while individuals who were buried separately from their genetic families were typically more distantly related. For instance, Family A consists of a mother (Burial 3) and two sons (Burials 1 and 2), interred side-by-side, in addition to a 2^nd^–3^rd^-degree relative (Burial 24) who was buried separately and whose exact relationship to the other individuals is unresolved. While genetic relatedness had a role in burial patterning, other factors, such as temporal context and cultural and religious practices likely contributed as well.

## Sex-biased reproduction

The European ancestry of enslaved African Americans originated largely through a process whereby white men reproduced with Black women through rape. This gender-based sexual violence contributed to the brutal systematic enslavement of African Americans and frequently produced children born into slavery (22, 38). This pattern of behavior, a form of sex-biased admixture, is reflected in the distribution of the mt and Y haplogroups observed among the Catoctin individuals. Three of the 16 Catoctin males have Y haplogroups that are broadly associated with West Eurasian ancestry (Fig. S1). These include subclades of the R1a and R1b haplogroups which are common throughout Europe, indicating that their paternal lineages likely trace back to a fully European ancestor. In contrast, only one individual (Burial 32) has a European-associated mt haplogroup (J1b1a1a) (Fig. S2). This individual is an outlier with respect to ancestry as they also have a European-associated Y haplogroup (R1a1a1b1a3b) and over 50% European ancestry. Among other possible causes, their spatially separated grave, located in the northwestern edge of the burial ground, may reflect their distinct ancestral origins or lack of relatedness within the community represented in the cemetery.

## Variable proportions of African, European, and Indigenous American-related ancestry in Catoctin individuals

All individuals (except the individual from Burial 32) were assigned a majority of African ancestry by qpAdm, using a model designed to estimate each individual’s African, European, and Indigenous American ancestry proportions (Fig. 1C, Table S2–3). Over one quarter of individuals (n=7) could be modeled as having no detectable European admixture (i.e., the amount of ancestry assigned to the European source population is within a single standard error of zero). This is in sharp contrast to nearly all present-day self-identifying African Americans who typically have at least some European-derived ancestry (e.g., there is an average of 24% European ancestry among 23andMe research participants who self-identify as African American (39)). Several individuals could be modeled as having Indigenous American ancestry, but in all cases, estimates were within three standard errors of zero. It is therefore uncertain based on these analyses alone whether these estimates represent true Indigenous American ancestry.

We next imputed genotypes across the whole genome for each Catoctin individual using the software GLIMPSE (40) with an approach optimized for low-coverage, capture-based aDNA data. To ensure that the imputation process would not bias results, we tested the performance of the Templated Positional Burrows–Wheeler Transform (41) (TPBWT) IBD detection tool and 23andMe’s Ancestry Composition (42) tool on a dataset composed of 48 high-coverage ancient individuals who were downsampled to varying degrees (36). Further, we compared the results of ADMIXTURE (Fig. S3), PCA (Fig. S4), and qpAdm (Fig. S5) analyses using the imputed and non-imputed datasets to ensure that the imputation process did not substantially bias ancestry estimates. We observed a bias towards estimating excess European-related ancestry in the lowest coverage individuals when using the imputed dataset relative to the non-imputed dataset; however, this did not appear to substantially impact individuals with >0.5x coverage, which is the group we focused on for subsequent analyses.

**Fig. 2.**
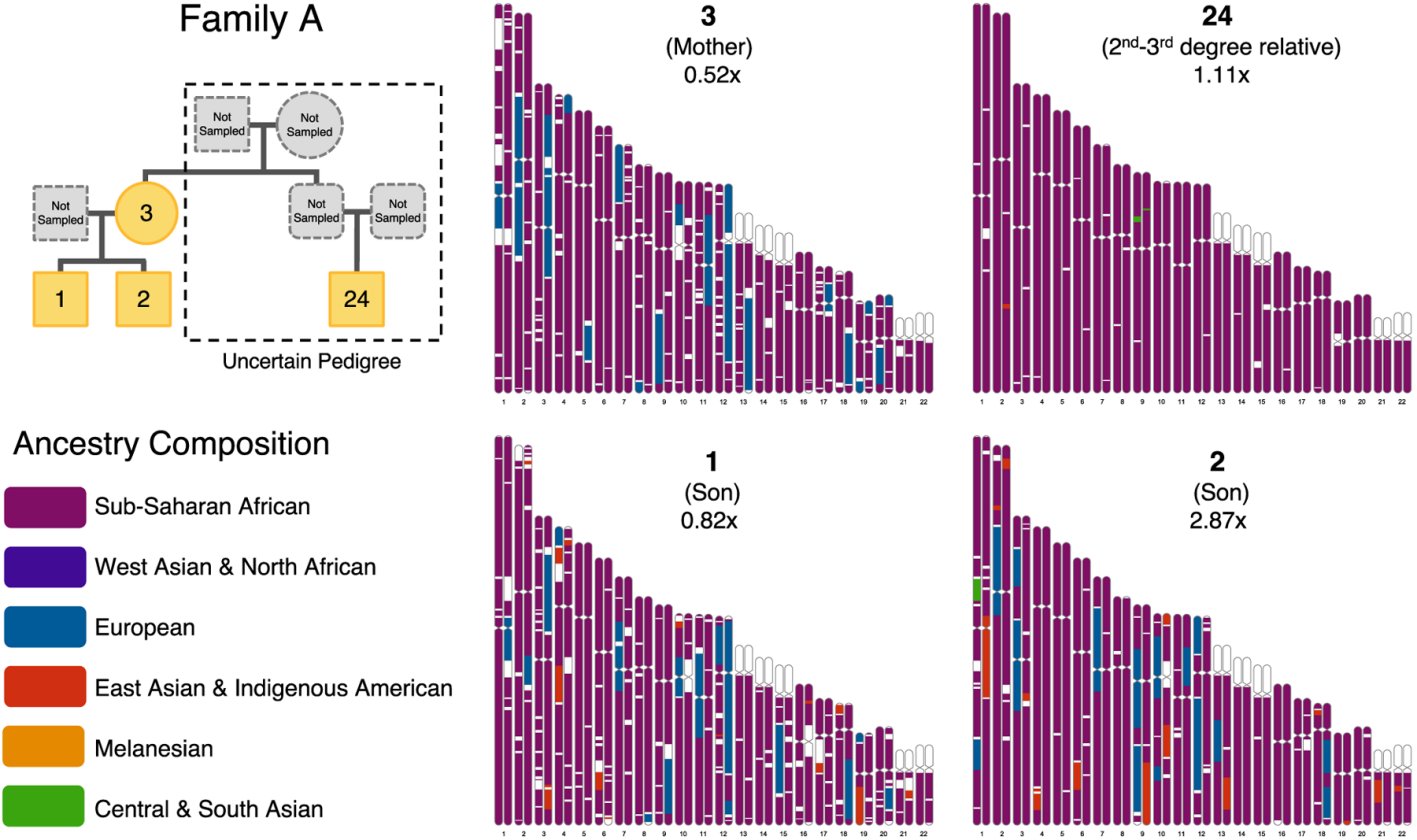
Ancestry composition chromosome paintings for members of Family **A.** Chromosome paintings demonstrating the biogeographic ancestry assigned to portions of the autosomal chromosomes for four related Catoctin individuals assigned to Family A—a mother, two sons, and their 2^nd^–3^rd^-degree relative. A likely pedigree for Family A (top left) describes their relationship to one another, although we note that the true relationship of the individuals from Burials 1, 2, and 3 to the individual from Burial 24 is uncertain. Across the genome, ancestry is assigned to one of six ancestry components defined using 23andMe reference populations: Sub-Saharan African (purple), West Asian & North African (dark blue), European (dark teal), East Asian & Indigenous American (orange), Melanesian (yellow), and Central & South Asian (green). Unassigned regions are shown in white.

Using the imputed dataset, we applied 23andMe’s Ancestry Composition tool (42) to infer local ancestry along the chromosomes of the Catoctin individuals. Each region of their genomes was assigned to one of six previously determined broad ancestry categories: Sub-Saharan African, European, East Asian & Indigenous American, Western Asian & North African, Melanesian, and Central & South Indian (Fig. S6, Table S4). These assignments are correlated with qpAdm estimates (Fig. S7) and provide support for the identification of Indigenous American ancestry in several Catoctin individuals. For instance, we inferred low levels of Indigenous American ancestry in two brothers (Burials 1 and 2) from Family A, but not in their mother (Burial 3) (Fig. 2), suggesting that their un-sampled father had some Indigenous American ancestry.

## Identity-by-Descent (IBD)

To learn more about the biogeographic ancestry and genetic legacy of the Catoctin individuals, we searched for identical-by-descent (IBD) segments of the genome–that is, long segments of DNA that are identical in two or more people because they have been inherited from a recent common ancestor. We searched for IBD shared between each of the Catoctin individuals and ~9.3 million 23andMe research participants. We identified 55,342 IBD segments shared between the historical Catoctin individuals and 41,799 research participants, ranging up to 60 cM in length (Table 1, Figs. S8–9, Tables S5–6). We calculated the total IBD shared between each pair of individuals to estimate their most likely genetic relationship; however, we caution that we are likely underestimating the true amount of DNA shared between these individuals, particularly among close relatives (36). In Box 1, we discuss how the relationships between Catoctin individuals and research participants with whom they share DNA can be interpreted (Table S7), noting that not all present-day individuals who share DNA with Catoctin individuals are direct descendants. In fact, most connections are likely between collateral relatives—relatives who are neither direct ancestors nor descendants of one another, but instead both descend from a common ancestor who lived generations before the Catoctin individuals. Further, many of the most distant relatives that we identified may not share a common ancestor who lived in the Americas. Instead, their connection may trace back to an individual who lived in Africa or Europe prior to their descendants’ arrival in the Americas, either willingly or as part of the transatlantic slave trade.

### Genetic connections to present-day Africans

**Fig. 3.**
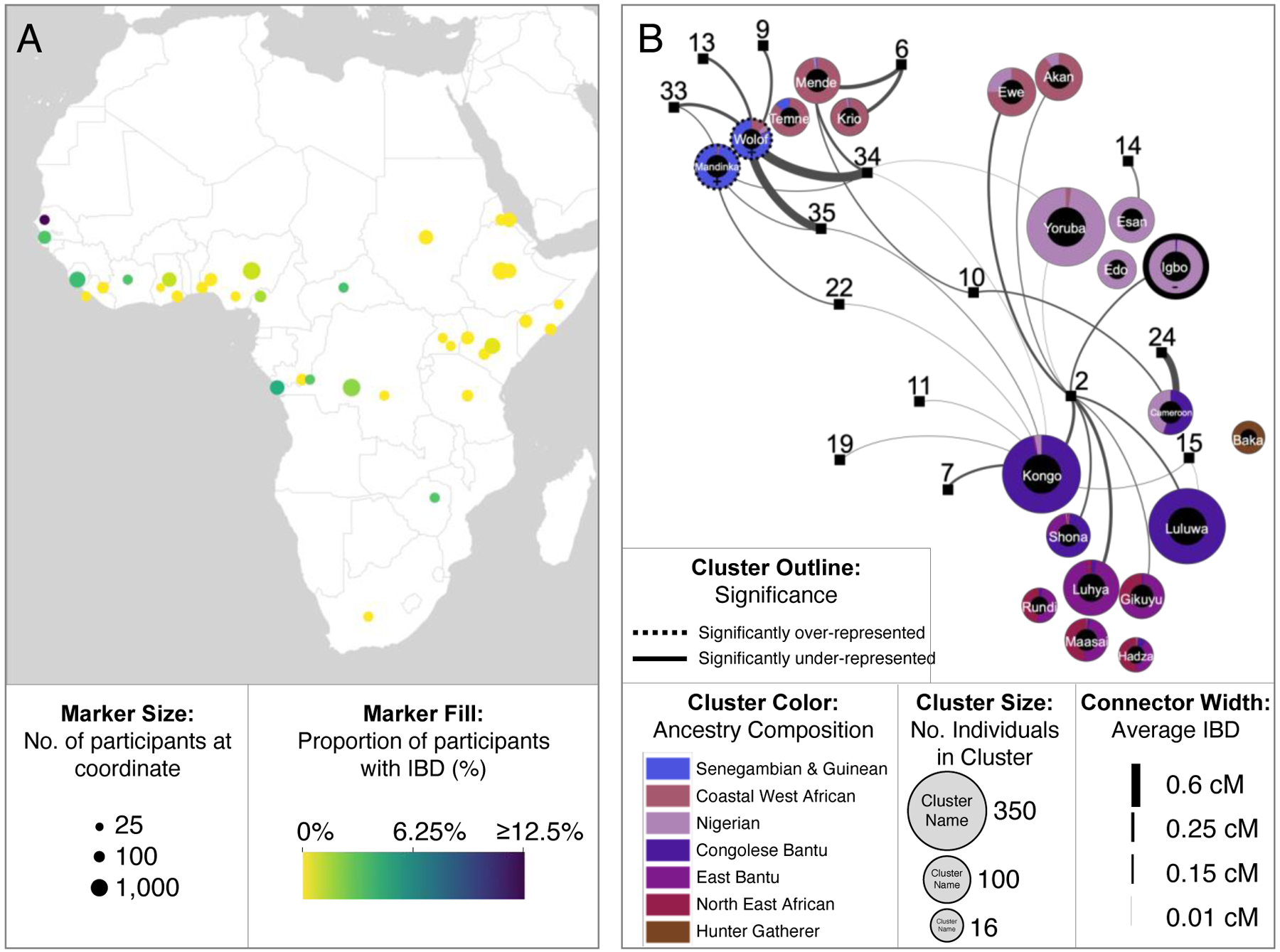
Genetic connections to the Catoctin individuals among members of the African cohort. (**A**) The proportion of 23andMe participants in the African cohort who share IBD with Catoctin. Geographic coordinates are rounded to the nearest integer, and only coordinates that have at least 25 associated participants after 80% downsampling are shown. Marker size indicates the number of participants associated with each coordinate and color indicates the proportion of participants who share IBD with the Catoctin individuals. (**B**) IBD network demonstrating Catoctin individuals’ connections to members of the African cohort who share less than 700 cM of IBD with one another (N = 2,807). IBD clusters (represented by circles) are filled according to members’ average local African ancestry and arranged by average pairwise IBD sharing between clusters using a Force Atlas graph layout. Catoctin individuals, displayed as squares, are projected based on their average IBD shared with each cluster (shown as lines).

We examined IBD segments shared between Catoctin individuals and members of the African cohort (i.e., 23andMe research participants with ≥95% Sub-Saharan African ancestry who indicated that either they or all four of their grandparents were born in Africa) to identify the geographical regions in Africa with which these present-day people are associated. We observed the highest rates of IBD sharing between the Catoctin individuals and participants linked to Senegal, Gambia, Angola, and Democratic Republic of the Congo (DRC) (Fig. 3A, Fig. S10, and Table S8), confirming via randomization testing that we would be unlikely to detect the same number of IBD connections (n=10) with an identically sized sample (n=166) of randomly selected individuals from the Atlantic African cohort (i.e., research participants in the African cohort with ties to specific Atlantic African countries, defined in (36)) (p-value<0.001) (Table S9).

Many African ethnolinguistic groups occupy wide geographic ranges that cross present-day national borders. We therefore also determined the amount of IBD each Catoctin individual shared with genetic clusters in Atlantic Africa that correspond to the self-identified ethnolinguistic groups of research participants (Fig. 3B, Table S10). Of the 15 Catoctin individuals with detectable IBD connections to Africa, seven share a connection with only a single cluster. Six of these individuals have high proportions of Sub-Saharan African ancestry (>90%). In contrast, it is less common for present-day research participants with four grandparents born in the US and ≥50% Sub-Saharan African ancestry to have a connection to only a single cluster (36). Among the Catoctin individuals with connections to Atlantic Africans, the most commonly observed connections are to genetic groups associated with the Wolof, Mandinka, and Kongo (whose present-day geographic distribution is described in Table S11). Overall, the Catoctin individuals share relatively more connections with Senegambian groups, like Wolof and Mandinka, than do research participants with four grandparents born in the US and ≥50% Sub-Saharan African ancestry (Fig. 3, Supplementary Text S5).

### Genetic connections to present-day Europeans

**Fig. 4.**
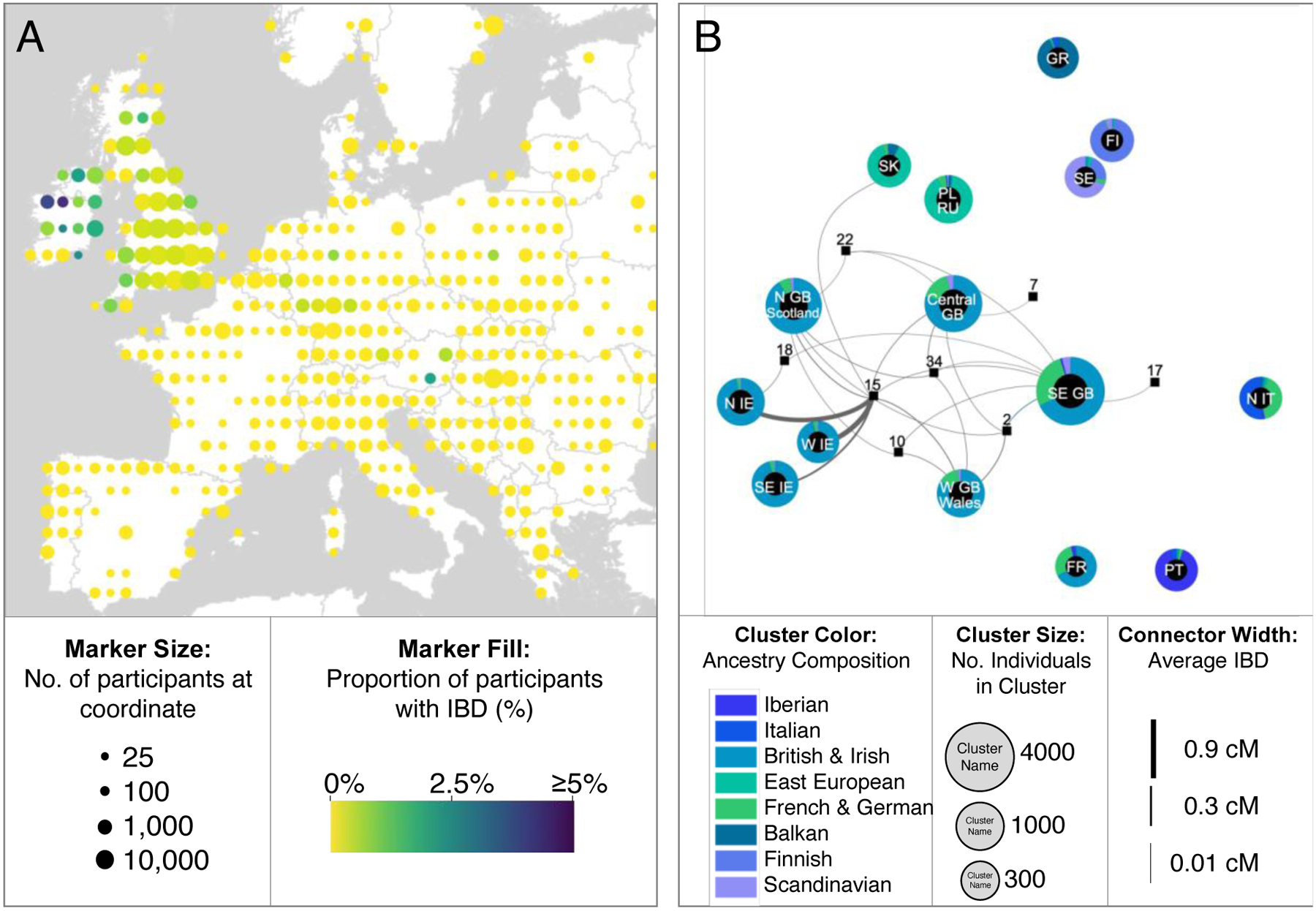
Genetic connections to the Catoctin individuals among members of the European cohort. (**A**) Proportion of 23andMe participants in the European cohort who share IBD with Catoctin. Geographic coordinates are rounded to the nearest integer, and only coordinates that have at least 25 associated participants after 80% downsampling are shown. Marker size corresponds to the number of participants associated with each location, while the color indicates the proportion of participants who share IBD with the Catoctin individuals. (**B**) IBD network indicating Catoctin individuals’ connections to the 23andMe participants in the European cohort who share less than 700 cM with one another. Clusters are labeled by the geographic region with which the majority of cluster members are associated using ISO2 country abbreviations and when appropriate, prefixes to indicate the cardinal directions. Clusters are arranged by the average pairwise IBD sharing between clusters using a Force Atlas graph layout, with outlines that indicate participant’s average local European ancestry. Catoctin individuals, displayed as squares, are projected based on the average IBD shared with each European cluster (shown as lines).

Next, we explored the rate of IBD sharing with members of the European cohort (i.e., 23andMe research participants with ≥99% European ancestry who indicated that either they or all four of their grandparents were born in Europe) to learn about the origins of the European-related ancestry that we observed in over half of the Catoctin individuals. We detect the highest rates of IBD among participants associated with Great Britain and Ireland (Fig. 4A, Table S12). Randomization testing confirms that we would be unlikely to detect the same number of IBD connections (n=467) with an identically sized sample (n=101,262) of randomly selected individuals from the European cohort (p-value<0.001) (Table S9). Much of the Irish-related signal is driven by connections to the individual from Burial 15, an adolescent male belonging to Family C, who, when projected onto a graph network of clusters that correspond to European geography again shares the most IBD with clusters of participants associated with the northern, western, and southeastern regions of the island of Ireland (Fig. 4B). Multiple other Catoctin individuals (in particular, those from Burials 2, 10, and 34) share broader connections with participants from Great Britain and Ireland (Fig. 4B, Fig. S11, Table S13).

### Distant and close relatives in the United States

**Fig. 5.**
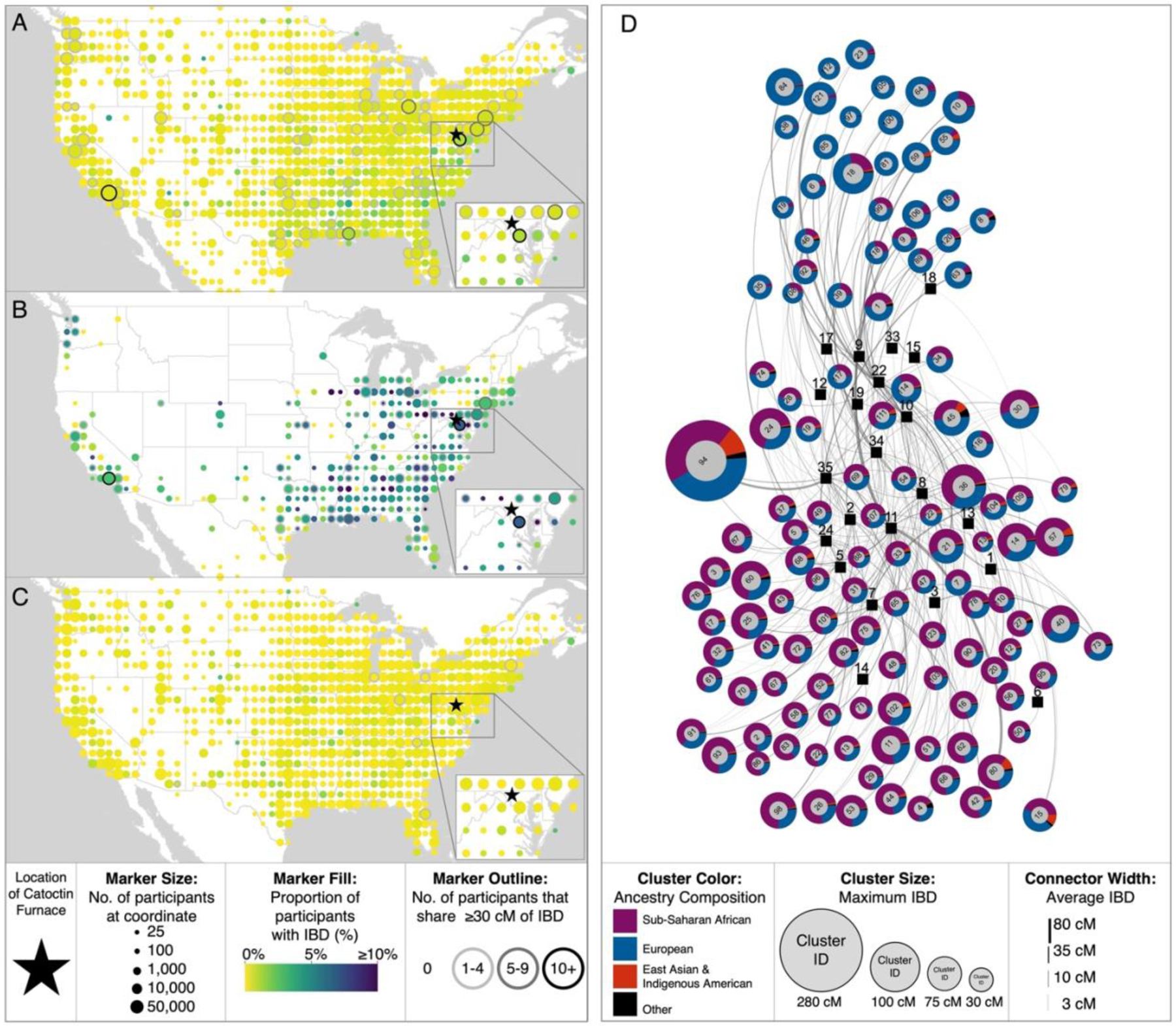
Geographic distribution of distant and close relatives of the Catoctin individuals among members of the US cohort. (**A**) Proportion of 23andMe research participants in the US cohort who share IBD with Catoctin. Only coordinates representing at least 25 participants after 80% downsampling are shown. Marker size corresponds to the number of participants associated with each coordinate, while color indicates the proportion of participants with shared IBD. Marker outlines indicate the number of participants at each coordinate who share ≥30 cM of IBD with one or more Catoctin individuals. The same information is shown for (**B**) participants with ≥5% Sub-Saharan African ancestry and (**C**) participants with ≥99% European ancestry. (**D**) IBD network of the closest present-day relatives of Catoctin individuals among 23andMe research participants. Circles represent modularity clusters consisting of close Catoctin relatives (sharing ≥30 cM of IBD) along with their relatives (sharing ≥100 cM with a close relative of a Catoctin individual). Clusters are outlined according to their average ancestry and arranged by the average pairwise IBD sharing between clusters using a Force Atlas layout. Catoctin individuals, displayed as squares, are projected based on the average IBD shared with each familial group (shown as lines).

When we considered IBD sharing between research participants in the US cohort (i.e., research participants who indicated that either they or all four of their grandparents were born in the US) and each of the Catoctin individuals separately, each historical individual exhibited a unique pattern of IBD sharing with respect to geography. However, when we considered the Catoctin individuals together, we observed the highest rates of sharing between Catoctin and research participants from the southern US (Fig. 5A, Fig. S12, Table S14). This signal resembles the geographic distribution of 23andMe research participants with Sub-Saharan African ancestry in the US (Fig. S13) and is therefore plausibly driven by a higher rate of IBD sharing in genomic regions with Sub-Saharan African ancestry. To address this source of bias, we restricted our analysis to research participants in the US cohort with ≥5% Sub-Saharan African ancestry (Fig. 5B, Table S15). This filtering strategy increases the rate of IBD sharing from 0.45% of all US participants to 4.25% (Table 1).

For participants included in this filtered dataset, we continued to observe elevated rates of IBD sharing with Catoctin in the southern US (including Maryland) (Fig. 5B). We confirmed via randomization test that we would be unlikely to detect the same number of IBD connections (n=2,034) with an identically sized sample (n=42,132) of randomly sampled individuals from the US cohort with ≥5% Sub-Saharan African ancestry (p-value <0.001) (Table S9). In contrast, when we filtered to include only participants in the US cohort with ≥99% European ancestry to focus on the genetic legacy of the admixed Catoctin individuals’ European ancestors along lineages with little to no African admixture, we did not detect any clear geographic patterns (Fig. 5C, Table S12).

When we focused on “close relatives” (referred to here as pairs of individuals who share at least 30 cM of IBD with a Catoctin individual, reflecting a relationship that is predicted to be 9^th^-degree or closer), we observed particularly pronounced connections to Maryland, identifying a total of 30 close relatives in the state (Table S16). A randomization test confirmed that we would be unlikely to identify 30 or more close relatives among an identically sized sample (n=19,972) of randomly selected individuals (p-value<0.001) (Table S17). These results suggest that at least some descendants of the Catoctin individuals or their family members remain in the Maryland area.

Next, we considered population substructure among the close relatives of the Catoctin individuals in the US using an IBD network approach. We analyzed 443 close relatives of the Catoctin individuals (≥30 cM of IBD) along with 4,385 of those participants’ closest relatives who share ≥100 cM of IBD—many of whom we hypothesize could be genetically related to the Catoctin individuals, even if we did not detect IBD sharing between them due to the high false negative rate of our approach—and identified 123 familial groups (Fig. 5D, Table S18). The positions of these familial clusters within the IBD network appear to be primarily correlated with the relative proportions of European and Sub-Saharan African ancestry detected among each cluster’s members, likely reflecting the ancestry of the individual IBD segments shared with the Catoctin individuals. In most cases, the familial groups do not appear to be correlated with geography. However, notably, all members of familial group 36 who provided location information have ties to Maryland, providing further evidence that Catoctin descendants and close relatives remained in or returned to Maryland after emancipation.

While elevated rates of IBD sharing are particularly evident in the Maryland region, we also identified other regions of the US with an enrichment of Catoctin close relatives. For example, the maximum amount of IBD (280 cM) is observed among a set of individuals from Southern California. This amount of sharing (corresponding to ~4% of the autosomal genome) is consistent with a 5^th^-degree relationship to the individual from Burial 35 based on maximum likelihood predictions (although the actual relationship may differ by a few degrees in either direction). Individuals with this amount of IBD sharing are likely either direct descendants of those buried at Catoctin or direct descendants of very close relatives of the Catoctin individuals (given that the Catoctin individuals likely lived at least five generations before most of the research participants included in this study) (Box 1, Table S7).

**Fig. 6.**
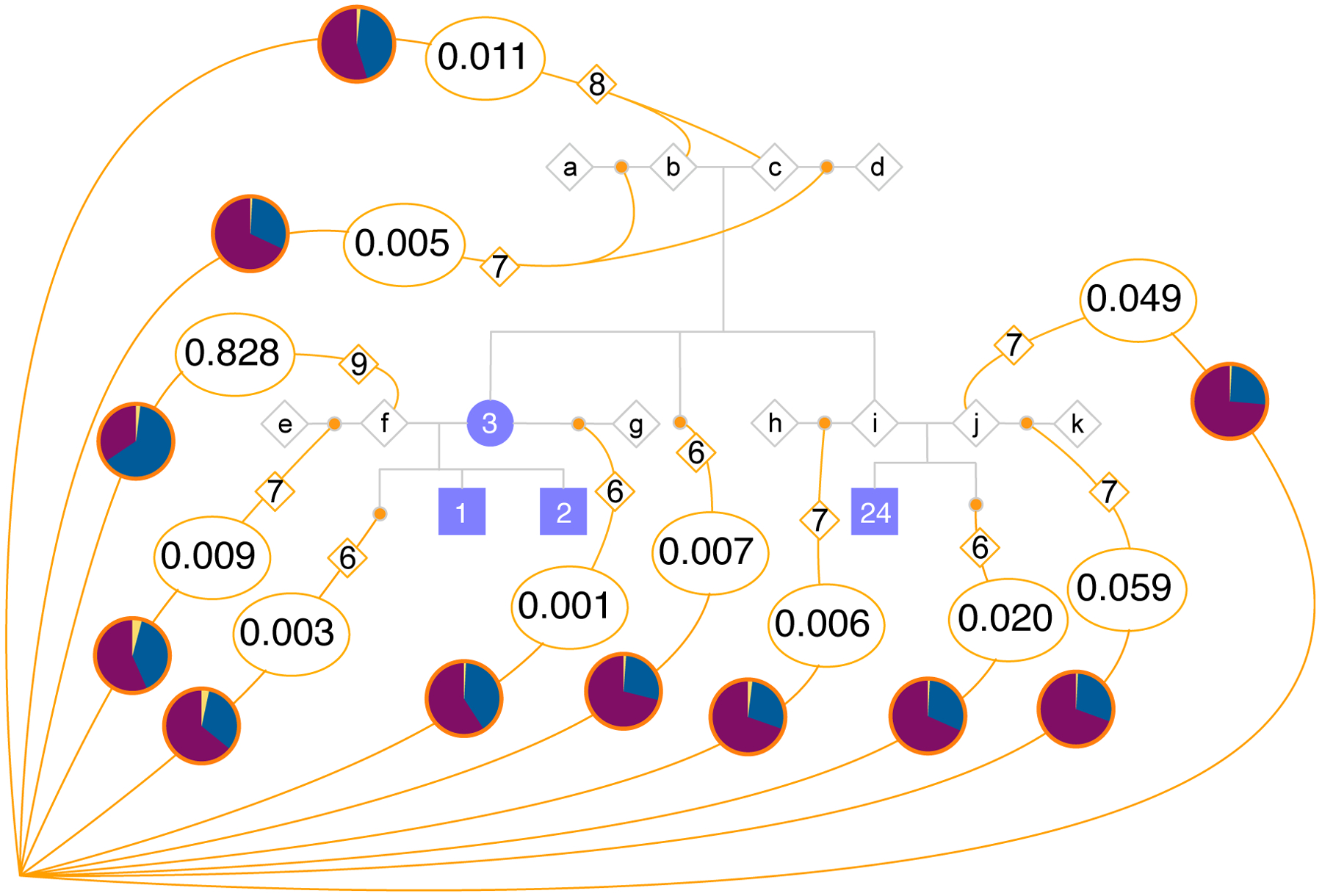
Connections between Catoctin Family A and modern pedigrees. The pedigree for Catoctin Family A is shown with blue-shaded individuals connected by gray lines. Open gray diamonds indicate un-genotyped individuals, some of whom must have existed (i.e., **b**, **c, f**, **i**, and **j)**, whereas others (i.e., **a**, **d**, **e**, **g**, **h**, **k**) are inferred to have existed. The large purple triangle represents all present-day pedigrees composed of 23andMe research participants, and the probability distribution of how these pedigrees connect to the historical pedigree is inferred. Orange dots indicate all possible points of connection of present-day pedigrees to the historical pedigree. Numbers in ovals give the percentage of present-day pedigrees whose most likely connection was to a given point on the historical pedigree. Numbers in orange diamonds indicate the average degree of a lineage connecting to a particular point. Pie charts show the average European (blue), Sub-Saharan African (purple), and Indigenous American (yellow) ancestry (normalized to sum to one) of individuals in pedigrees whose most likely point of connection was through the respective lineage leading to the present day.

To reconstruct pedigrees describing the connections shared between the Catoctin individuals and their closest genetic relatives in the 23andMe cohort, we used a modified version of the tool Bonsai (36, 43). The informed consent process for participation in 23andMe research requires strict protection of research participant anonymity, which means full pedigrees cannot be published. Instead, we highlight the ways in which 8,721 independent pedigrees (636 of which contain more than one research participant) are connected to Catoctin Family A (Fig. 6, Table S19). We found no cases where the most likely connection was via a direct descendant of individuals from Burials 1, 2, or 24, consistent with our expectations since these individuals died during childhood. The most probable path for most (83%) of the pedigrees we considered connects through an ancestor of the unsampled father of individuals from Burials 1 and 2 (referred to as un-genoyped individual *f* in Fig. 6). We find that pedigrees that include research participants who have at least twice as much Indigenous American ancestry as Sub-Saharan African ancestry were significantly more likely to connect through this unsampled individual than through other Catoctin individuals (p < 10^−6^; calculated via a permutation test with 10^6^ replicates) (Supplementary Text S6), consistent with our earlier prediction that this unsampled individual had some Indigenous American ancestry.

Most inferred connections extend upward in pedigrees generated for Families A, C and D (meaning that they connect through an ancestor of one or more members of the family, and therefore do not involve a direct descent relationship) (Figs. 6 and S14, Table S19). This is consistent with expectations, as individuals who lived in the last few hundred years are expected to have far more living collateral relatives than direct descendants. Notably, lineages that extend upwards from the Catoctin pedigrees tend to involve research participants who have more European ancestry than those lineages that extend downward. This suggests that a relatively large fraction of the connections that we identified involve ancestors of members of Family A with European ancestry, which in part may reflect biases in the 23andMe cohort where European ancestry is overrepresented.

These results demonstrate the power of our IBD-based approach to identify connections between historical and present-day individuals. In the future, by obtaining additional informed consent from research participants it may be possible to present more complex pedigrees that include direct descendants of historical individuals using this approach.

## Biologically significant variants

We considered sites in the Catoctin individuals’ genomes that might shed light on their physical traits and health (Table S20, Fig. S15). However, we caution that these results are based on low coverage data, and further work is required to conclusively infer genotypes at these positions.

For three Catoctin individuals, we identified copies of only the causal A allele at the genetic position (*rs334*/*i3003137*) which is associated with protection against malaria in the heterozygous form and sickle cell disease—a red blood cell disorder that causes pain and increased susceptibility to infection and early mortality (44)—in individuals with two copies of the allele. Although we only have limited coverage at this position for each of these three individuals (range: 1–5x coverage), making it impossible to confidently determine their genotypes, we note that two individuals (Burials 17 and 19) are siblings who died during early childhood. If they had sickle cell disease, this may have contributed to their early mortality.

We also observed four individuals with at least one copy of the causal T allele at another genetic position (*rs1050828*) that provides protection against malaria in genetically female individuals who carry one copy of this allele on only one of their X chromosomes. In genetic males, who are hemizygous, and in genetic females who are homozygous for the T allele at this position, it is associated with G6PD deficiency (45). Both variants occur at elevated rates in populations with ancestry from Sub-Saharan Africa where malaria has historically been endemic (46). Understanding how the frequency of these alleles may have changed in African American populations over time is a question of great interest (47, 48), which may be informed by ancient DNA data as more historical African Americans are sequenced.

## Discussion

This study demonstrates the power of genetic analyses to uncover previously unknown information about the family structure and ancestry of historical individuals, and to connect them with living relatives. For Catoctin Furnace, this research is a critical step toward identifying a larger descendant community, one of the main goals expressed by stakeholders from CFHS, AARCH, and the two Catoctin descendant families recently identified using historical documents and genealogical data. By developing an approach to identify genetic connections between historical individuals and their present-day relatives, DNA can be a significant means through which these relationships can be re-constructed.

At least 15 of the Catoctin individuals can be clustered into five genetic families, providing insight into the social structure of African Americans at this early industrial site. Historical records suggest that iron works enslavers often kept families together to benefit from a sustained knowledge transfer between generations. It was also believed that this practice minimized the likelihood of revolt or escape caused by family separation (49–51). The majority of the genetic connections we identified were between first-degree relatives, usually mothers and children. No fathers were identified, nor did we find families with representation of three or more generations. There were at least 11 individuals who appeared unrelated to others buried in the cemetery. These results may therefore indicate that, in practice, families did not remain together at this iron works. One possible explanation is that partners may have been sought outside of the Catoctin village. However, additional consideration must be given to how the types of genetic relationships we observed may be biased by incomplete excavation combined with burial patterning at the site, which was likely influenced by personal choice and/or imposed religious practices. For instance, from first-hand accounts recorded in journals and diaries, we know that some African American funerals at Catoctin were conducted by Moravian ministers (52, 53). The Moravians prescribed burial protocols in which families were not interred together, instead, they were buried in “choirs,” separated by marital status, age, and gender (54). This tradition introduces the possibility that married men were buried elsewhere in the cemetery, and therefore not sampled as part of this study (54). It was only through the co-analysis of aDNA and archival Moravian diaries that this unique aspect of burial and demography at Catoctin could be considered.

The genetically inherited conditions identified in this study (sickle cell anemia and G6PD deficiency) provide additional insight into the health and well-being of the Catoctin African Americans. Although these results are tentative and require more refined analyses, they offer insight and potential future avenues by which to explore the remains of deceased humans recovered from archaeological contexts.

At the population level, the Catoctin individuals have diverse ancestry, with clear genetic links to Africa, Europe, and the Americas. Most individuals have primarily Sub-Saharan African ancestry, with the strongest ties to present-day peoples in Senegambia and West Central Africa, a region that primarily encompasses present-day Angola and the Democratic Republic of the Congo. These findings correlate with historical records that show that slave ships originating from Senegambia (particularly before the end of the 18^th^ century) and West Central Africa accounted for the highest disembarkation rates in Maryland over the course of the transatlantic slave trade, while lower disembarkation rates were recorded from intervening coastal regions and the Caribbean (Table S21) (55). However, records of the intra-American slave trade, which was responsible for the arrival of many enslaved African Americans to Maryland, are more limited, making it challenging to infer what the most likely major sources of African ancestry in Maryland might have been based on historical records alone. According to the transatlantic slave trade database, ships departing from Senegambia and West Central Africa account for 15.8% and 20.8% of overall disembarkations in North America (55). Notably, the most common sources of African ancestry among the Catoctin individuals do not align with larger trends in the greater Chesapeake region, where the highest rate of disembarkation was from the Bight of Biafra–home to people of Igbo and Yoruba ancestry (55).

The Catoctin individuals were less likely to have genetic connections to multiple distinct African ethnolinguistic groups than research participants with substantial (≥50%) Sub-Saharan African ancestry and longstanding ties to the US (i.e., four grandparents born in the US), likely reflecting admixing between individuals with diverse African ancestries that occurred among enslaved African Americans and their descendants over time, following forced migration to the Americas (22, 56).

Due to the overrepresentation of research participants with European (particularly British and Irish) ancestry in the 23andMe cohort, we have even more power to localize the European ancestry of the Catoctin individuals. Eight individuals exhibit connections to Great Britain and/or Ireland. Differing frequencies of European associated maternal and paternal haplogroups observed at Catoctin indicate that their European ancestry was likely introduced through a sex-biased process almost certainly driven by rape of enslaved women as part of the gender-based sexual violence inherent in the US’s system of chattel slavery (38). We also observed a single European-associated mt haplogroup (in an individual who we estimate to have over 50% European ancestry). This may be one of numerous examples present in the historical record of enslaved or free Black men having children with white women (often indentured servants) (57).

Another objective of this study was to explore the possibility of identifying direct descendants using DNA. We identified 41,799 research participants with genetic connections to the Catoctin individuals. In many cases, it was possible to construct detailed genetic pedigrees that directly link 23andMe research participants to the historical individuals from Catoctin, either as direct descendants or, most commonly, as collateral relatives with a shared common ancestor. While most of these connections are very distant, we identified over 500 relatives who share ≥30 cM of IBD, reflecting a maximum likelihood estimate of 9^th^-degree relationship or closer.

It has been suggested that Catoctin’s enslaved workers were sold and transported to more southern states when the furnace transitioned to white wage labor (4). During the early 19^th^ century large numbers of enslaved individuals were sold from Mid-Atlantic states and transported south (58). While we observe strong connections to Maryland that suggest that this was unlikely to be the fate of Catoctin’s entire enslaved population, we do observe small clusters of close relatives throughout the US, including in the South.

The methodological approach we present in this study can be applied to the remains of deceased humans from other sites and contexts, offering a new scientific tool for individuals and descendant communities seeking greater knowledge of their ancestors, as well as archaeologists, bioarchaeologists, historians, genealogists. Museums and universities that steward the remains of deceased humans now have an additional means by which to identify past individuals and potentially link them to biological descendants. This work can currently only be done in partnership with organizations with access to massive, genetically diverse, re-contactable research cohorts, such as those maintained by genetic-ancestry companies. These partnerships will require conversations on the ethical implications and consequences of this work, particularly how to avoid reinforcing the biologization of identity (37). For researchers, this study represents advances in scientific methodology, but the impact may be even greater for those seeking connections to their past.

## Materials and Methods Summary

We sampled aDNA from the temporal bones of 27 Catoctin individuals, using a minimally destructive cranial based drilling approach when sampling from intact skulls (59). We generated double stranded, partially uracil-DNA glycosylase (UDG) treated DNA libraries (60–64). Before sequencing, we enriched the libraries for DNA aligning to the mitochondrial genome and 1.2 million positions in the nuclear genome using a capture-based approach (23–26). Following bioinformatic processing, all 27 DNA samples were deemed suitable for analysis, although the data for two individuals was subjected to damage restriction based on slightly elevated MT (26) or X-chromosome (65) contamination rates. For each individual, we inferred genetic sex (66) and uniparental haplogroups (67) and estimated the proportion of African, European and Indigenous American-related ancestry by comparing to publicly available datasets (68–71). Diploid genotypes were imputed using GLIMPSE (72) with the 1000 Genomes project phase 3 dataset (68) as a reference panel. After filtering and rephasing the data (73), we searched for IBD (41) between the imputed Catoctin individuals and 9,255,493 participants (elsewhere referred to as the “23andMe cohort”) who had been genotyped by 23andMe, Inc., a consumer personal genetics company, and provided informed consent to participate in research by July 28th, 2020. Summary statistics were generated describing these IBD connections for cohorts of research participants that were created based on each research participant’s genetic ancestry (as determined by the tool Ancestry Composition) and answers to 23andMe survey questions about their birth and grandparent birth locations. Genetic pedigrees were reconstructed using a modified version of the Bonsai pedigree inference algorithm (43). Finally, we counted the number of unique DNA sequences that overlap biologically significant positions in the genome for each Catoctin individual.

## Supplementary Material

SupplementaryNotes Materials and Methods
Supplementary Text S1 - Extended Ethics Statement
Supplementary Text S2 - The Return of Names
Supplementary Text S3 - Testing the application of IBD detection methods to imputed low coverage ancient DNA using simulated data
Supplementary Text S4 - Testing the performance of Ancestry Composition on imputed low coverage ancient DNA using simulated data
Supplementary Text S5 - Comparison of IBD Network results of Catoctin individuals with present-day African Americans
Supplementary Text S6 - Genetic connections to African Among Catoctin individuals and 23andMe participants in the US cohort with at least 50% Sub-Saharan African ancestry
Figs. S1 to S16
References 74–124

SupplementaryTables Tables S1 to S24, S1.1, S3.2 to S3.7, and S4.1

## Figures and Tables

**Table 1.** IBD shared between 22 Catoctin individuals and 23andMe participants

23andMe Participants included in group	Number of 23andMe participan ts in group	Proportion of 23andMe participants in group that share IBD with Catoctin Individuals	Number of IBD segments detected	Median Length of IBD segments detected (cM)*	Maximu m length of IBD segments detected (cM)*	Median total IBD in 23andMe participants with IBD detected (cM)*	Maximum total IBD shared with 23andMe participant s (cM)*
All participants	9,255,493	0.45%	55,342	10	60	9.9	280
Atlantic African cohort	3,304	2.27%	85	7.4	20	7.4	30
European cohort	226,384	0.22%	519	7.6	20	7.6	25
US cohort	2,993,165	0.51%	17,854	9.7	55	9.6	280
Members of US cohort with ≥5% Sub-Saharan African ancestry	192,880	4.25%	10,675	11	55	11	280
Members of US cohort with ≥99% European Ancestry	1,896,655	0.26%	5,123	8.0	30	8.0	40

Summary statistics describing frequencies and amounts of IBD shared between the 22 Catoctin individuals with >0.5x coverage and subsets of the 23andMe cohort. Participants were included in the US cohort either [1] if all four of their grandparents were born in the US, or [2] if they were born in the US and their grandparent birth location information was either unavailable or their grandparents were born in multiple countries. Similarly, members of the Atlantic African and European cohorts were determined using grandparent or participant birth location, with the additional requirement of ≥95% Sub-Saharan African ancestry or ≥99% European, respectively. Individual level results for each Catoctin individual are available in Table S5.

* Values are rounded based on the magnitude of IBD sharing as follows: values >100 cM are rounded to the nearest ten, values between 30–100 cM are rounded to the nearest five, values between 10–30 cM are rounded to the nearest integer, and values <10 cM are rounded to one decimal place).

## Data Availability

Unaligned and aligned sequences for the 27 Catoctin individuals were originally reported in Harney et al. (13) and are available from the European Nucleotide Archive under accession number PRJEB52230. Genotype files for pseudo-haploid and imputed versions of the dataset are available at https://reich.hms.harvard.edu/datasets. There are restrictions to the availability of 23andMe genotype data due to 23andMe consent and privacy guidelines. 23andMe agrees that the publication coauthors will rerun the comparison of historical genomic data against customer genetic data upon request by other academic and nonprofit researchers on reasonable terms to enable the results of the research activities to be replicated for at least seven years after publication or for as long as the coauthors are employed by, or otherwise affiliated with 23andMe in a capacity that allows them to rerun the analysis. Wherever possible, supplementary tables were included that report the summary statistics that were used to create figures that involved 23andMe datasets. Unless another comparable anonymizing approach was specified, these summary statistics were generated with the requirement that in all reported results, any 23andMe research participant must be indistinguishable from at least four other research participants included in the dataset. When meaningful, we repeated analyses performed on the 23andMe dataset using only the 1000 genomes and/or African American imputation panel datasets and reported these results.

## Box 1. Interpreting DNA shared between historical and present-day individuals.

The Catoctin Furnace African American Cemetery was in use between 1774–1850 (midpoint: 1812), while the average birth year of 23andMe research participants with genetic connections to Catoctin is 1974 (Table S22). The average separation between the death of the historical individuals and birth of present-day genetic relatives is ~162 years (although it is possible that as few as 70 years may have elapsed since the date of death of some of the latest Catoctin burials and the oldest research participants). The average age of reproduction among humans is 28–30 years, therefore the most likely number of generations separating the Catoctin individuals from their living relatives is about five to six generations (Fig. B1a).

The vast majority of the genetic relatives that we detected are not direct descendants of the Catoctin individuals. Instead, they are collateral relatives (i.e., relatives who are not direct ancestors or descendants) (Fig. B1b). To determine the likelihood that someone shares a direct descendant relationship with a Catoctin individual, we must consider the age of the two individuals and the amount of identical DNA (or IBD) that they share. Relatives who share very little IBD, but who lived close together in time (as is the case for most of the connections we identified), are less likely to share a direct descendant/ancestor relationship.
