# Supplementary material for: The Genetic Legacy of African Americans from Catoctin Furnace
Source: Science. Author manuscript; Available in PMC 2024 Mar 22. (PMC10958645; doi:10.1126/science.ade4995)
Supplement: SupplementaryNotes — Materials and Methods Supplementary Text S1 - Extended Ethics Statement Supplementary Text S2 - The Return of Names Supplementary Text S3 - Testing the application of IBD detection methods to imputed low coverage ancient DNA using simulated data Supplementary Text S4 - Testing the performance of Ancestry Composition on imputed low coverage ancient DNA using simulated data Supplementary Text S5 - Comparison of IBD Network results of Catoctin individuals with present-day African Americans Supplementary Text S6 - Genetic connections to African Among Catoctin individuals and 23andMe participants in the US cohort with at least 50% Sub-Saharan African ancestry Figs. S1 to S16 References 74–124 [file NIHMS1962424-supplement-SupplementaryNotes.pdf]

This is the author's version of the work. It is posted here by permission of the AAAS for personal use, not for redistribution. The definitive version was published in Science on August 4th, 2023, DOI: 10.1126/science.ade4995.

Supplementary Materials for

The Genetic Legacy of African Americans from Catoctin Furnace

Éadaoin Harney, Steven Micheletti, Karin S. Bruwelheide, William A. Freyman, Katarzyna Bryc, Ali Akbari, Ethan Jewett, Elizabeth Comer, Henry Louis Gates Jr., Linda Heywood, John Thornton, Roslyn Curry, Samantha Ancona Esselmann, Kathryn G. Barca, Jakob Sedig, Kendra Sirak, Iñigo Olalde, Nicole Adamski, Rebecca Bernardos, Nasreen Broomandkhoshbacht, Matthew Ferry, Lijun Qiu, Kristin Stewardson, J. Noah Workman, Fatma Zalzala, Shop Mallick, Adam Micco, Matthew Mah, Zhao Zhang, 23andMe Research Team, Nadin Rohland, Joanna L. Mountain, Douglas W. Owsley, David Reich

Correspondence to: eadaoinh@23andme.com (E.H.); jlmountain@gmail.com (J.M.); owsleyd@si.edu (D.W.O.); reich@genetics.med.harvard.edu (D.R)

This PDF file includes:

Materials and Methods
Supplementary Text S1 to S6
Figs. S1 to S16
References 74-124

Other Supplementary Materials for this manuscript include the following:

Tables S1 to S24, S3.2 to S3.7, and S4.1

This is the author's version of the work. It is posted here by permission of the AAAS for personal use, not for redistribution. The definitive version was published in Science on August 4th, 2023, DOI: 10.1126/science.ade4995.

Materials and Methods

In 1979–1980, the skeletons of 32 individuals were excavated from the Catoctin Furnace African American cemetery in Thurmont, Maryland, in advance of planned highway construction and were transferred into the custody of the NMNH. Sampling of 27 skeletons for ancient DNA was authorized by the NMNH Department of Anthropology Collections Advisory Committee.

A report describing the generation of the genetic data was submitted to the NMNH Department of Anthropology Collections Advisory Committee (*13*) and the genetic data was deposited in the European Nucleotide Archive under accession number PRJEB52230 after three years had elapsed following the original date of sampling, in accordance with the review committee's sampling policies. For completeness, we reprise here a description of how the DNA data was prepared.

Ancient DNA Sequencing:

We performed all ancient DNA sampling work in dedicated ancient DNA facilities at Harvard Medical School, following standard guidelines for ancient DNA laboratory setup and cleaning.

We sampled bone powder from the petrous portion of the temporal bone (*74*, *75*) from a total of 27 Catoctin individuals. When sampling from intact skulls, we used a minimally destructive cranial base drilling approach (*59*). We extracted DNA from ~37 mg of bone powder using published methods (*60–62*) and created dual-barcoded, double stranded DNA libraries treated with a partial uracil-DNA glycosylase (UDG) approach that reduces characteristic ancient DNA damage (*63*, *6*).

This is the author's version of the work. It is posted here by permission of the AAAS for personal use, not for redistribution. The definitive version was published in Science on August 4th, 2023, DOI: 10.1126/science.ade4995.

To maximize the amount of endogenous human DNA sequenced, we used an in-solution targeted enrichment capture approach. We enriched for sequences that overlap the mitochondrial genome and ~1.24 million (1240k) single nucleotide polymorphisms (SNPs) from the nuclear genome (*24–26*, *76*). Following enrichment, we added unique seven base pair long indexing barcodes to each molecule in the library and performed paired-end sequencing using either an Illumina HiSeq10 or NextSeq500 instrument with 2x101 or 2x76 cycles, respectively, and with 2x7 cycles to read the library indices. We also performed a relatively small amount of shotgun sequencing for each library, which we used to assess DNA quality.

We trimmed the molecular adapters and barcodes from sequenced reads, and merged paired-end reads (requiring an overlap of 15 base pairs with up to 3 mismatches in low quality bases (<20) or 1 mismatch of a high quality base (≥20) using custom software (https://github.com/DReichLab/ADNA-Tools). We mapped the merged sequences to both the mitochondrial (mt) consensus sequence (RSRS) (*77*) and the human reference genome (version hg19) using *samse* in BWA (v0.6.1) (*78*). We identified duplicate molecules using Picard MarkDuplicates (http://broadinstitute. github.io/picard/), defining reads with identical start and end positions, orientation, and identical DNA barcodes as duplicates, and retaining only the highest quality duplicate sequence.

We assessed ancient DNA authenticity and considered the following criteria when deciding if the data were suitable for analysis. Typically, requiring a minimum rate of 3% cytosine-to-thymine substitutions at the 5’ end of each molecule is recommended to establish ancient DNA authenticity (*63*). However, based on the relatively recent age of the Catoctin skeletal samples, versus the

considerably more ancient samples upon which this recommendation was based, we considered all of the Catoctin individuals (minimum 2.3% terminal damage rate) to have sufficient damage rates to be suitable for analysis (*63*). Next, we quantified the mt contamination rate using contamix v1.0-12 (*26*), requiring libraries to have 95% confidence intervals for their inferred contamination rate to be entirely greater than 5%. Additionally, we used ANGSD (*65*) to estimate contamination on the X-chromosomes of genetic males, requiring contamination rates below 3%.

The libraries generated from two individuals (Burials 13 and 25) had somewhat elevated levels of estimated contamination when we considered the upper bounds of the 95% confidence intervals of the mt match to consensus and ANGSD contamination rates. Therefore, we restricted to sequences that showed evidence of having ancient DNA damage, using the damage restriction approach described in PMDtools (*79*). The damage restricted version of each library passed all authenticity metrics and was used to create pseudo-haploid genotype calls (described later) used for preliminary population genetic analyses. However, since we do not expect such low rates of contamination to significantly impact the accuracy of imputed genotype calls, all sequences were used during the imputation process.

Imputation

Imputation was performed using GLIMPSE (*72*) (v1.0.0) using the 1000 Genomes project phase 3 dataset as the reference panel (*68*). Diploid genotype calls were generated using bcftools mpileup (v1.10.2). All the autosomal biallelic SNPs and indels in 1000 Genomes project phase 3 have been imputed. Only biallelic genotype likelihoods for SNPs, generated by mpileup were used as input to build the phasing and imputation model, and genotype likelihoods for indels were ignored

This is the author's version of the work. It is posted here by permission of the AAAS for personal use, not for redistribution. The definitive version was published in Science on August 4th, 2023, DOI: 10.1126/science.ade4995.

because of more severe reference bias. Unless otherwise noted, the data were filtered to remove genotype calls with an estimated maximum genotype posterior below a minimum threshold (henceforth referred to as the "max (GP) threshold") of 0.95.

Genetic Sex and Uniparental Haplogroups

We determined the genetic sex of each individual by calculating the ratio of the number of shotgun sequences that align to the X chromosome versus the Y chromosome. Individuals for whom we detected a ratio of approximately 1:1 shotgun sequences aligning to the X and Y chromosomes were assigned a genetic sex of male, while individuals for whom we detected a ratio of approximately 1:0 were assigned a genetic sex of female (*66*). To identify mitochondrial haplogroups, we considered reads that aligned to the RSRS mitochondrial genome with MAPQ ≥ 30 and base quality ≥ 20. Haplogroups were determined with haplogrep2 (*67*), using Phylotree version17. Y-chromosome haplogroups were called from reads that aligned to the Y-chromosome with MAPQ ≥30 and base quality ≥30. The most derived mutation for each individual was used to determine the Y-haplogroup, using the nomenclature defined by the International Society of Genetic Genealogy (ISOGG) (http://www.isogg.org) version 14.76 (April 2019). Assigned genetic sex, mt and Y haplogroups for each individual are reported in Fig. 1B and Table S1.

Genetic relatedness between Catoctin Individuals

We identified genetic relatives of first, second, and third degree using a previously described method based on comparing the rate of matching across individuals to the rate of matching of the maternal and paternal chromosomes of the same individual (*80*). We identified 15 related individuals, belonging to 5 distinct families. Another individual (Burial 28) did not have sufficient coverage to conclusively determine whether they should be included in genetic Family B. Using

This is the author's version of the work. It is posted here by permission of the AAAS for personal use, not for redistribution. The definitive version was published in Science on August 4th, 2023, DOI: 10.1126/science.ade4995.

the estimated degree of relatedness, genetic sex, uniparental haplogroups and age of death of each individual we further resolved the relationship shared between each pair of related individuals, as shown in Fig. 1B. While IBD comparisons between the Catoctin individuals (described later) were not used to determine these genetic relationships, the results of this analysis are consistent with these predicted relationships considering that we expect to underestimate the total amount of IBD shared between historical individuals (Fig. S16).

Comparison to publicly available data

We determined pseudo-haploid genotype calls for the non-imputed dataset by randomly sampling a single sequence to represent each position. We merged previously reported, publicly available, genome-wide data from present-day individuals from 12 populations (BantuSA.SDG, CHB.SG, ESN.SG, FIN.SG, GBR.SG, GWD.SG, Khomani_San.DG, Mandenka.SDG, Mbuti.SDG, MSL.SG, Pima.SDG, and YRI.SG) (*68–71*) with the newly generated pseudo-haploid dataset containing 27 Catoctin individuals. We refer to this dataset as "the public dataset." The resulting merged dataset contained information at 1,233,013 SNPs. We also created a version of this publicly available dataset using the imputed genotype calls for the Catoctin individuals (filtering out all genotype calls with a maximum genotype posterior below 0.95), to show the impact of imputation on the Catoctin dataset. This dataset contained information at 1,083,703 SNPs.

qpAdm estimates of mixture proportions

We used the software qpAdm (*81*) (version 960) to estimate the proportion of ancestry that derived from African, European and Indigenous American sources for each Catoctin individual, using default parameters and the option "allsnps:YES". Since the purpose of this analysis was to broadly estimate ancestry proportions and not to identify the best possible model for each individual's

ancestry, we selected a single population to represent each ancestry type: YRI.SG (African), GBR.SG (European), and Pima.SDG (Indigenous American). An advantage of qpAdm is that it does not require access to reference data from individuals from the true source population to give unbiased and accurate results; instead, it works well if the set of samples used to represent each ancestry type are descended from the same ancestral population (perhaps distantly in time) as the true source population. We selected four reference populations that are differentially related to each of these source populations and that we considered unlikely to be more closely related to the Catoctin individuals than chosen source populations: Mubti.SDG, Khomani_San.DG, CHB.SG, FIN.SG. We considered all models with p-values >0.01 to be plausible. Additionally we considered models that assigned ancestry proportions outside the range of 0–1 to be plausible if the assigned proportions were within 3 standard errors of this range. Applying this approach to the non-imputed dataset, we could plausibly model the ancestry of 22 of the 27 Catoctin individuals tested. Two of the individuals whose ancestry could not be plausibly modeled using this approach were assigned over 100% YRI.SG ancestry, indicating that they likely have fully African ancestry but that YRI.SG is not a good proxy for this ancestry with respect to the reference populations included in the model. We could plausibly model the ancestry of all Catoctin individuals using this approach when using the imputed dataset.

ADMIXTURE

We used the clustering tool ADMIXTURE (*82*) to assign the ancestry of the Catoctin individuals and representative African (GWD.SG, Mandenka.SDG, MSL.SG, YRI.SG, ESN.SG and BantuSA.SDG), European (GBR.SG) and Indigenous American (Pima.SDG) populations to *k* theoretical ancestral populations. We pruned SNPs in linkage disequilibrium using PLINK with

parameters --indep-pairwise 200 25 0.4 and performed ADMIXTURE analysis on the remaining 1,095,120 SNPs for values of *k* between 2 and 10, with 10 replicates for each value of *k*. The highest likelihood replicate at each *k* was retained and we display results for *k*=4, which was the most visually informative for distinguishing broadly between African, European and Indigenous American ancestry in the Catoctin individuals. We also repeated this analysis using the public dataset with imputed versions of the Catoctin individuals. The pruned dataset consisted of 1,021,850 SNPs.

PCA

We performed principal components analysis (PCA) using smartpca (*83*) with default parameters in addition to the settings lsqproject:YES, numoutlier:0, shrinkmode:YES and ellconf: 0.95. We projected the Catoctin individuals onto a PCA plot created using representative African (YRI.SG), European (GBR.SG) and Indigenous American (Pima.SGD) populations. This PCA was designed to reveal a cline of European-related ancestry among self-identifying African Americans (ASW.SG). The PCA plot was created using both the non-imputed and imputed versions of the Catoctin dataset to show the impact of imputation.

DATES

We estimated the date of European and African admixture for each of the Catoctin individuals using the tool DATES (*84*) using the non-imputed dataset, with default parameters in addition to the settings mincount: 2 and minparentcount: 0. The populations YRI.SG and GBR.SG were used to represent the European and African admixture sources.

This is the author's version of the work. It is posted here by permission of the AAAS for personal use, not for redistribution. The definitive version was published in Science on August 4th, 2023, DOI: 10.1126/science.ade4995.

23andMe dataset

We compared the Catoctin dataset with data from 9,255,493 participants (elsewhere referred to as the "23andMe cohort") who had been genotyped by 23andMe, Inc., a consumer personal genetics company, by July 28th, 2020. Participants provided informed consent and participated in the research online, under a protocol approved by the external AAHRPP-accredited IRB, Ethical & Independent Review Services (E&I Review). Participants were included in the analysis on the basis of consent status as checked at the time data analyses were initiated. The name of the IRB at the time of the approval was Ethical & Independent Review Services. Ethical & Independent Review Services was recently acquired, and its new name as of July 2022 is Salus IRB (https://www.versiticlinicaltrials.org/salusirb). We also included data from the 1000 Genomes Project (*68*) (Table S24), the Human Genome Diversity Project (*69*), and previously published data from people from Angola (*22*), the Democratic Republic of the Congo (*85*) and Sierra Leone (*86*), in addition to Khoe-San speaking people (*87*) in these comparisons.

We considered a participant to be associated with a particular geographic location based on their answers to questions about where they and their grandparents were born. In cases where all four grandparents were born in the same location, we prioritized this information over participant birth location. Additionally, in cases where participant birth location and grandparent birth location was the same, and where we had finer resolution for participant birth location (*i.e.*, we know that all four grandparents and the participant were born in the same country and we also have information about the participant's birth state) we prioritized birth location information. We grouped participants associated with the US in the "US cohort." We grouped participants associated with European countries (including the following countries, as denoted by their ISO2 country codes:

This is the author's version of the work. It is posted here by permission of the AAAS for personal use, not for redistribution. The definitive version was published in Science on August 4th, 2023, DOI: 10.1126/science.ade4995.

AD, AL, AT, AX, BA, BE, BG, BY, CH, CZ, DE, DK, EE, ES, FI, FO, FR, GB, GG, GI, GR, HR, HU, IE, IM, IS, IT, JE, LI, LT, LU, LV, MC, MD, ME, MK, MT, NL, NO, PL, PT, RO, RS, RU, SE, SI, SJ, SK, SM, UA, VA, and XK) into a “European cohort,” with the additional requirement that participants in this cohort are assigned ≥99% European ancestry by Ancestry Composition. Similarly, we grouped participants associated with African countries (including the following countries, as denoted by their ISO2 country codes: AO, BI, CD, CM, CF, TD, CG, GQ, GA, KE, NG, RW, ST, TZ, UG, SD, SS, DJ, ER, ET, SO, BW, KM, LS, MG, MW, MU, MZ, NA, SC, ZA, SZ, ZM, ZW, BJ, ML, BF, CV, CI, GM, GH, GN, GW, LR, MR, NE, SN, SL, and TG) into an “African cohort,” requiring that participants in this cohort were assigned ≥95% Sub Saharan-African ancestry by Ancestry Composition. Finally we grouped participants associated with Atlantic African countries (including the following countries, as denoted by their ISO2 country codes: AO, CD, CF, CI, CM, GH, GM, GN, LR, NG, SL, SN, TG, and ZA) into an “Atlantic African cohort,” again requiring that participants in this cohort were assigned ≥95% Sub-Saharan African ancestry by Ancestry Composition.

Ancestry Composition

We performed ancestry prediction using 23andMe’s Ancestry Composition tool (*42*) for each of the 22 Catoctin individuals with >0.5x coverage, using a max (GP) threshold of 0.00, reporting results with a 90% confidence threshold. In Supplementary Text S4, we tested the performance of ancestry composition on an imputed test dataset of high coverage ancient individuals, which was originally described in Supplementary Text 3. We find that Ancestry Composition performs better when poorly imputed genotypes are retained (i.e. no max (GP) threshold is used) than when these genotypes are considered missing. We therefore rephased the unfiltered imputed Catoctin

This is the author's version of the work. It is posted here by permission of the AAAS for personal use, not for redistribution. The definitive version was published in Science on August 4th, 2023, DOI: 10.1126/science.ade4995.

genotypes against the panel of 23andMe research participants genotyped on the version 5 23andMe sequencing platform, as described previously, and performed Ancestry Composition analysis on the resulting dataset. We also found that while the broadest level Ancestry Composition assignments (e.g., Sub-Saharan Africa, East Asia & the Americas, West Asia & North Africa) were consistently assigned, even to the lowest coverage, imputed ancient DNA data, the more specific assignments (e.g., British & Irish versus Italian) were not reliably assigned. We therefore only considered the broadest level Ancestry Composition assignments for each Catoctin individual.

Identity-by-Descent

To search for portions of the genome that are identical by descent between the Catoctin individuals and members of the 23andMe cohort, we filtered out genotype calls that were assigned a max (GP) of less than 0.95. This threshold was chosen based on extensive testing of the performance of TPBWT comparisons between ancient individuals and 23andMe participants, described in Supplementary Text S3. After filtering, we then rephased the imputed data using EAGLE, with default settings and optional parameter --allowRefAltSwap. We created two rephased datasets using a reference panel of either 691,759 23andMe research participants at 454,507 SNPs or 706,995 23andMe research participants at 541,948 SNPs for subsequent analysis of participants genotyped on the versions 1-4 or version 5 23andMe genotyping platforms, respectively. Missing genotypes, including those that were filtered out when the minimum genotype posterior threshold was applied, were not re-imputed.

This is the author's version of the work. It is posted here by permission of the AAAS for personal use, not for redistribution. The definitive version was published in Science on August 4th, 2023, DOI: 10.1126/science.ade4995.

We ran TPBWT with default parameters, comparing the 22 Catoctin individuals with sequencing coverage of at least 0.5x and the 23andMe cohort. While TPBWT reports IBD segments as short as 3cM, we restricted our analyses to segments that exceeded minimum length thresholds. These thresholds were defined for each Catoctin individual based on average chromosomal coverage at 1240k sites to reduce the frequency of false positive IBD calls to ~10% in the shortest IBD segments that we consider (Supplementary Text S3). We used average chromosomal coverage at 1240k sites, rather than the overall coverage on 1240k sites (which does not consider coverage on each chromosome separately) to best match the coverage estimates used in the down-sampling tests described in Supplementary Text S3. References to "coverage" in this manuscript refer to average chromosomal coverage at enriched autosomal positions. For individuals with >2x average chromosomal coverage (n=5), we considered segments as short as 6 cM. For individuals with 1-2x average chromosomal coverage (n=10), we considered segments as short as 9cM and for individuals with 0.5-1x average chromosomal coverage (n=7), we considered segments as short as 10cM.

We interpreted IBD shared between the Catoctin individuals and participants by generating summary statistics that group participants based on their geographic location (provided by participants through surveys) and their ancestry composition (assigned by 23andMe's Ancestry Composition tool (*42*)). We generated metrics including the proportion of participants that shared IBD with the Catoctin individual(s) of interest, the average total IBD shared between participants and the Catoctin individual(s) of interest (where average is calculated across all participants, and only those participants who shared some amount of IBD with the Catoctin individual(s) of interest), the maximum amount of IBD shared between a single participant and Catoctin individual

(rounded to 1 digit when IBD sharing is <10cM, 0 digits when IBD sharing is between 10-100cM, and to the nearest 10 when IBD sharing exceeds 100cM), and the number of participants that shared at least 30 cM of IBD with the Catoctin individual (s) of interest. Results are reported for participants grouped based on associated geographic coordinates (rounded to the nearest integer) for participants in the US, European and African cohorts.

To ensure participant anonymity, coordinate level results are reported only for locations that have at least 25 associated participants. Additionally, we randomly downsampled our 23andMe cohort to include only results for 80% of participants to provide further anonymity. In the US and European maps, we show data only for participants who provided county or state level information, respectively, while in Africa we also include participants who provided only country level information, in which case we assigned them to the geographic centroid of the country.

Randomization Tests

We performed randomization tests in order to determine whether cases where elevated rates of IBD sharing with the Catoctin individuals among specific subsets of research participants were observed could be explained by random chance. In each case, we randomly sampled participants (with replacement) from the larger participant cohort of interest (e.g. participants in the US with over 5% Sub-Saharan African ancestry) to create 1000 random subsets of participants that were equal in size to specific subsets of participants of interest (e.g. participants with over 5% Sub-Saharan African ancestry from the southern US [i.e., Alabama, Arkansas, Delaware, Florida, Georgia, Kentucky, Louisiana, Maryland, Mississippi, North Carolina, Oklahoma, South Carolina, Tennessee, Texas, Virginia, West Virginia and the District of Columbia]). For each replicate, we

counted the number of participants in the subset who shared any IBD with a Catoctin individual, or at least 30 cM in the case of the Maryland randomization test. We calculated p-values as the proportion of sampled subsets in which more individuals shared matches to Catoctin than were observed among participants from the true subset of participants under consideration. We considered all p-values <0.01 to be significant.

IBD Networks

To identify Catoctin individuals' distant and recent connections to genetic groups, we first performed community detection using the Louvain Method (*88*). The Louvain Method uses a heuristic model to optimize modularity, a value that represents the number of connections inside proposed groups with respect to connections to outside groups. Optimizing this value results in a natural grouping of participants of a given cohort. In the context of this study, the Louvain Method determines genetic clusters of participants by placing individuals into groups that maximize IBD sharing within a group after assessing pairwise IBD shared between all individuals in the cohort. We applied this method to unrelated Atlantic Sub-Saharan Africans with ≥99% Sub-Saharan African ancestry (N = 2,807), unrelated Europeans with ≥99% European ancestry (N = 23,092), and a partially-related cohort of individuals that share >30 cM with any Catoctin individual (N = 4,828). Unrelated cohorts are filtered so that no two individuals share ≥700 cM, whereas partially-related cohorts contain no IBD filters. In each of the three cohorts, we identified the groupings of participants that maximize modularity and found any enrichment of geographical or ethnolinguistic terms within each group using survey answers provided by participants. After identifying the optimal clusterings, based on modularity, we determined the average amount of IBD each Catoctin individual shares with each of the groups. We used these data to display each

This is the author's version of the work. It is posted here by permission of the AAAS for personal use, not for redistribution. The definitive version was published in Science on August 4th, 2023, DOI: 10.1126/science.ade4995.

Catoctin individual's connections to each group in the form of a graph layout. To achieve this, we first arranged groups within each of the cohorts, using the Force Atlas layout(*89*). Force Atlas is an algorithm that situates groups (or nodes) in a graph using a physical magnetic model. In this case, groups with more IBD sharing will be attracted to one another and groups with less IBD sharing are repelled. Force Atlas runs until balance between repulsion and attraction is achieved, essentially illustrating the structure of groups via their IBD sharing. After Force Atlas was run on each of the participant cohorts, we independently ran Force Atlas between each Catoctin individual and the pre-arranged graph of participants, projecting Catoctin individuals onto the structure of each cohort, thus illustrating where Catoctin individuals physically fell into the structure of each cohort.

Pedigree Reconstruction

Pedigrees were inferred between Catoctin families and modern pedigrees using a modified version of the Bonsai pedigree inference algorithm that is used to reconstruct research participant pedigrees at 23andMe (*43*). The algorithm was modified to use likelihoods adjusted for low coverage individuals (Supplementary Text S6).

Biologically Significant Variants

We determined the number of reference and alternative alleles observed in unique reads that overlap positions of phenotypic interest, including those known to be associated with sickle cell anemia and G6PD deficiency. While sequencing coverage was not high enough for any of the Catoctin individuals to make diploid genotype calls, the presence of causal alleles that are associated with phenotypes of interest suggests that the Catoctin individuals were either homozygous or heterozygous at these sites

This is the author's version of the work. It is posted here by permission of the AAAS for personal use, not for redistribution. The definitive version was published in Science on August 4th, 2023, DOI: 10.1126/science.ade4995.

Supplementary Text S1: Extended Ethics Statement

S1.1 Permissions for this and prior related studies

The Catoctin Furnace cemetery was excavated during a Maryland state highway project in 1979-1980 and the remains were subsequently transferred to the care of the Smithsonian National Museum of Natural History (NMNH). Authorization for the ancient DNA analysis of 27 Catoctin individuals was provided by the NMNH Department of Anthropology Collections Advisory Committee. In fulfillment of DNA sampling requirements, the genetic data were made fully publicly available on European Nucleotide Archive after 3 years had elapsed after the initial date of sampling. A technical report that describes the methods used during sampling, data generation, and bioinformatic processing and that details the results of the genetic analyses that had already been publicly presented during the community outreach events described below was returned to the Collections Advisory Committee and posted publicly at the same time (*13*).

The Catoctin Furnace Historical Society, Inc. (CFHS) and the African American Resources Cultural and Heritage Society (AARCH) are considered organizations with a vested interest in the research, and were consulted throughout the study. Members of two families that can genealogically trace their ancestry to enslaved and free African Americans who labored at Catoctin Furnace were also consulted. Each group directly supported the decision to collaborate with the direct to consumer genetic testing company, 23andMe, Inc., in order to compare the DNA of the Catoctin individuals to research participants in the 23andMe cohort.

This is the author's version of the work. It is posted here by permission of the AAAS for personal use, not for redistribution. The definitive version was published in Science on August 4th, 2023, DOI: 10.1126/science.ade4995.

S1.2 Community outreach

The community outreach efforts included in this study built upon a foundation established by co-author Elizabeth Comer (current President of the Catoctin Furnace Historical Society). For further information about the history of CFHS please refer to https://catoctinfurnace.org/.

A major goal of CFHS is to identify a descendant community of the African American individuals buried at Catoctin Furnace. Despite extensive efforts to identify such a community, CFHS has only recently been successful in identifying one family who can trace their ancestry back to an enslaved person at Catoctin Furnace. They have also identified a family who can trace their ancestry to a free African American ironworker who labored at the furnace. The search for the broader descendant community was therefore one of the primary drivers of this genetic study.

Although identifying a descendant or direct biological kinship community for the enslaved and free African Americans buried at Catoctin Furnace is a goal for CFHS, multiple groups have served as community stakeholders for these individuals, including:

- **The Catoctin Furnace Historical Society, Inc. (CFHS)**. This society was established in 1972 and among its members are descendants of the paid laborers of primarily European ancestry who worked at (but did not own) the Furnace. Many of these descendants still reside in Thurmont, Maryland in dwellings associated with Catoctin Furnace and their lives are deeply intertwined with the village's history.
- **The African American Resources Cultural and Heritage (AARCH) Society, Frederick, Maryland**. AARCH was officially incorporated in 2009, and its mission is to

This is the author's version of the work. It is posted here by permission of the AAAS for personal use, not for redistribution. The definitive version was published in Science on August 4th, 2023, DOI: 10.1126/science.ade4995.

"identify, collect, preserve and exhibit the cultural objects, artifacts, and stories that tell and celebrate the unique history, culture, and heritage of African Americans in Frederick County, Maryland for the purpose of educating the general public and deepening our understanding of how the African American past can shape and enrich the present and the future." Members of AARCH have worked closely with CFHS to ensure that the portrayal and study of African Americans at Catoctin Furnace is done sensitively and appropriately.

- **Members of descendant families.** Historical research led by CFHS has recently identified two descendant families whose ancestry can be directly traced to enslaved and free African American ironworkers who labored at Catoctin Furnace.

Interaction with these stakeholder communities and others with an interest in the history of Catoctin Furnace has involved a variety of engagement events and activities, during which we shared findings from the study and gathered community feedback on the project. These events and activities include:

- **The Catoctin Furnace African American Cemetery Interpretive Trail.** Visitors to Catoctin Furnace (both in person and virtual) can learn about the role that enslaved and free African Americans played at Catoctin Furnace during its early history by participating in a self-guided tour that leads participants from the ruins of Catoctin's "Isabella" Furnace to an observation area that overlooks the site of the African American cemetery. Along the trail are 11 informational panels that describe the site and various aspects about the lives of the enslaved and free African American laborers at Catoctin. We contributed to panel 9 of the interpretive trail by reporting the genetic relationships identified among the 27

This is the author's version of the work. It is posted here by permission of the AAAS for personal use, not for redistribution. The definitive version was published in Science on August 4th, 2023, DOI: 10.1126/science.ade4995.

individuals included in this study. More information about the interpretive trail is available here: https://catoctinfurnace.org/african-american-cemetery/

- **Facial Reconstruction Unveiling Event.** In June of 2021, the CFHS held an event during which facial reconstructions that had been created of two African Americans buried at Catoctin were unveiled to members of CFHS and other interested members of the Frederick community. The evening included readings by Elayne Bond Hyman from her collection of poems, Catoctin SlaveSpeak, performances by Joseph Ngwa, a master ancestral drummer, and a reading of the names of the enslaved individuals at Catoctin, which paid tribute to the idea that "A person is not forgotten until his or her name is forgotten" (Supplementary Text S2). Also among the events of the evening were two talks in which representatives of our research team shared preliminary genetic results from our study, including a description of the genetic relationships that were identified, preliminary estimates of ancestry, and discussion of the possible identification of several individuals who may have been carriers of or had sickle cell disease. Media coverage of the evening is available in The Washington Post (*90*) and in The Frederick News-Post (*91*). Placards describing the results presented during this evening are now on display in the Museum of the Ironworker at Catoctin Furnace.
- **Tours of the NMNH Collections for Catoctin Stakeholders.** Co-authors affiliated with the NMNH hosted multiple tours of the NMNH collections for members of CFHS and AARCH, as well as students and teachers from the Silver Oak Academy, a school for at-risk youth in Keymar, Maryland affiliated with CFHS. The most recent of these tours occurred in April, 2022.

This is the author's version of the work. It is posted here by permission of the AAAS for personal use, not for redistribution. The definitive version was published in Science on August 4th, 2023, DOI: 10.1126/science.ade4995.

- **Screening of "America's Hidden Stories: Forged In Slavery."** CFHS and members of the study team helped to organize a screening of this Smithsonian Channel documentary in Frederick Maryland in June, 2022. The documentary spotlights the ongoing research into the lives and legacies of the Catoctin African Americans. Additionally, several members of the study team were interviewed about the genetics of the Catoctin individuals as part of the documentary.
- **Virtual meeting with members of the descendant families.** In June, 2022, members of the research team virtually met with representatives from the two descendant families to discuss the results of this study.

S1.3 Informed Consent Provided by Present-day Research Participants

In this study, we compared the Catoctin individual's genomes to that of present-day research participants whose genetic data was obtained from a variety of sources. Here, we outline the different datasets used and provide an overview of the informed consent that was provided with each of these datasets. In Table S1.1 we specify which datasets were used in each analysis and indicate whether the results of those analyses can be reproduced using publicly available data.

- **Non-23andMe datasets:**
  - **1000 Genomes Project**

    Individual's whose genetic data is included in the 1000 Genomes Project (*68*) dataset provided broad consent to allow individual level data generated from the samples that they provided to be made publicly available on online scientific databases, provided that no individual identifiers or medical information was associated with the samples. The 1000 Genomes dataset can be used for a variety

of purposes, including studying population history and relatedness, and it may be accessed by academic, commercial and government entities. The sampling protocol for all samples included in the dataset was required to be approved by local Internal Review Boards (IRB) or Research Ethics Committees (REC), while also fulfilling the requirements of the 1000 Genomes Samples and ELSI group. For more information about the 1000 Genomes Project consent process, see: https://www.internationalgenome.org/sample_collection_principles/.

- **The Human Genome Diversity Project**

  Like the 1000 Genomes Project dataset, individual's whose genetic data is included in the Human Genome Diversity Project (HGDP) dataset provided broad consent to allow individual level data generated from the samples that they provided to be made publicly available on online scientific databases. The HGDP dataset was generated with the intention of providing a resource that could be used to study human genetic variation, including studies of human evolutionary history and/or ancestry. In this study, the HGDP dataset is used to learn about the ancestry of the Catoctin individuals, an aim that falls within the intended use case. However, a number of criticisms have emerged since the release of the HGDP dataset regarding sampling procedures and informed consent, as the majority of samples included in the dataset came from existing collections that were not originally sampled with the intent of creating a public database of global human genetic variation (e.g. *92*, *93*).

- **Simons Genome Diversity Project**

  We considered individuals whose genetic data was included in the Simons Genome Diversity Project dataset. Only genetic data from individuals who provided

This is the author's version of the work. It is posted here by permission of the AAAS for personal use, not for redistribution. The definitive version was published in Science on August 4th, 2023, DOI: 10.1126/science.ade4995.

informed consent that was consistent with fully public data release on the EBI European Nucleotide Archive was considered. Data uploaded to the European Nucleotide Archive may not be subject to any usage restrictions and must remain permanently accessible as part of the scientific record. For more information about the Simons Genome Diversity Project and the European Nucleotide Archive policies, see: Mallick et al, 2016 and https://www.ebi.ac.uk/ena/browser/about/policies

- **23andMe datasets:**
  - **23andMe Research participants**

This study (like all 23andMe Research studies) only considered the data of 23andMe customers who had actively provided consent to participate in 23andMe Research at the time of study onset (July 28th, 2020). More than 80% of 23andMe customers have provided informed consent to participate in research via a protocol that was approved by an external AAHRPP-accredited IRB, Ethical & Independent Review Services (E&I Review), now known as Salus IRB. Consenting participants provide broad consent to allow 23andMe researchers to study and publish on a wide variety of research topics, including, but not limited to, "The history of peoples across the world, including how they migrated and intermixed in the past." In order to protect participant privacy, 23andMe researchers do not have access to identifying information, such as participants' names and contact information. Additionally, all research results are reported as combined summaries that do not allow identification of any particular individual. Participants are informed that they are unlikely to directly benefit from participation and that they should not expect to

This is the author's version of the work. It is posted here by permission of the AAAS for personal use, not for redistribution. The definitive version was published in Science on August 4th, 2023, DOI: 10.1126/science.ade4995.

receive individual results that are learned through 23andMe Research discoveries. However, they or people with shared ancestry may indirectly benefit if these 23andMe Research discoveries go on to be incorporated into the 23andMe product in the future. 23andMe Research participants have the option to change their consent choice at any time. For more information about the 23andMe Research consent process, see: https://www.23andme.com/about/consent/

- **23andMe's African American Sequencing Project imputation panel:** The 23andMe African American Sequencing Project imputation panel reported in O'Connell et al (*94*) is composed of 23andMe Research participants who provided additional informed consent to allow their individual level genetic data to be uploaded to the database of Genotypes and Phenotypes (dbGap), where it may be accessed by qualified researchers conducting human genetic variation research. The study design and consent were approved by Ethical & Independent Review Services (E&I Review), now known as Salus IRB. For more information about the panel, see O'Connell et al (*94*).
- **23andMe Population Collaboration Datasets:** The following datasets were generated as part of collaborations between 23andMe and academic institutions with the purpose of increasing global representation of genetic databases:
    - 299 individuals from Angola (*22*, *95*). Sampling was conducted and consented for following a research protocol that was approved by ethics committees at the University 11th of November (Universidade 11 de

This is the author's version of the work. It is posted here by permission of the AAAS for personal use, not for redistribution. The definitive version was published in Science on August 4th, 2023, DOI: 10.1126/science.ade4995.

Novembro), Cabinda, Angola (REf: GD-FM/UoN/2016) and the University of Leicester ethics committee (REf: 11334-sdsb1-genetics).

- 605 individuals from The Democratic Republic of the Congo (*85*). Sampling was conducted and consented for following a protocol that was approved by the Harvard Internal Review Board (IRB00000109; Protocol 24087).
- 225 individuals from Sierra Leone (*86*). Sampling was conducted and consented for following a protocol that was approved by IRBs obtained by Boston University and the University of South Carolina.
- 95 Khoe-San speaking people (*87*). Sampling was conducted and consented for following a protocol that was approved by an IRB obtained from Stanford University.

The 23andMe Population Collaboration Program provided support to 23andMe collaborators to genotype research participants from populations that are understudied in terms of genetics. The exact research protocols and consent procedures used in each study was developed in collaboration between 23andMe and the external collaborators who were leading the study. In all cases, informed consent was received that permitted researchers to collect a saliva sample and for 23andMe to store, access and analyze the sample and resulting data. Additionally, collaborating researchers were responsible for gathering demographic information, including participant age, sex, birthplace, ethnic affiliations and languages to be shared with 23andMe. For more information about the 23andMe Population

This is the author's version of the work. It is posted here by permission of the AAAS for personal use, not for redistribution. The definitive version was published in Science on August 4th, 2023, DOI: 10.1126/science.ade4995.

Collaborations Program, see: https://blog.23andme.com/articles/23andmes-population-collaboration-program-supports-research-in-understudied-groups

S1.4 Strategies to Ensure 23andMe Participant Anonymity

23andMe Research participants provided informed consent for their data to be used in 23andMe Research studies with the understanding that 23andMe would not release individual level data to outside collaborators or include it in publications without their explicit and separate consent. Instead, under the 23andMe Research Consent Document (https://www.23andme.com/about/consent/), 23andMe researchers may only share research participant data in a combined format that does not identify any particular individual. We applied several anonymization strategies throughout the study that were created in order to fulfill this requirement, while also enabling us to share fine grained results. Here, we outline the strategies used for each analysis involving 23andMe Research participants:

- **Default approach: k-anonymity (k ≥ 5):**

  Unless otherwise noted, all results generated using the 23andMe dataset were required to meet the requirement of k-anonymity, where k ≥ 5 (*96*). This means that any reported counts that were less than 5 were excluded or masked (i.e. reported as "≤ 5"). This anonymization strategy greatly reduces the chance that individual-level data could be discerned from the reported results.

  Applies to results reported in: Table 1, Figs 3b, 4b, 5b, 6 and associated Supplementary Tables

This is the author's version of the work. It is posted here by permission of the AAAS for personal use, not for redistribution. The definitive version was published in Science on August 4th, 2023, DOI: 10.1126/science.ade4995.

- **Rounding of IBD segment lengths and total IBD:**

  While individual-level results about genetic connections to the Catoctin individuals were not returned to research participants as part of this study, we wanted to ensure that if these results were to be returned in the future, that this information would not make it possible to determine whether specific research participants were included in the study. While it would be difficult to discern whether or not a particular participant was included in the dataset when considering values like average IBD sharing or the proportion of participants in a particular subset, the maximum IBD segment length and maximum total IBD could be more easily linked to a particular research participant if that participant's results were known. We rounded the maximum IBD segment length and total IBD sharing using the following approach to unlink individual results from the values provided in these tables: Values >100 cM were rounded to the nearest ten, values between 30–100 cM were rounded to the nearest five, values between 10–30 cM were rounded to the nearest integer, and values <10 cM were rounded to one decimal place. ***Applies to results reported in:*** Table 1, Figs 3a, 4a, 5a and associated Supplementary Tables

- **Downsampling to 80% and only reporting coordinates with at least 25 associated participants:**

  Many of the geographic signals that we identify in this study would not have been possible to discern using a strategy where counts ≤5 were masked for k-anonymity, particularly at geographic coordinates where there were only a small number of associated individuals. We therefore adopted the following strategy in order to report meaningful results, involving counts ≤5 that were associated with specific geographic coordinates.

This is the author's version of the work. It is posted here by permission of the AAAS for personal use, not for redistribution. The definitive version was published in Science on August 4th, 2023, DOI: 10.1126/science.ade4995.

First, we downsampled the entire dataset to 80% of its original size, meaning that 1 in 5 research participants were excluded from the analysis. Next, we only reported results from geographic coordinates where there were at least 25 associated participants (after downsampling). This means that on average, there were at least 5 individuals from each coordinate that were not included in the analysis. Following this anonymization strategy, it would not be possible to determine whether a particular research participant was included in the analysis, even if their birth or grandparental birth location and the exact amount of IBD they shared with a Catoctin individual were known.

Applies to results reported in: Figs 3a, 4a & 5a and associated Supplementary Figs and Tables

- **Pedigree Figures:**

To ensure the anonymity of research participants in the display of pedigree structures, we did not show pedigrees containing relationships among present-day research participants. We only displayed the relationships among historical individuals along with summary information depicting the connections between historical and modern pedigrees. This ensures that pedigrees with unusual structures, which could be identified as belonging to one or few present-day families, were not depicted. In cases where five or fewer research participants shared a unique connection to the historical pedigree, we masked the exact count and instead reported "≤5".

Applies to results reported in: Fig. 6 and associated Supplementary Figs and Tables

This is the author's version of the work. It is posted here by permission of the AAAS for personal use, not for redistribution. The definitive version was published in Science on August 4th, 2023, DOI: 10.1126/science.ade4995.

S1.5 Terminology Choices

Discussions involving topics such as race, ancestry and gender require careful consideration of terminology. In what follows, we outline choices that we made regarding the terminology that is used throughout the manuscript. In some cases, we used terms that are imprecise in order to maintain the ease of readability of the manuscript, so we have provided a detailed explanation of how these terms should be interpreted.

- **Ancestry, Race and Ethnicity**

  Although they are often incorrectly used interchangeably, the terms ancestry, race and ethnicity have distinct definitions in the fields of anthropology and genetics that researchers must be careful not to conflate (*97*). In genetics, the term "ancestry" refers to one's biological ancestors and their genetic connections to groups of people or places in the past. In contrast, the terms "race" and "ethnicity" are not biologically defined categories. Race is a socially constructed category that is often associated with shared physical characteristics. Members of the same race often share social experiences, while the term ethnicity refers to self-identified cultural groups. Each of these terms and how they are used (correctly and incorrectly) by researchers in the fields of anthropology and genetics are discussed in detail in Birney et al (*97*), Wagner et al (*98*) and Mathieson and Scally (*99*).

  The term "African American" is often associated with descendants of enslaved individuals of Sub-Saharan African ancestry in the US, while the term "Black" is applied more broadly, also encompassing individuals whose ancestors (or who themselves) moved to the US more recently and individuals who live outside the US (*100*). As this study is specifically focused on individuals buried in the Catoctin Furnace African American Cemetery, the majority of

This is the author's version of the work. It is posted here by permission of the AAAS for personal use, not for redistribution. The definitive version was published in Science on August 4th, 2023, DOI: 10.1126/science.ade4995.

whom were enslaved, we primarily use the term "African American" throughout the manuscript. However, we also use the term "Black" where it is more appropriate, such as to match how race-based information was collected by outside sources (such as the US census) or when paired with the term "white" (to maintain linguistic continuity). We follow the Associated Press style guidelines, which recommend that the term "Black" be capitalized while "white" not be when referring to race (*101*). When discussing genetic ancestry, we refer to continental-level ancestry categories, either defined in relation to one or more representative populations sampled from each region (as in the qpAdm, ADMIXTURE and PCA analyses) or to assignments made by the 23andMe Ancestry Composition algorithm (*42*), whose categories are also defined using a panel of representative individuals with deep ancestral ties to known geographic regions. We do not use genetic ancestry to assign the Catoctin individuals or research participants to specific racial or ethnic groups (i.e. African American) and instead refer to their continental-level ancestry assignments (i.e. participants with at least 5% Sub-Saharan African ancestry). In cases where we do refer to research participants as African American, we are referring specifically to their self-reported ethnicity.

Race and ethnicity are not defined by genetic ancestry, and continental-level ancestry categories can obfuscate the differences (and similarities) that exist between different groups of people (e.g. *102*, *103*). However, there are correlations between people who identify as African American and genetic ancestry (*39*), and we believe that it is important to highlight the historical associations between these distinct categories in cases where genetic signatures may reflect or shed new light on historical events and/or patterns that were known to have impacted African American individuals. For instance, we observe a

higher number of European-associated Y-haplogroups among the Catoctin individuals relative to the number of European-associated mt-haplogroups. This is a likely a genetic signature of historically well-documented patterns in which white men raped enslaved Black women (*38*). These acts have left a genetic signature that is detectable in the DNA of many historic and present-day individuals in the US with African ancestry.

- **Genetic sex and gender**

We determine the genetic, or chromosomal, sex of the Catoctin individuals based on X to Y-chromosome ratios. Throughout the manuscript, we refer to individuals who are determined to have XY sex chromosomes as male and those who have XX sex chromosomes as female. We chose to use the singular, gender-neutral pronoun 'they' when referring to individuals, in acknowledgement that although genetic sex and gender are often correlated, gender is not defined by genetic sex (*104*).

- **Genetic relatedness and kinship**

We use kinship-based terminology to describe the genetic relationships detected between the Catoctin individuals, referring to groups of individuals with close genetic relationships as genetic families and using terms such as mother and child/son/daughter to refer to specific genetic relationships that are shared between individuals in each family. We acknowledge that genetic relatedness does not necessarily dictate how kin relationships were defined between the Catoctin individuals (*105*). Therefore, in all cases, any terms that we use to define relatedness refer specifically to genetic relatedness (i.e., "mother" may be read as "genetic mother" or "biological mother"), rather than to kinship.

This is the author's version of the work. It is posted here by permission of the AAAS for personal use, not for redistribution. The definitive version was published in Science on August 4th, 2023, DOI: 10.1126/science.ade4995.

Supplementary Text S2: The Return of Names

An overlook located close to the Catoctin Furnace cemetery includes three interpretive panels. The panels provide information about the erasure and rediscovery of the cemetery, and a list of 271 given names found in land records, probate inventories, church records, diaries, and freedom-seeker ads, which we list below. Notice the number of names listed simply as "unknown" and the absence of any last names. As we read their names, which are listed on the following pages, these people are remembered.

"a person is not forgotten until his or her name is forgotten"

In reading these names to you, you ensure enslaved workers at Catoctin Furnace are not forgotten:

This is the author's version of the work. It is posted here by permission of the AAAS for personal use, not for redistribution. The definitive version was published in Science on August 4th, 2023, DOI: 10.1126/science.ade4995.

Jack
Jaines
Milly
Phil
Ben
Nelly
Sam
John
Thomas
John
Clemens
Jenny
Polly
Rachel
Samuel Hercules
Caty
Clemens
Maria
Petty
Sammy
Susanna
Ally
Anne
Ben
Betty
Betty
Big Dick
Bill
Bill
Bill
Cate
Charlot
Christina
Cloay
Elsey
Estor
Farm Jacob
Frederick
George
Harriet
Harry
Harvey
Henry
Hetty
Humbert
Jack
Jacob
Jane
Jane
Jane
Janey
Joe
Larkin
Len
Lewis
Liddy
Little Bill
Lock
Lorena
Lusinda
Magdeline
Maria
Mary
Mary
Mattie
Mill
Milly
Mingo
Nanny
Ned
Nell
Old Hanna
Old Jack
Old Will
Parris
Priss
Resey
Romeo
Rose
Sal
Sam
Sam
Samuel
Sue
Sue
Toby
Wally
Will
Yellow Girl
Hanson
Big Bill
Daniel
Isaac
John
William
William of Elizabeth
Jacob
Moses
Nicholas
Richard
Mary
Daniel
Anne
Anthony
Richard
James
Patsy
Nace
Rachel
Ann
Bill
Bob
Charity
Christiana
David
Eliza
George
George
Harrison
Harry
Henry
Isaac
Leonard
Lucky
Lucy
Peter
Sarah
Stacy
Wally
Sarah
Leonard
Jeremiah
Henry
Peter
Nathaniel
Ruth
Polly
Richard
William
Priscilla
Phebe
George
Jessee
Thomas
James
Richard
Richard Jr
Thomas
Henry
Peter

This is the author's version of the work. It is posted here by permission of the AAAS for personal use, not for redistribution. The definitive version was published in Science on August 4th, 2023, DOI: 10.1126/science.ade4995.

Celeste
Benjamin
Unknown
Unknown
Unknown
Unknown
Unknown
Unknown
Unknown
Unknown
Unknown
Unknown
Unknown
Unknown
Unknown
Unknown
Unknown
Unknown
Unknown
Unknown
Unknown
Charles
Elias
Rebecca
Isabella
John
Elizabeth
James
Joseph
Maria
Nathan
William
George
Milly
William
Eliza
Joseph
Ann
Elias
Elizabeth
Hesekiah
Hesekiah
James
John
John
John
Mary
Mary
Mary
Nathaniel
Otho
Anna
Ann
Archibald
Bisi
Daniel
Elias
Lulian [Julian]
Mary
Mary
Mary
Merideth
Savilla [Sasilla]
Susan
Thomas
William
Carlline [Caroline]
Ellen
James
John
Mary
Sarah
William
Zachariah
John
Annenise
Emiley
Georgeana
James
Daniel
Martin
Sylvester
Caroline
Henry
Jane
Joseph
Lucy
Martha
Philip
Sarah
Henry
Elizabeth
Hezakiah
John
Nathan
Otho
Sarah
Susan
Ann
Isaac
John
Julia
Julia
Nick
Peter
Sarah
Simon
Andrew
Julia
Lucy
Mary
Peter
Sarah
Sylvester
Andrew
Eliza
John
John
Joseph
Mary
William

Total 271

This is the author's version of the work. It is posted here by permission of the AAAS for personal use, not for redistribution. The definitive version was published in Science on August 4th, 2023, DOI: 10.1126/science.ade4995.

Supplementary Text S3. Testing the application of IBD detection methods to imputed low coverage ancient DNA using simulated data

S3.1 Data preparation

We selected 32 high coverage ancient genomes from varying time periods and regions across the globe in order to test the performance of TPBWT(*41*) when applied to imputed genotypes from low coverage, captured ancient DNA data. The imputation was performed using the tool GLIMPSE (*40*) with an approach that has been optimized for low-coverage, capture-based ancient DNA data. All 32 individuals included in this test dataset are of at least 20x coverage, except for two individuals (I6714.SG and I3388.SG) that were included as they are first degree relatives of two other high coverage individuals (Table S3.1).

Table S3.1. Ancient Individual Background

Individual ID	Genetic Sex	Country where individual was excavated	Average age (years before present)	Coverage (average autosomal)	Original Publication	Publication of High Coverage Shotgun Data	Notes
HSJ-A-1_38.SG	M	Iceland	1015	34.68	(*106*)	(*106*)	
I2861.SG	F	Great Britain	2853	20.54	(*107*)	(*108*)	
I1053.SG	M	Russia	3793	20.22	(*84*)	(*108*)	
I5835.SG	M	Germany	4200	23.75	(*107*)	(*108*)	
I2514.SG	M	Iran	4341	21.30	(*84*)	(*108*)	
I1633.SG	F	Armenia	4465	20.89	(*109*)	(*108*)	
I5950.SG	M	Ethiopia	4472	22.68	(*110*)	(*108*)	
I6714.SG	M	Russia	4495	17.95	(*84*)	(*108*)	Afanasievo Family – Son B
I3388.SG	F	Russia	4600	8.95	(*84*)	(*108*)	Afanasievo Family – Mother
I3949.SG	M	Russia	4618	22.78	(*84*)	(*108*)	Afanasievo Family – Son A
I3950.SG	M	Russia	4707	23.33	(*84*)	(*108*)	Afanasievo Family – Father
Yamnaya.SG	M	Kazakhstan	4903	27.32	(*111*)	(*111*)	
I5279.SG	M	Russia	4905	24.58	(*84*)	(*108*)	
I2935.SG	M	Great Britain	5123	23.95	(*107*)	(*108*)	
I2978.SG	M	Great Britain	5129	30.90	(*107*)	(*108*)	
I2520.SG	M	Bulgaria	5132	22.41	(*76*)	(*108*)	
I2980.SG	F	Great Britain	5181	26.20	(*107*)	(*108*)	
I3133.SG	M	Great Britain	5455	26.27	(*107*)	(*108*)	
I1631.SG	F	Armenia	6100	29.62	(*109*)	(*108*)	

This is the author's version of the work. It is posted here by permission of the AAAS for personal use, not for redistribution. The definitive version was published in Science on August 4th, 2023, DOI: 10.1126/science.ade4995.

I5077.SG	M	Croatia	7026	24.24	(*76*)	(*108*)	
I1496.SG	M	Hungary	7052	26.07	(*112*)	(*108*)	
I4438.SG	M	Latvia	7291	20.63	(*76*)	(*108*)	
I4878.SG	M	Serbia	7803	22.62	(*76*)	(*108*)	
I4873.SG	F	Serbia	7872	21.95	(*76*)	(*108*)	
I4596.SG	M	Latvia	7976	20.27	(*76*)	(*108*)	
I4914.SG	M	Serbia	8123	22.19	(*76*)	(*108*)	
I1960.SG	F	Russia	8166	20.40	(*84*)	(*108*)	
I1583.SG	M	Turkey	8281	21.50	(*23*)	(*113*)	
I4877.SG	F	Serbia	8505	23.33	(*76*)	(*108*)	
SF12.SG	F	Sweden	8895	65.47	(*114*)	(*114*)	
I5236.SG	M	Serbia	10008	23.10	(*76*)	(*108*)	
I5235.SG	M	Serbia	10835	20.12	(*76*)	(*108*)	

We used samtools view (v1.10.2) to subsample reads and create bam files with coverages of 0.01x, 0.05x, 0.1x, 0.2x, 0.5x, 1x, 2x, 5x, max (the max case was only generated for shotgun-style simulations) (Fig. S3.1). For shotgun (SG) and 1.2 million SNP capture (1240k) simulations we only keep reads that overlap with 1000 Genomes project phase 3 (*68*) and the 1.2 million sites targeted during enrichment capture (*23–25*), respectively. These bam files were used as input to the imputation pipeline to estimate the Genotype Posterior (GP=[GP0, GP1, GP2]) for each genotype. The genotype with highest GP value was used as the imputed genotype.

Following imputation, the data were filtered to remove genotype calls with an estimated maximum genotype posterior below a minimum threshold (henceforth referred to as the "max(GP) threshold") of 0.85, 0.90, 0.95 or 0.99, resulting in 2,176 simulated test cases. We also considered the non-imputed versions of the original 32 individuals in our analyses, using the most likely genotype calls generated by bcftools mpileup (v1.10.12) prior to imputation. In cases where no genotype could be predicted or two genotypes were equally likely, the genotype was considered missing. All data were then rephased with EAGLE (*73*) using a panel of 706,995 23andMe

This is the author's version of the work. It is posted here by permission of the AAAS for personal use, not for redistribution. The definitive version was published in Science on August 4th, 2023, DOI: 10.1126/science.ade4995.

research participants at 541,948 SNPs, with default settings and optional parameter --allowRefAltSwap. Missing genotypes were not re-imputed.

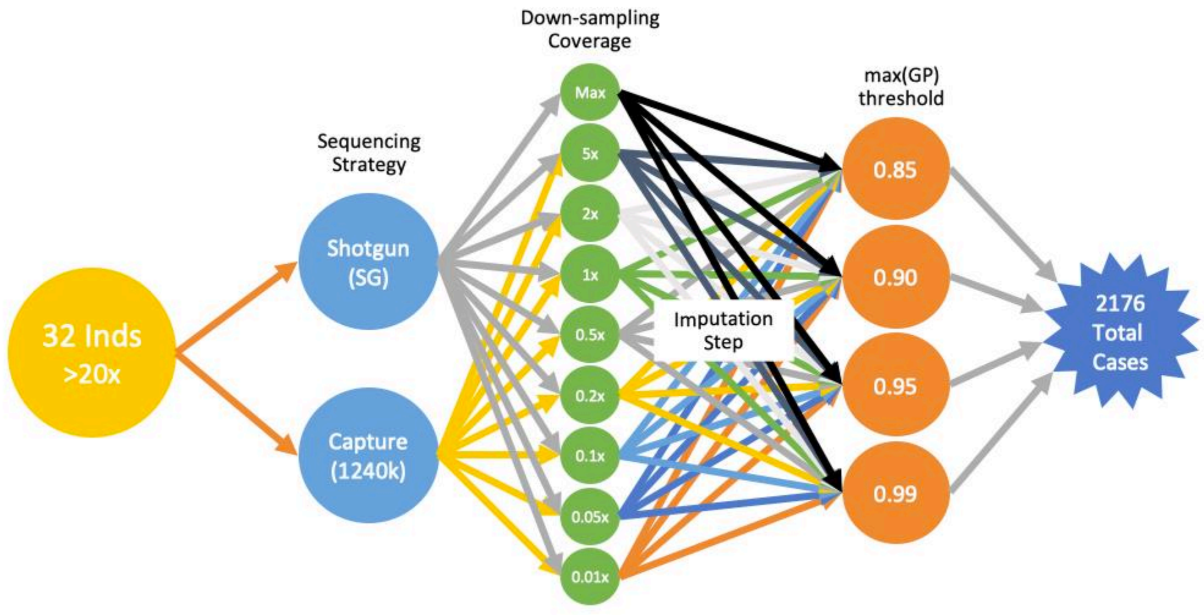

Fig. S3.1. Schematic of simulated data processing strategies. The schematic shows the various conditions that were generated at each stage of processing. Starting with 32 high coverage ancient individuals, the data modified to mirror shotgun (SG) and capture (1240k) sequencing strategies. Then the data were downsampled to coverages between 0.1x-5x (also retaining the max coverage case for shotgun data). Next, data was filtered to retain sites with a max(GP) threshold between 0.85-0.99, resulting in a total of 2,176 cases.

S3.2 Impact of data quality reduction on total IBD sharing

While IBD-based tools have previously been applied to ancient DNA (*115–118*), these analyses have been restricted to shotgun sequence data. In order to ensure that the process of imputation on low-coverage, captured sequence data did not introduce substantial biases, we explored the

This is the author's version of the work. It is posted here by permission of the AAAS for personal use, not for redistribution. The definitive version was published in Science on August 4th, 2023, DOI: 10.1126/science.ade4995.

impact of data generation strategy (i.e,. capture versus shotgun) and down-sampling coverages on IBD segment detection. For each case, we used TPBWT (*41*) to detect IBD shared with a panel of ~470,863 23andMe research participants who indicated having 4 grandparents that were born in the same region, including ~200,000 research participants whose grandparents were born in the same US state and an additional ~270,000 research participants whose grandparents were born in a single country. We emphasize that imputation is an essential step in our process, not only because it allows inference of diploid genotypes, but also because only 22.3% (146,701/656,786) of the version 5 panel of SNPs genotyped by 23andMe were targeted in the 1240k capture process.

We detected 229,492,064 IBD segments shared between all the downsampled cases and the 23andMe panel, ranging in size from 3cm (the minimum segment length output by TPBWT) to 32 cM (Fig. S3.2a; Table S3.2). The majority of IBD segments (71.8%) were between 3-4cm in length, while fewer than 0.5% of segments exceeded 8cm. The relative abundance of short IBD segments is expected, as shorter IBD segments reflect more distant relatedness (*119*, *120*).

Because imputation can potentially introduce false positive IBD segments, particularly for the lowest coverage cases, we determined the rate of false positive IBD detected across all scenarios. We assumed that IBD segments detected between 23andMe research participants and the highest coverage cases (i.e. DP max) represented true IBD segments, while segments only observed in lower coverage cases represented false positive segments introduced by imputation. We defined false positive rate (FPR) as the number of false positive IBD segments detected in a lower coverage case divided by the total number of segments detected in that case. In our calculations, segments

This is the author's version of the work. It is posted here by permission of the AAAS for personal use, not for redistribution. The definitive version was published in Science on August 4th, 2023, DOI: 10.1126/science.ade4995.

that overlapped at any position were considered to be the same segment. In Fig. S3.2b (Table S3.3) we present the weighted average of the FPR for each case, where the relative weight of each ancient individual is determined based on the number of IBD segments detected in the highest coverage (DP max) condition.

We found that FPR increases as coverage decreases with a higher rate of increase in capture data. The difference in performance of capture versus shotgun data is unsurprising as coverage measurements are not equivalent between the two data types—for shotgun sequenced data, coverage refers to the average number of reads aligning to every site in the human genome, while only the targeted sites are considered in measurements of coverage for capture data. The large amount of data from non-targeted SNPs available from shotgun data greatly increases the precision with which we can make genotype inferences at all SNP positions including targeted ones. We also found that the FPR was generally similar across the various max(GP) thresholds, with slightly higher FPRs typically associated with max(GP) threshold of 0.99, particularly in lowest coverage cases. This likely reflects the relative increase in missing data associated with using a more stringent filtering threshold. In order to strike a balance between the number of reliably imputed genotype calls and the amount of missing data, we chose to apply a max(GP) threshold of 0.95 going forward. However, we note a minimal impact of varying max(GP) threshold across subsequent analyses. We also focus on the capture-style data going forward in order to mirror the data available for study in the main text, again noting that we observe similar trends in the shotgun style data.

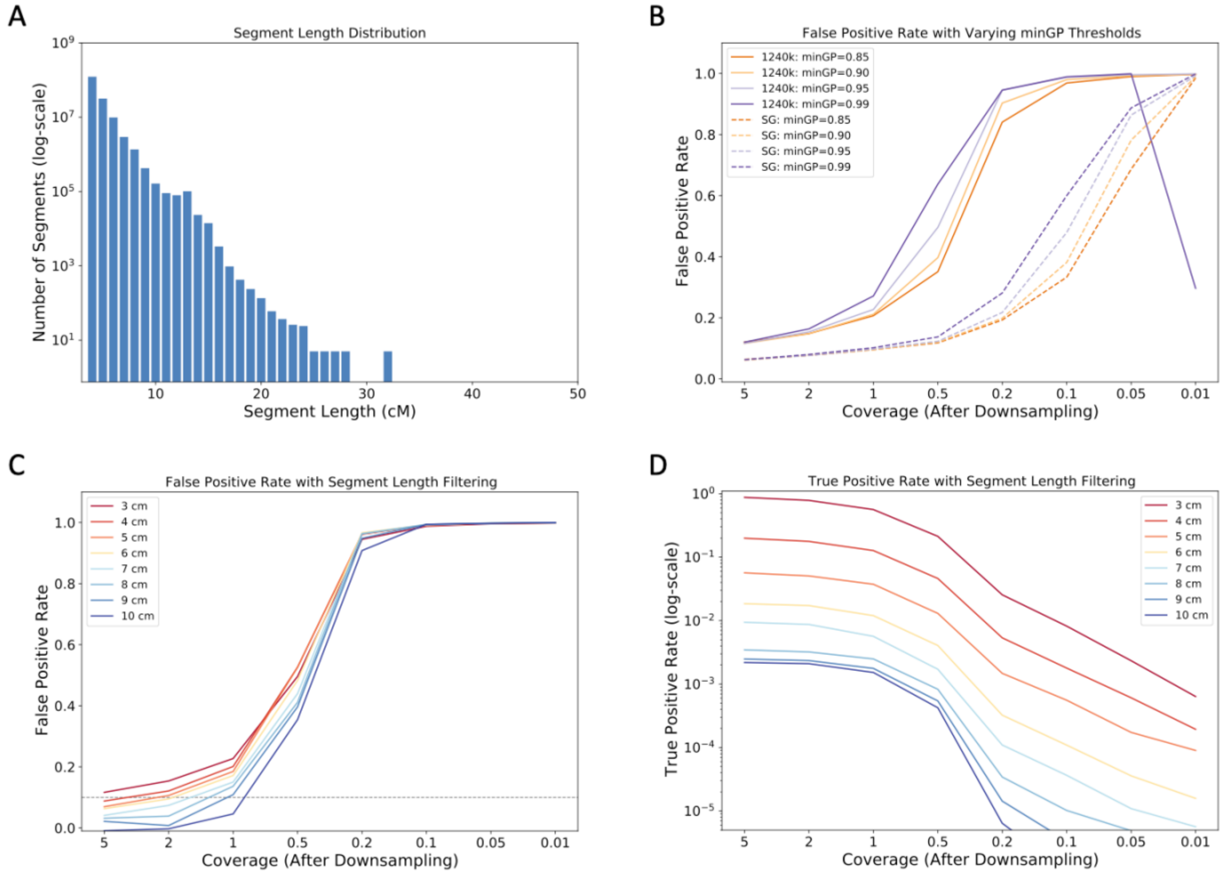

Fig. S3.2 IBD Segment Authenticity [A] Histogram of IBD segment lengths shared with 23andMe research participant panel across all simulated cases. x-axis labels indicate the upper bound of each size bin (i.e. 4cM indicates the 3-4cM bin). Segment counts are shown on the y-axis using a log scale. [B] FPR for shotgun (dotted line) and 1240k capture (solid line) style sequence data, with varying max(GP) thresholds (as indicated by line color). FPR was calculated as the proportion of IBD segments in a specified case that do not occur in the corresponding highest coverage (DP max) case. We present the weighted average of the FPR for each case, where the relative weight of each ancient individual is determined based on the number of IBD segments detected in the highest coverage (DP max) condition. [C] Weighted average FPR for the max(GP) threshold = 0.95 for 1240k capture style data after filtering using a variable minimum segment length threshold between 3-10cM (as indicated by line color). [D] Weighted average TPR for the

This is the author's version of the work. It is posted here by permission of the AAAS for personal use, not for redistribution. The definitive version was published in Science on August 4th, 2023, DOI: 10.1126/science.ade4995.

max(GP) threshold = 0.95 for 1240k capture style data after filtering using a variable minimum segment length threshold between 3-10cM (as indicated by line color). The TPR was calculated as the proportion of IBD segments that are detected in the max coverage case that are also detected in the lower coverage case. TPR is shown on the y-axis using a log scale.

To minimize the FPR while maximizing the true positive rate (TPR) when studying capture-style sequenced ancient DNA we explored the impact of filtering IBD segments based on length, with the hypothesis that shorter reads are more likely to be false positives. We find that the FPR decreases as more stringent minimum IBD segment length filters are applied (Fig. S3.2c, Table S3.3). At 5x coverage, a minimum segment length threshold of 4cm is sufficient to reduce the weighted average FPR to 9.8%. However, as coverage decreases, longer minimum segment length thresholds are required to reach the 10% FPR threshold.

While length thresholding reduces FPR, it also dramatically reduces the number of true IBD segments detected. Therefore, we considered the impact of length filtering on the true positive rate (TPR)—the proportion of IBD segments that are detected in the max coverage case that are also detected in the lower coverage case. Mirroring trends observed with FPR, when using the standard minimum segment length threshold of 3cM TPR is maximized in the highest coverage cases and dramatically declines as coverage decreases (Fig. S3.2d, Table S3.3). Applying a more stringent minimum length threshold dramatically reduces the TPR, even in the highest coverage cases, indicating that many of the shorter IBD segments removed by length filtering are real.

This is the author's version of the work. It is posted here by permission of the AAAS for personal use, not for redistribution. The definitive version was published in Science on August 4th, 2023, DOI: 10.1126/science.ade4995.

In order to balance the reduction in false positive IBD with the loss of true IBD associated with length filtering, we chose to apply a sliding scale minimum segment length threshold to our TPBWT results, selecting the minimum segment length threshold at each coverage that is closest to a 10% FPR. For empirical analyses we applied the following thresholds:

Coverage (x)	Minimum IBD Segment Length
>5	4 cM
[2,5)	6 cM
[1,2)	9 cM
<1	10 cM

We note that at 0.5x coverage and below, none of the thresholds that we tested substantially reduced the FPR. We therefore chose to apply a minimum segment length threshold of 10 cM for the purposes of testing.

S3.3 Exploring broad geographic signals of IBD sharing

In order to assess whether the IBD segments detected between the ancient individuals and the panel of 23andMe participants reflect known historical patterns, we considered the geographic distribution of IBD sharing. Since each country in our dataset has a different number of associated participants who report ancestry from the region, we considered the average total IBD shared with 23andMe participants from each country in each case. To protect individual research participant privacy, we only report results for countries with a minimum of 5 associated 23andMe research

This is the author's version of the work. It is posted here by permission of the AAAS for personal use, not for redistribution. The definitive version was published in Science on August 4th, 2023, DOI: 10.1126/science.ade4995.

participants. Here we highlight the results of 3 ancient individuals who serve as representative examples of general trends that we observed in this dataset.

First, we considered individual HSJ-A-1_38.SG. This individual was excavated in Iceland and dates to approximately 1015 years before present (*106*), making them the most recent individual in our test dataset. The highest average IBD is shared between this individual and 23andMe participants with 4 grandparents from Iceland for all cases with at least 0.2x coverage, including the non-imputed case (Fig. S3.3a-e, Table S3.4). Research participants with ancestry from Northwestern Europe, including Scandinavian countries, Great Britain and Ireland also show a high rate of IBD sharing with this ancient Icelander. This pattern of IBD sharing directly matches our historical expectations, as Iceland was colonized by populations with ancestry from these regions. Further, ancient Icelanders have previously been shown to be even more genetically similar to present-day Scandinavian, British and Irish populations than present-day Icelandic people, likely due to the genetic drift that inhabitants of the region have undergone in the millennium since this ancient individual lived (*106*). The most total IBD is observed in the 5x coverage case, even relative to the non-imputed dataset. We believe that the relative increase in IBD detected in the high coverage imputed dataset versus the non-imputed dataset is due to the greater accuracy of genotype calls in this dataset, as the non-imputed dataset did not undergo any filtering based on the likelihood of the genotype calls and likely includes more incorrect genotype calls than the high coverage imputed dataset. Similarly, we observe a relative decrease in total IBD for the imputed cases down-sampled to lower coverages, in part due to the higher minimum segment length thresholds that we apply in the lower coverage cases. However, this decrease in IBD is relatively uniform across the entire 23andMe cohort, therefore the geographic pattern is

This is the author's version of the work. It is posted here by permission of the AAAS for personal use, not for redistribution. The definitive version was published in Science on August 4th, 2023, DOI: 10.1126/science.ade4995.

apparent even in the 0.2x coverage case. In the 0.1x coverage case and below, the geographic pattern is lost, as almost no IBD is detected between any 23andMe participants.

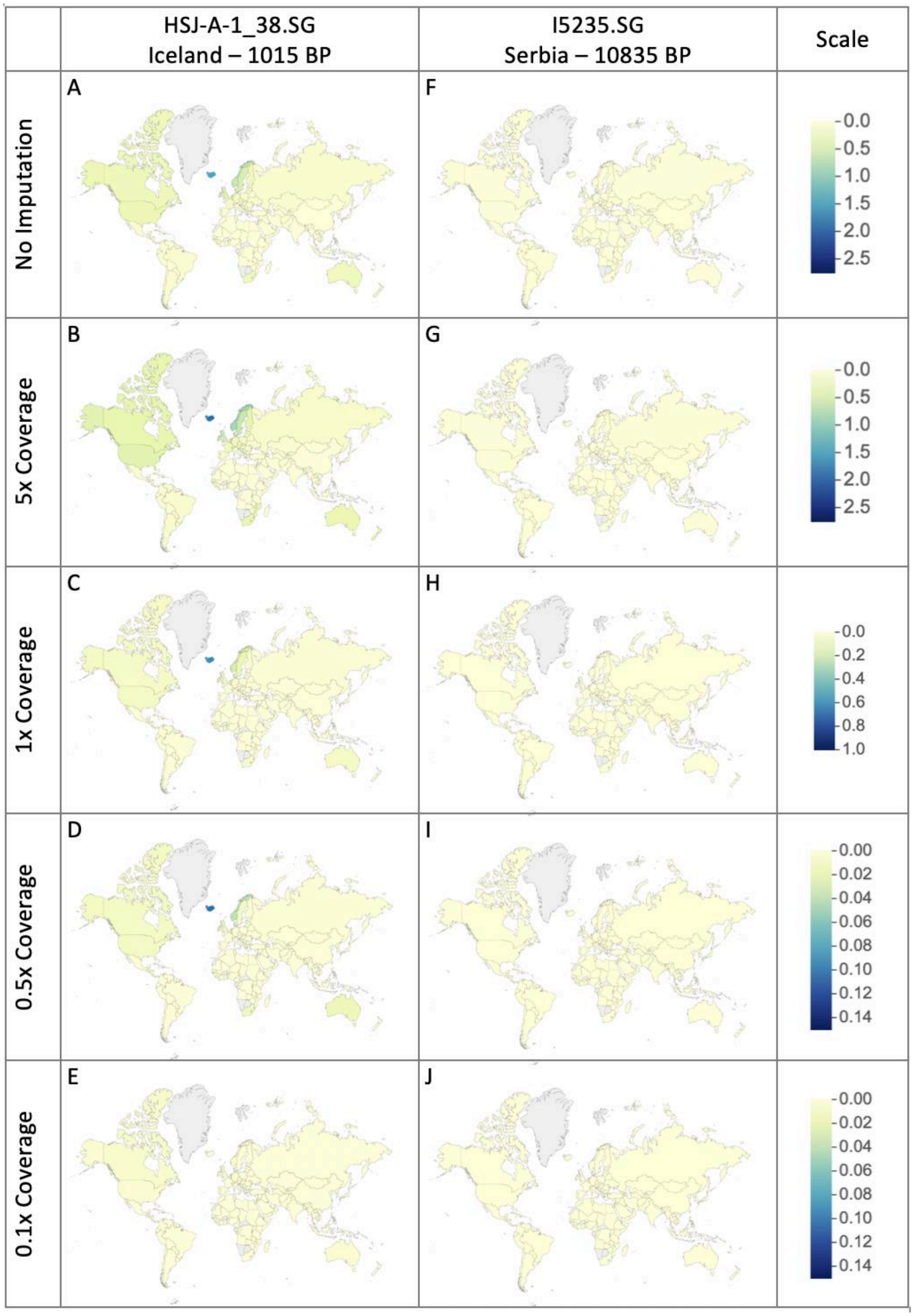

This is the author's version of the work. It is posted here by permission of the AAAS for personal use, not for redistribution. The definitive version was published in Science on August 4th, 2023, DOI: 10.1126/science.ade4995.

Fig. S3.3. Geographic distribution of average IBD sharing. Average IBD shared between an ancient individual and 23andMe research participants with 4GP from each country. Higher average IBD is highlighted in blue, while lower average IBD is shown in yellow. Countries with fewer than 5 associated 23andMe research participants are shown in gray. Average IBD shared with each of three ancient individuals is shown in each column, while varying coverages are shown in each row. In all cases, except for the non-imputed version, results for the 1240k capture style data filtered using a max(GP) threshold of 0.95 is shown, with minimum IBD segment length thresholds applied based on coverage. The color scale used for each coverage varies and is indicated by the scale bar to the right of each row.

In contrast, we detect very little IBD shared between the oldest ancient individual in the test dataset—a 10,835 year old individual from present-day Serbia (*76*)—and the panel of 23andMe research participants for any case (Fig. S3.3 f-j). This failure to detect IBD shared between 23andMe research participants and an ancient individual of this age is expected, as IBD breaks down over time due to recombination. The likelihood of an IBD segment over 4 cM in length being shared between individuals that are separated in time by almost 11 thousand years is nearly negligible (*119*).

S3.4 Detecting relatedness between ancient individuals

Among the 48 high coverage ancient individuals used for testing are 4 individuals that form a genetically related family unit, consisting of a biological father (I3950.SG), mother (I3388.SG) and two sons (I3949.SG and I6714.SG). These individuals were originally described and identified as relatives in Narasimhan et al. (*84*), and the shotgun sequencing data are reported in Wohns et

This is the author's version of the work. It is posted here by permission of the AAAS for personal use, not for redistribution. The definitive version was published in Science on August 4th, 2023, DOI: 10.1126/science.ade4995.

al. (*121*) The inclusion of these related individuals in our test dataset provided the opportunity to determine how well TPBWT can detect IBD in known relatives.

We therefore compared all 32 individuals to one another using TPBWT. In order to understand the impact of imputation of low coverage data, we compared cases with 0.1-5x coverage (with the same coverage used for each individual in the comparison) and included comparisons with the highest coverage shotgun case. At the highest coverage, first-degree relatives in the Afanasievo family share substantially more IBD than any other pair of individuals (Fig. S3.4a, Table S3.5). Further, the total IBD detected between the related Afanasievo family members is highly dependent upon coverage, with the most total IBD detected at 5x coverage. These results indicate that total IBD detected between ancient individuals depends on both the degree of relatedness and coverage, and therefore the degree of closeness of a relationship cannot be directly inferred from the total IBD measured between two individuals without taking the coverage of those individuals into consideration. Further, we observe an increase in total IBD detected between unrelated individuals at lower coverages. By 0.2x coverage it is not possible to distinguish between the known 1st degree relatives and random pairs of unrelated individuals. We therefore caution that total IBD measured between two ancient individuals with less than 1x coverage should be interpreted with extreme caution.

This is the author's version of the work. It is posted here by permission of the AAAS for personal use, not for redistribution. The definitive version was published in Science on August 4th, 2023, DOI: 10.1126/science.ade4995.

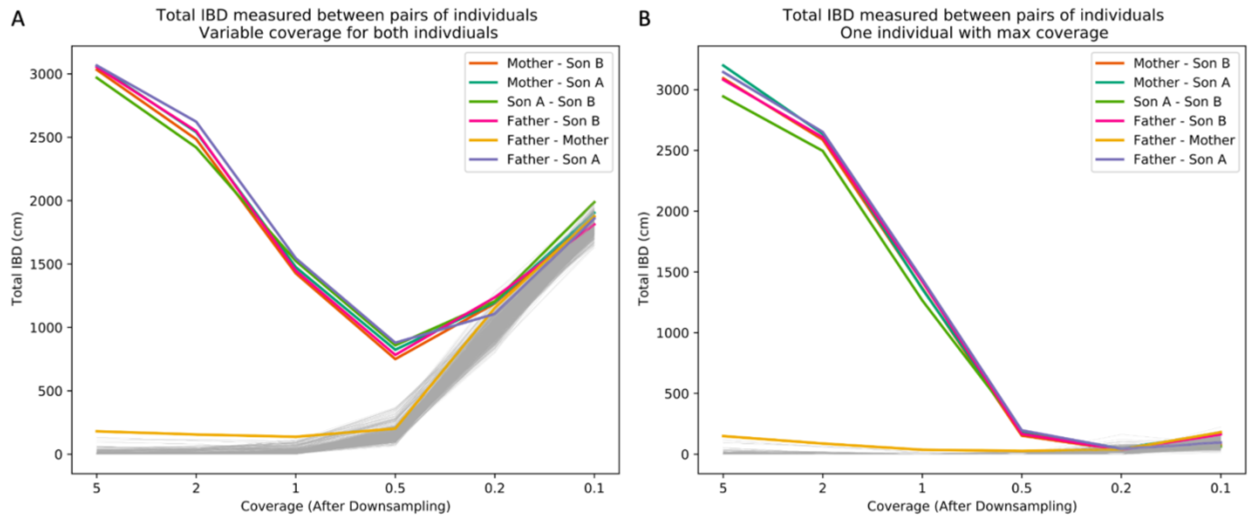

Fig. S3.4. Total IBD detected between ancient individuals. Total IBD measured by TPBWT in pairwise comparisons of all ancient individuals. Comparisons between members of the Afanasievo family are shown in varying colors (see legend) while all other comparisons are shown in gray. We show the change in total IBD detected at varying down-sampling coverages, where either both individuals are down-sampled to the same coverage [A] or a single down-sampled individual compared with the highest coverage (DP max) case.

In comparisons between 23andMe participants and ancient individuals, only the ancient individuals will be impacted by reduced data quality, as the diploid genotypes of 23andMe research participants are called with a high degree of confidence. We therefore decided to repeat our comparison of the ancient individuals while only downsampling a single ancient individual and using the highest coverage version (DP max) of the second individual (Fig. S3.54b, Table S3.6) in order to better mirror this scenario. In this case, we find a more rapid decline in total IBD detected between the first-degree relatives as coverage decreases; however, we do not detect substantial IBD between the unrelated pairs of individuals, even at the lowest coverage threshold. These

results suggest that when substantial IBD is detected between an ancient individual with at least 0.5x coverage and a 23andMe participant, it likely reflects a true genetic relationship. Again, however, it is not possible to directly translate the total amount of IBD shared into a specific degree of relatedness without taking the coverage of the ancient individual into consideration.

Finally, in order to understand how the distribution of IBD segments detected between relatives changes as coverages decreases, we mapped the genetic positions of the IBD segments detected between the two highest coverage members of the Afanasievo family—I3950.SG (Father) and I3949.SG (Son A). In a comparison between the highest coverage cases (DP max), we detect IBD across almost the entire genome (Fig. S3.5, Table S3.7). Additionally, we observed relatively few instances where two IBD segments were detected in the same region (i.e., full IBD), consistent with the distribution of IBD segments expected between a father and son pair. In the few cases where we do detect full IBD, many of these regions are also areas where we detect IBD shared between the father and mother. Thus, these may represent true instances of full IBD sharing between the father and son, as the son may have also inherited an identical segment from the mother. When we reduce the coverage of a single individual (I3949.SG), we again show that as coverage decreases we fail to detect additional true IBD.

This is the author's version of the work. It is posted here by permission of the AAAS for personal use, not for redistribution. The definitive version was published in Science on August 4th, 2023, DOI: 10.1126/science.ade4995.

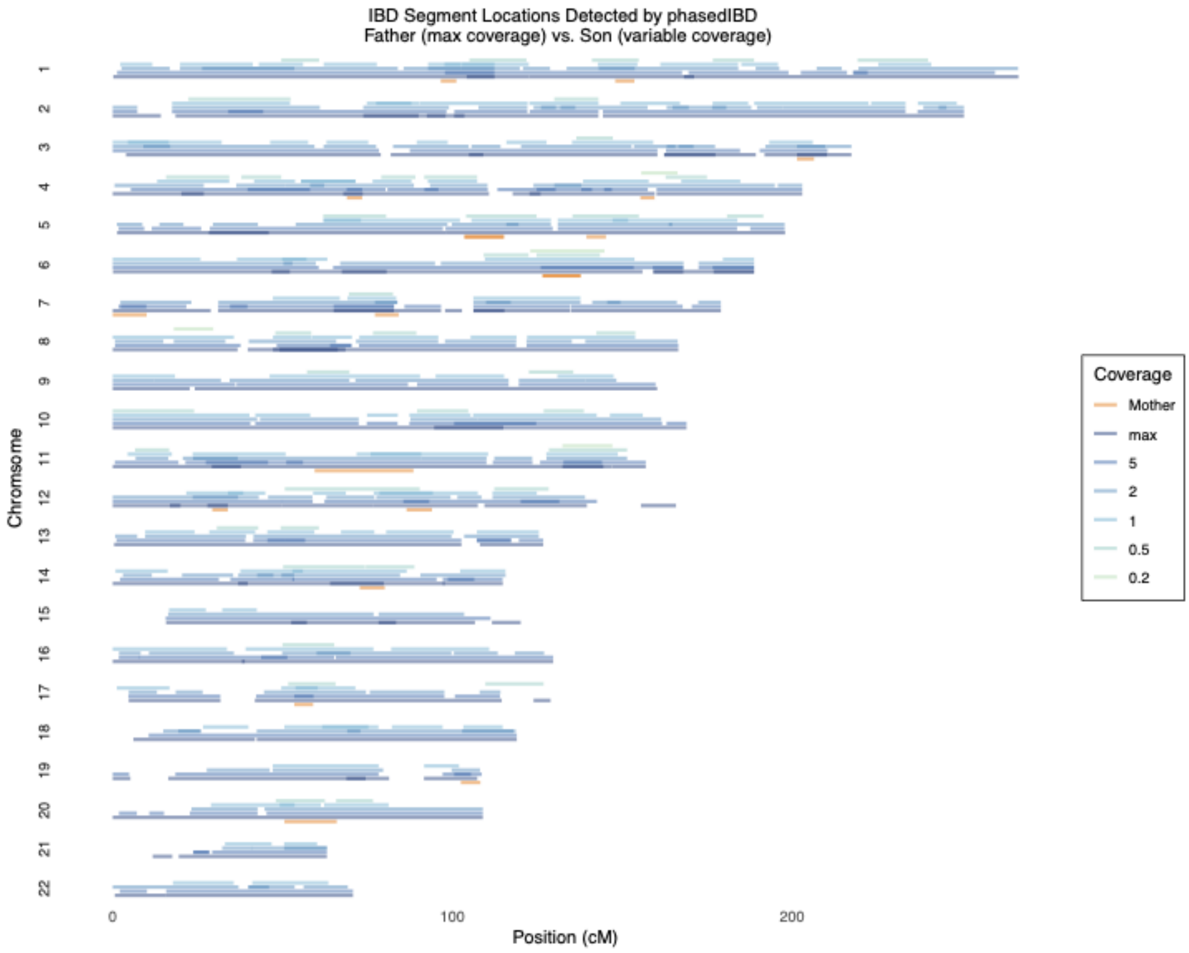

Fig. S3.5. Location of IBD segments detected between Father (I3950.SG) and Son A (I3949.SG) at varying coverages. In all cases, the highest coverage version (DP max) of the father is compared with Son A, downsampled to varying coverages, as specified by segment color. Positions where full IBD is detected (i.e. two overlapping IBD segments are detected in single pair) are indicated by darker colors. Positions where IBD is detected between the mother and father (in the max case for both individuals) are shown in orange.

S3.6 Guidelines for IBD analysis of imputed ancient DNA

Taken together, these results suggest several guidelines for IBD analysis of imputed data which we will follow for all analyses in the main text. First, we require an absolute minimum coverage

This is the author's version of the work. It is posted here by permission of the AAAS for personal use, not for redistribution. The definitive version was published in Science on August 4th, 2023, DOI: 10.1126/science.ade4995.

of 0.5x for ancient individuals sequenced using a 1240k capture-based approach (in cases where shotgun sequenced data is considered, a threshold of 0.05x is acceptable), and will interpret results for individuals with <1x coverage with caution. In order to maximize genotype call quality while minimizing missing data, we filter all imputed data using a max(GP) threshold of 0.95. Finally, in order to reduce false positive IBD, while still capturing as much true IBD as possible, we only consider IBD segments that exceed a minimum length threshold, as defined by the coverage of the individual.

This is the author's version of the work. It is posted here by permission of the AAAS for personal use, not for redistribution. The definitive version was published in Science on August 4th, 2023, DOI: 10.1126/science.ade4995.

Supplementary Text S4: Testing the performance of Ancestry Composition on imputed low coverage ancient DNA using simulated data

23andMe's Ancestry Composition (*42*) algorithm infers local ancestry in genomic windows across each chromosome. Ancestry Composition was designed to predict local ancestry using high coverage genotypic data from present-day individuals. Consequently, there are several factors that might impact the performance of Ancestry Composition when applied to aDNA data, such as biases caused by imputation and the presence of missing data (even at the rate that it is present in an imputed dataset that has been filtered to remove low confidence genotype calls). Therefore, using the test dataset described in Supplementary Text S4, we evaluated Ancestry Composition applied to imputed ancient genomes generated from low-coverage capture-based sequence data.

Since Ancestry Composition was not designed for use with missing data, we also created an unfiltered dataset for which we did not perform any filtering based on the quality of the inferred genotype. We refer to this unfiltered dataset as having a minimum maximum genotype posterior (max(GP) threshold) of 0.00, and compared this dataset to the max(GP) thresholds of 0.85, 0.90, 0.95 and 0.99 datasets described in Supplementary Text S3.

Ancestry Composition predicts local ancestry using present-day reference populations which may not be applicable to very ancient individuals. We therefore chose to focus our tests on the most recently deceased of the 32 individuals in our test dataset for whom these assignments would be most meaningful. The current version of Ancestry Composition assigns windows of each chromosome to one of 45 leaf populations. These populations are nested within a hierarchical structure (Fig. S4.1). The broadest level assignments are divided into 6 categories (which we will

This is the author's version of the work. It is posted here by permission of the AAAS for personal use, not for redistribution. The definitive version was published in Science on August 4th, 2023, DOI: 10.1126/science.ade4995.

refer to as “level 1”): “Sub-Saharan African”, “East Asian & Indigenous American”, “Melanesian”, “Central & South Asian”, “Western Asian & North African” and “European”. Within each of the level 1 (Continental) categories, there are up to two additional levels of nested classifications, referred to as “level 2” (Regional) and “level 3” (Population) assignments. For instance, within the Sub-Saharan African level 1 category are the nested “African Hunter-Gatherer”, “Congolese & Southern East African”, Northern East African, and “West African” level 2 categories, and each of these (except for “African Hunter Gatherer”) contains its own nested level 3 categories. It is also possible for a proportion of an individual's genome to be classified as "Unassigned" (i.e., level 0) when a genomic window cannot be confidently assigned to a single ancestry across all levels.

We hypothesized that there were two likely ways in which Ancestry Composition assignments could be impacted by data of reduced quality, like that observed in aDNA data: (1) the total amount of ancestry assigned to any category could be reduced. This could mean that a large proportion of the individual’s ancestry would be considered “Unassigned” or their ancestry would be assigned to a lower level category, such as “Sub-Saharan African” and not to a more granular higher-level category, like “Congolese & Southern East African,” which are more difficult to predict. Although it would be ideal to have as much ancestry assigned to the highest-level category possible, we view this potential impact as preferable to the alternative option, (2) that portions of the genome would be assigned to the incorrect ancestry category (e.g., “European” instead of “Sub-Saharan African”).

This is the author's version of the work. It is posted here by permission of the AAAS for personal use, not for redistribution. The definitive version was published in Science on August 4th, 2023, DOI: 10.1126/science.ade4995.

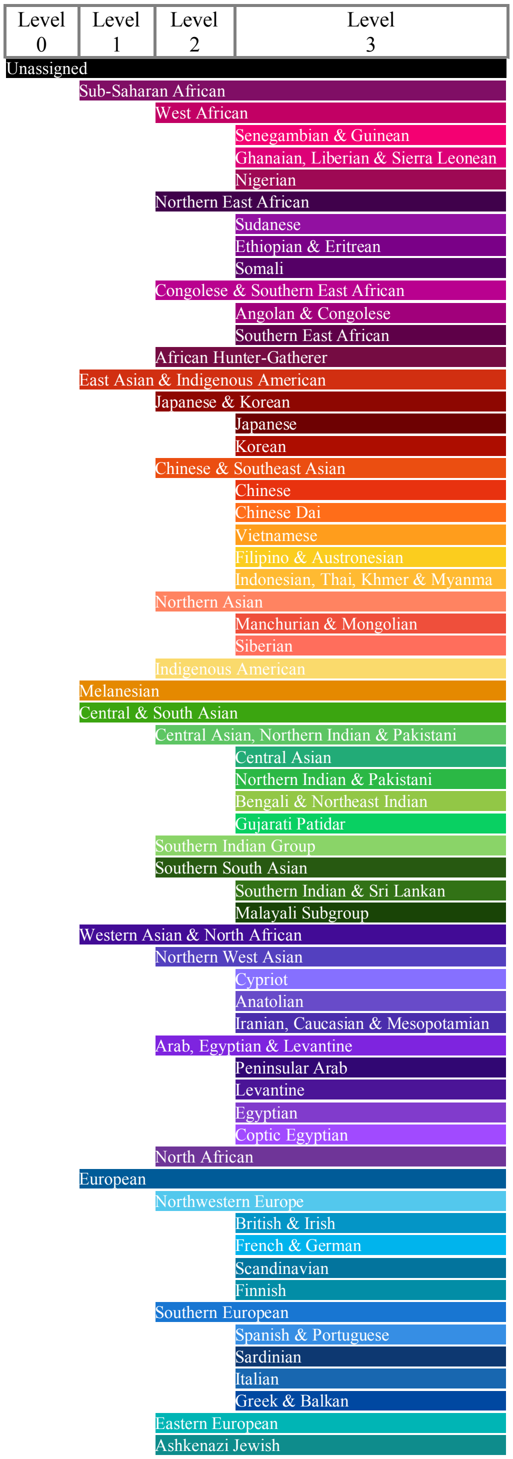

Fig. S4.1 Nested Ancestry Composition Categories. The hierarchical structure of Ancestry Composition (ac45), with higher level (more granular) categories nested beneath and to the right of the corresponding lower level (less granular) categories. Ancestry Composition categories are colored using a color palette that is consistent in all subsequent plots.

This is the author's version of the work. It is posted here by permission of the AAAS for personal use, not for redistribution. The definitive version was published in Science on August 4th, 2023, DOI: 10.1126/science.ade4995.

In order to understand whether either of these two types of errors occurred when Ancestry Composition is applied to imputed aDNA data, we first focused our analysis on individual HSJ-A-1_38.SG (a ~1015 year old individual from Iceland). As in Supplementary Text 4, we assumed that the Ancestry Composition results assigned to the highest quality versions this individual (i.e. the shotgun maximum coverage versions) represented the most accurate Ancestry Composition assignments, and that any changes to these assignments at lower coverages represented an error. We computed Ancestry Composition assignments for HSJ-A-1.SG across a variety of downsampled coverages and max(GP) thresholds. For all max(GP) thresholds, the maximum coverage versions of individual HSJ-A-1.SG are assigned 100% European ancestry (Fig. S4.2a, Table S4.1). This level 1 ancestry assignment appears most stable across all coverages at the max(GP) threshold 0.00 (i.e., without filtering out low confidence genotype calls). At 1x coverage and below an increasing proportion of the individual's ancestry could not be assigned to a level 1 category; however, very little ancestry was mis-assigned to a non-European category, even at the lowest coverages. In contrast, data produced with the other max(GP) thresholds produced deviating results at much higher coverages (Fig. S4.2b-e, Table S4.1). We therefore use the max(GP) threshold 0.00 data for the remainder of our analyses.

This is the author's version of the work. It is posted here by permission of the AAAS for personal use, not for redistribution. The definitive version was published in Science on August 4th, 2023, DOI: 10.1126/science.ade4995.

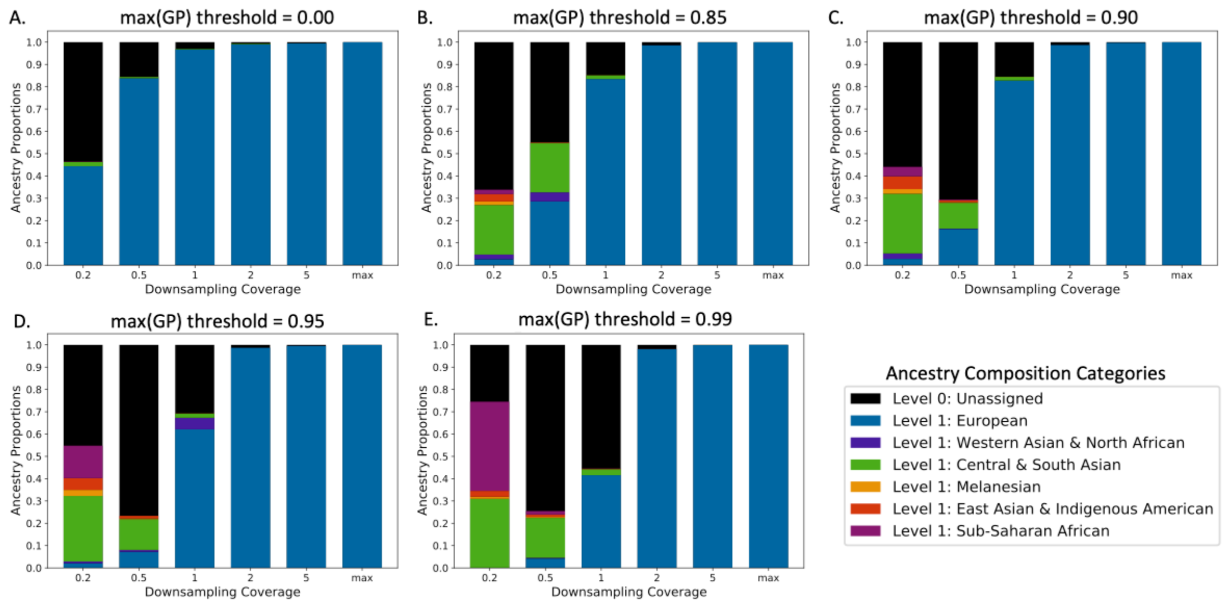

Fig. S4.2 - Level 1 Ancestry Composition Assignments for a single ancient individual (HSJ-A-1_38.SG) across multiple max(GP) thresholds. Each panel shows the total proportion ancestry assigned to each Level 1 Ancestry Composition categories across the genome of a single ancient individual (HSJ-A-1_38.SG) at a variety of sequencing approaches and coverages (Shotgun max coverage, 1240k 5x coverage, 1240k 2x coverage, 1240k 1x coverage, 1240k 0.5x coverage, 1240k 0.2x coverage). Panels A-E show the Ancestry Composition assignments using max(GP) thresholds of 0.00, 0.85, 0.90, 0.95 and 0.99, respectively.

In order to study this stability of the level 1 Ancestry Composition assignments across a variety of ancestry types, we generated Ancestry Composition assignments for 6 additional individuals across multiple downsampled coverages and again found that level 1 Ancestry Composition results can be reliably generated for ancient individuals with coverage great than or equal to 1x (Fig. S4.3, Table S4.1). For several individuals at coverages below this threshold we observe more appreciable amounts of ancestry mis-assigned to Ancestry Composition categories that differ from

This is the author's version of the work. It is posted here by permission of the AAAS for personal use, not for redistribution. The definitive version was published in Science on August 4th, 2023, DOI: 10.1126/science.ade4995.

the highest coverage assignments, suggesting that we should exercise caution when interpreting Ancestry Composition results for imputed ancient individuals with less than 1x coverage.

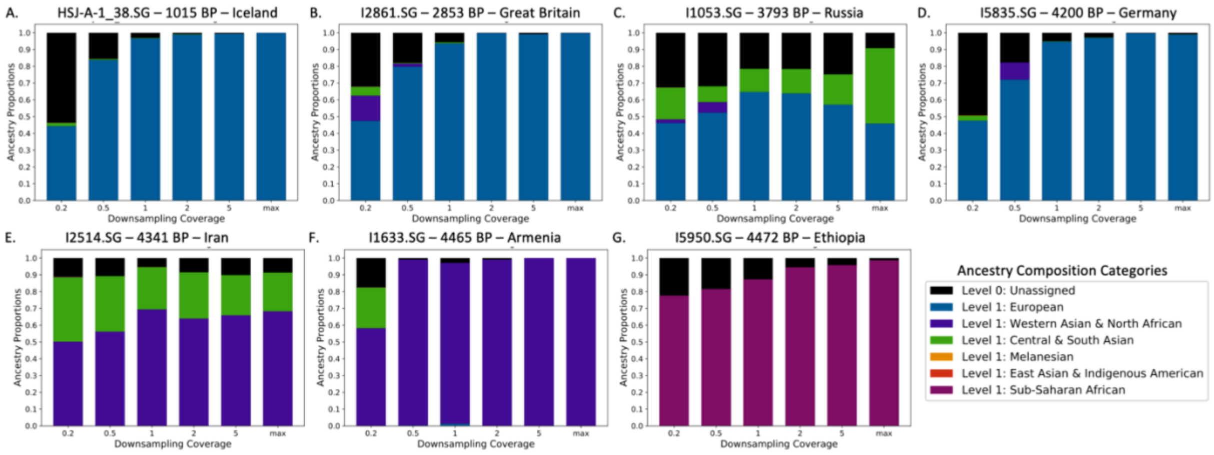

Fig. S4.3 - Level 1 Ancestry Composition Assignments for multiple ancient individuals. The total proportion ancestry assigned to each Level 1 Ancestry Composition categories across the genome of multiple ancient individuals at a variety of sequencing approaches and coverages (Shotgun max coverage, 1240k 5x coverage, 1240k 2x coverage, 1240k 1x coverage, 1240k 0.5x coverage, 1240k 0.2x coverage). Each panel displays the assignments for a different ancient individual: (**A**) HSJ-A-1_38.SG, (**B**) I2861.SG, (**C**) I1053.SG, (**D**) I5835.SG, (**E**) I2514.SG, (**F**) I1633.SG and (**G**) I5950.SG.

Since Ancestry Composition not only determines the overall proportion of ancestry assigned to each category, but also assigns ancestry to specific regions of the genome, we next painted the chromosomes of several individuals using their ancestry composition assignments at a variety of coverages and found that these level 1 ancestry assignments occured in stable locations throughout the genome (Fig. S4.4, Table S4.1).

This is the author's version of the work. It is posted here by permission of the AAAS for personal use, not for redistribution. The definitive version was published in Science on August 4th, 2023, DOI: 10.1126/science.ade4995.

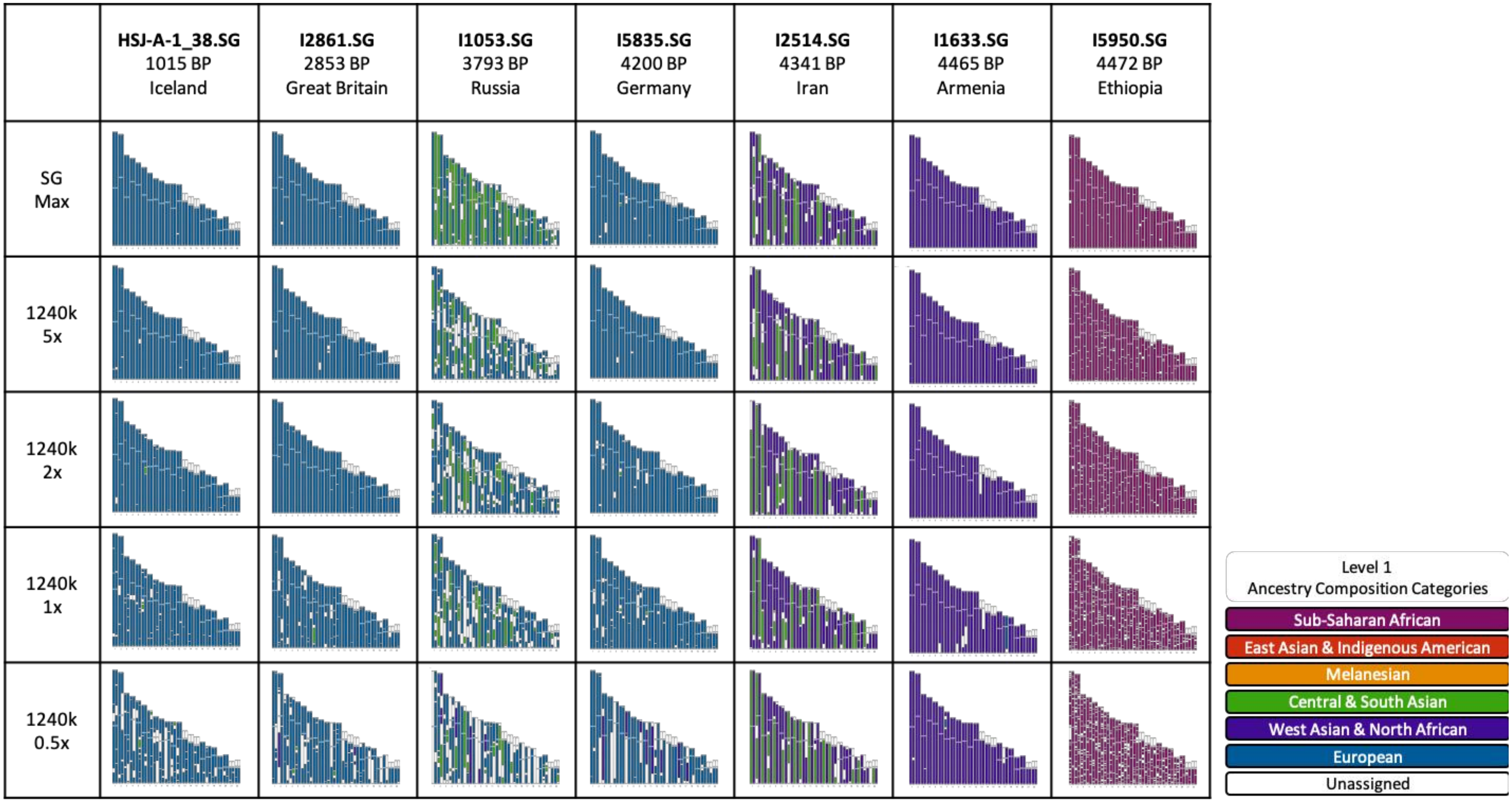

Fig. S4.4 - Chromosome paintings at a variety of coverages. Level 1 Ancestry Composition assignments visualized across the genome of seven historical individuals (left to right: HSJ-A-1_38.SG, I2861.SG, I1053.SG, I5835.SG, I2514.SG, I1633.SG, I5950.SG), at a variety of sequencing approaches and coverages (top to bottom: Shotgun max coverage, 1240k 5x coverage, 1240k 2x coverage, 1240k 1x coverage, 1240k 0.5x coverage). Chromosomes are colored according to their Level 1 Ancestry Composition category, with unassigned portions of the genome shown in white.

Next, we next considered the higher level Ancestry Composition assignments. We first considered the Icelandic individual (HSJ-38-1_A.SG) (Fig. S4.5a, Table S4.1). While only the highest coverage cases received level 2 assignments for their whole genomes, for all coverages, the only level 2 assignment was to the "Northwestern European" category. The level 3 assignments were somewhat less stable, with some ancestry assigned to the categories "British & Irish" and "Scandinavian" categories at the highest coverages. At 2x coverage and below, some ancestry was assigned to the level 3 category "French & German" suggesting that the level 3 category assignments are not reliable, even at modest (2x) coverages. Further, when we explored the Ancestry Composition assignments for individual 15950.SG–a ~4472 year old Ethiopian individual–we observed a steady increase in the amount of ancestry assigned to the level 2 "West African" category across all downsampled coverages (Fig. S4.5b, Table S4.1), suggesting that level 2 ancestry assignments are also unreliable for imputed, ancient DNA data. The reliability of level 2 and 3 Ancestry Composition assignments on aDNA data likely varies depending on the ancestry and imputation quality of the data, therefore further work may reveal that some level 2 and 3 Ancestry Composition assignments can be reliably inferred; however, in our analysis of the Catoctin individuals, we favored a conservative approach and only reported the level 1 ancestry assignments.

This is the author's version of the work. It is posted here by permission of the AAAS for personal use, not for redistribution. The definitive version was published in Science on August 4th, 2023, DOI: 10.1126/science.ade4995.

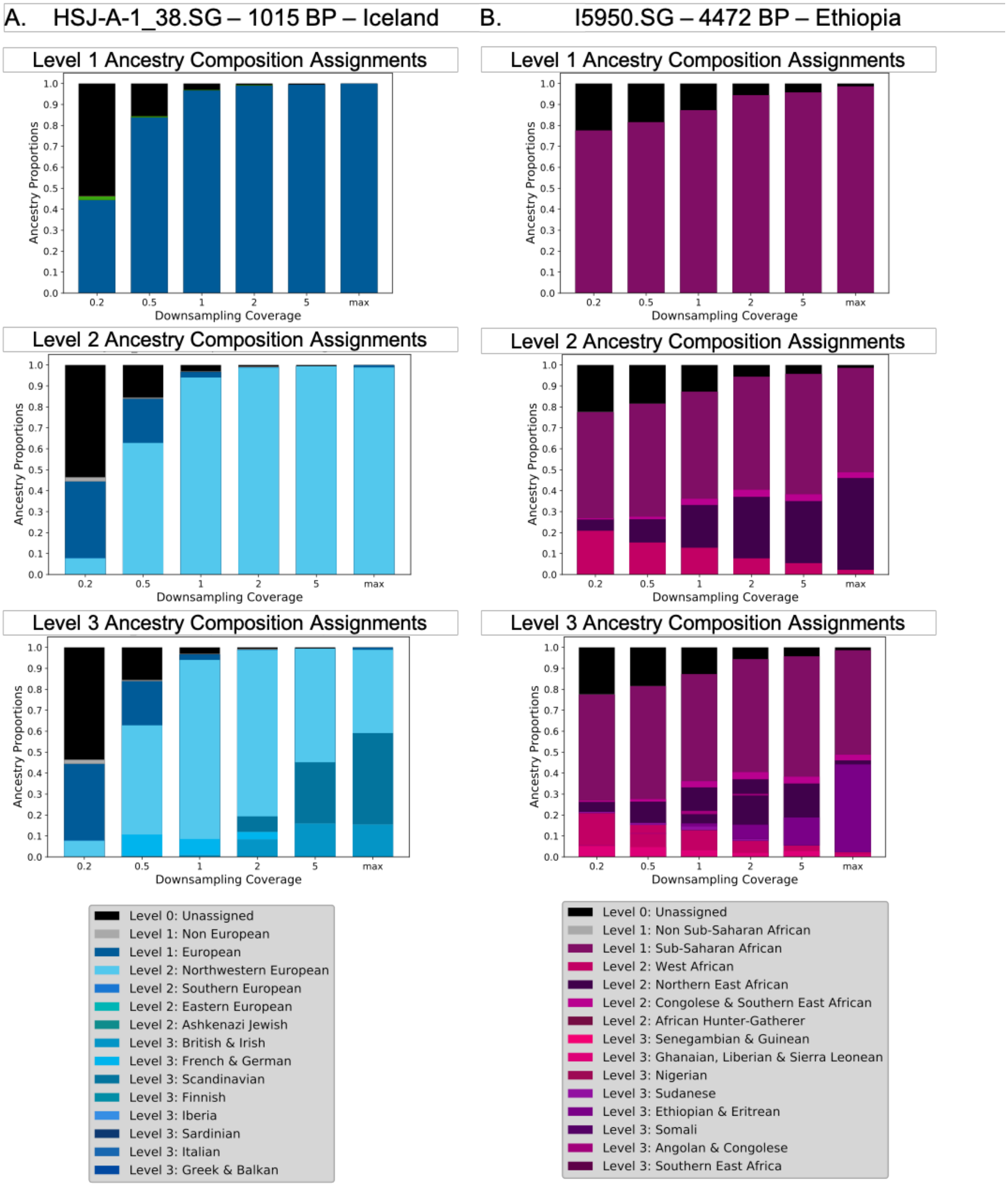

Fig. S4.5 - Level 1-3 Ancestry Composition Assignments for two ancient individuals. The total proportion ancestry assigned to Ancestry Composition categories with varying granularity across the genome for two ancient individuals (HSJ-A-1_38.SG (left) and I5950.SG (right)) at a variety

This is the author's version of the work. It is posted here by permission of the AAAS for personal use, not for redistribution. The definitive version was published in Science on August 4th, 2023, DOI: 10.1126/science.ade4995.

of sequencing approaches and coverages (Shotgun max coverage, 1240k 5x coverage, 1240k 2x coverage, 1240k 1x coverage, 1240k 0.5x coverage, 1240k 0.2x coverage). (Top row) Panels show Level 1 Ancestry Composition assignments. (Middle row) Panels show Level 2 Ancestry Composition assignments, in addition to Level 1 assignments for portions of the genome that could not be assigned to a Level 2 category. (Bottom row) Panels show Level 3 Ancestry Composition assignments, in addition to Level 1 and 2 assignments for portions of the genome that could not be assigned to a higher level category.

This is the author's version of the work. It is posted here by permission of the AAAS for personal use, not for redistribution. The definitive version was published in Science on August 4th, 2023, DOI: 10.1126/science.ade4995.

Supplementary Text S5. Genetic connections to African Among Catoctin individuals and 23andMe participants in the US cohort with at least 50% Sub-Saharan African ancestry

S5.1 The impact of applying minimum IBD segment size thresholds.

Catoctin individuals share segments of DNA that are identical-by-descent (IBD) with present-day individuals from African genetic groups that are associated with ethnolinguistic affiliation and geography (Fig. 3, Table S11). Given that Catoctin individuals were estimated to have died in Maryland between 1776-1850, they are likely recent descendants of enslaved Africans from specific regions of Africa who were forced into the Americas between the 16th and 19th century as part of the transatlantic slave trade. If Catoctin individuals are fewer generations removed from their African ancestors, they are expected to share more recent common ancestors with African genetic groups from which their ancestors were kidnapped.

To test this, we compared the IBD connections between Catoctin individuals and unique African genetic groups to the IBD connections observed between 23andMe participants in the US cohort with substantial Sub-Saharan African ancestry and unique African genetic groups. We constructed a cohort of present-day participants who have at least 50% Sub Saharan African ancestry, as estimated by 23andMe's Ancestry Composition algorithm (*42*), and who reported that all four of their grandparents were born within the United States (N = 43,696), henceforth referred to as the "US Sub-Saharan African ancestry cohort."

Using the templated positional Burrows-Wheeler transform (TWBWT) we can accurately detect small IBD segments in genotyped present-day participants (*41*); however, we set minimum segment size thresholds to identify IBD between Catoctin individuals and present-day individuals

This is the author's version of the work. It is posted here by permission of the AAAS for personal use, not for redistribution. The definitive version was published in Science on August 4th, 2023, DOI: 10.1126/science.ade4995.

to decrease false positive segments that can occur due to variable depths of coverage in historical samples (Fig. S3.2). Unsurprisingly, increasing the minimum IBD segment size threshold decreased the number of unique African groups an individual has connections with, on average (Fig. S5.1). This is expected as small segments can indicate connections to more distant common ancestors that may have existed prior to, or in the initial stages of the transatlantic slave trade. These temporally distant connections become undetectable as smaller IBD segments are pruned and connections become focused on the African groups with whom individuals share more recent common ancestors (*22*).

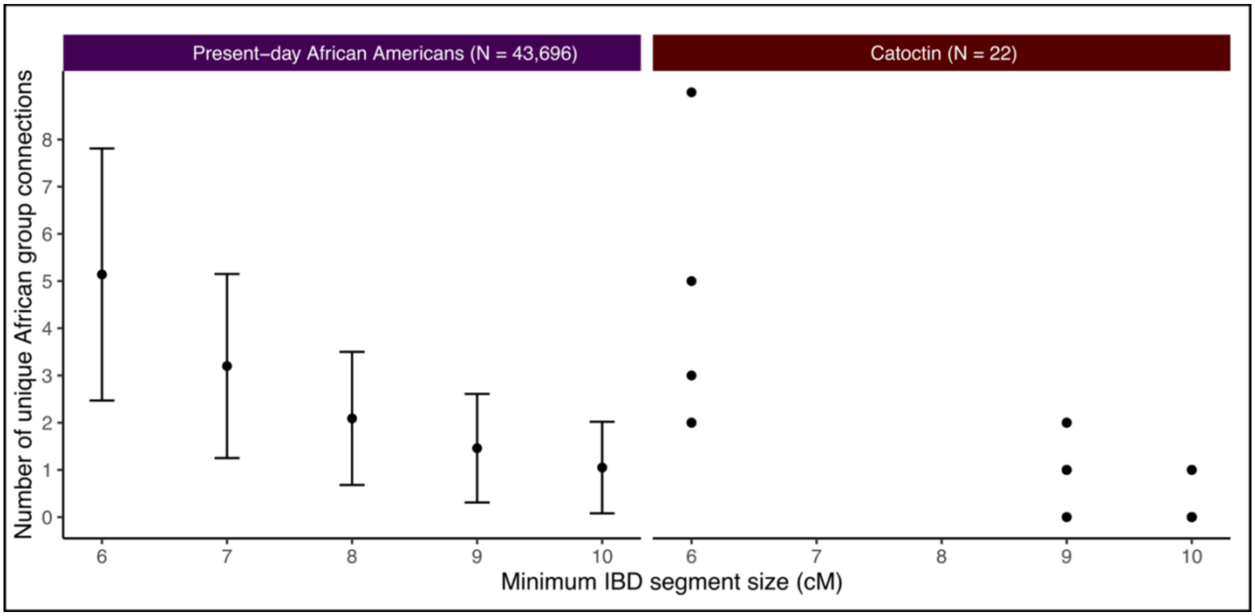

Fig. S5.1. Relationship between minimum IBD segment size threshold and number of unique African group connections. The left panel shows the mean number of connections with standard deviation bars of the US Sub-Saharan African ancestry cohort. The right panel shows the number of connections for independently plotted Catoctin individuals.

This is the author's version of the work. It is posted here by permission of the AAAS for personal use, not for redistribution. The definitive version was published in Science on August 4th, 2023, DOI: 10.1126/science.ade4995.

To account for biases that IBD segment size may have on the number of connections to African groups, we ran IBD analyses between the US Sub-Saharan African ancestry cohort and African genetic groups using 6,7,8,9, and 10 cM minimum segment size thresholds. To explore the effects African ancestry proportions may have on the number of connections to African genetic groups we parsed the US Sub-Saharan African ancestry cohort into different bins based on minimum African ancestry: ≥50% (N=43,696), ≥75% (N=28,763), and ≥95% (N=421). Together, these cohorts cover the range of African ancestry and minimum IBD segment size observed in Catoctin individuals. However, the minimum IBD segment size thresholds are variable among Catoctin individuals, depending on their depth of coverage, making direct comparisons more nuanced. To address variability in filtering thresholds, we also ran analyses on a present-day lookalike cohort whose Ancestry Composition and IBD segment size thresholds match those in the Catoctin cohort at the same frequency. In this special instance, 22 individuals (to match the sample size of Catoctin) whose ancestry and minimum IBD segment size thresholds match those of the Catoctin cohort (Table S1) were resampled 1000 times from the total present-day cohort to ensure that patterns seen in Catoctin individuals were not being driven by African ancestry proportion and segments size thresholds.

S5.2 Exploring number of connections to African genetic groups between US Sub-Saharan African ancestry cohort and Catoctin individuals

We compared the number of unique connections to African genetic groups between Catoctin individuals and various subsets of the US Sub-Saharan African ancestry cohort (Fig. S5.2). In addition to including all Catoctin individuals, we investigated the distribution of connections in Catoctin individuals that have >90% African ancestry. Catoctin individuals with more African

This is the author's version of the work. It is posted here by permission of the AAAS for personal use, not for redistribution. The definitive version was published in Science on August 4th, 2023, DOI: 10.1126/science.ade4995.

ancestry are expected to have had more recent African ancestors and therefore may share common ancestors with fewer present-day African groups.

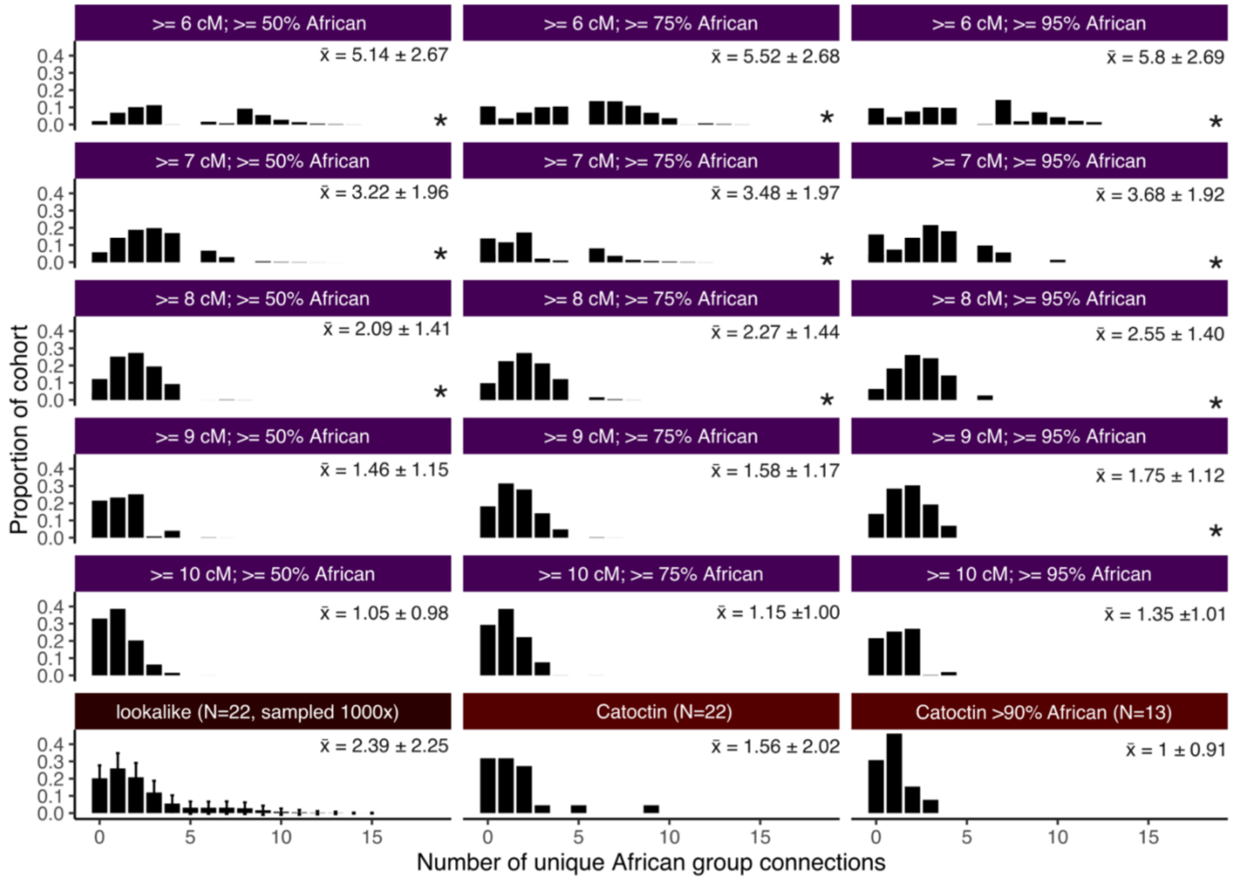

Fig. S5.2. The distribution of the number of unique African group connections in various cohorts. The mean and standard deviation of the number of unique hits are displayed in the top right of each panel. Asterisks indicate a significant ($p \leq 0.05$) difference in means between the Catoctin cohort and the corresponding panel using either a Student's t-test or Welch's t-test (depending on equal or unequal variances between datasets, respectively). The lookalike panel demonstrates the mean distribution of connections of 22 members of the US Sub-Saharan African ancestry cohort, sampled 1000 times, whose Sub-Saharan African Ancestry and minimum cM thresholds mimic those of the Catoctin cohort.

This is the author's version of the work. It is posted here by permission of the AAAS for personal use, not for redistribution. The definitive version was published in Science on August 4th, 2023, DOI: 10.1126/science.ade4995.

As expected, the distribution of unique connections to African genetic groups is widespread in present-day cohorts when retaining smaller IBD segments and skewed towards smaller values when restricting to larger segment sizes. From comparing the mean number of connections across all cohorts using a t-test, the Catoctin distribution is most similar to the ≥75% African cohorts at 9 cM (p= 0.96) and 10 cM (p = 0.83) thresholds and least similar to the ≥95% African cohort at 6 cM (p = 5.6e-13)

To determine if the distribution of connections to African genetic groups in Catoctin is smaller than expected among members of the US Sub-Saharan African ancestry cohort, we resampled the lookalike cohort 1000 times and determined the number of times the distribution of connections was significantly larger ($p<0.05$; t-test) than that of Catoctin. Though the raw mean of number of connections was higher 100% of the time, this was only significant ($p<0.05$) 50% of the time. When performing the same analysis on the Catoctin 90% African cohort, the mean of connections was higher 100% of the time and significant 84.5% of the time, indicating that individuals with more African ancestry in the Catoctin cohort tended to have fewer connections to African groups. In contrast, subsetting the US Sub-Saharan African ancestry cohort by increasing African ancestry *increased* the mean number of unique connections to African groups in every instance. This may indicate that the Catcotin individuals with high African ancestry had ancestors from the few groups they connect to, whereas members of the US Sub-Saharan African ancestry cohort have a greater chance of connecting with multiple African groups that may have admixed during and after the slave trade.

This is the author's version of the work. It is posted here by permission of the AAAS for personal use, not for redistribution. The definitive version was published in Science on August 4th, 2023, DOI: 10.1126/science.ade4995.

S5.3 Connections to specific African groups.

Self-identifying African Americans in the United States tend to have African ancestry from multiple regions of Atlantic Africa, presumably due to migrations and admixture that occurred during and after the colonial slave trade in the US (*22*). Studies have also noted an over-representation of ancestry from Nigeria when considering embarkation rates from ports along present-day Nigeria documented during the transatlantic slave trade (*22*, *122*). In general this over-representation of Nigerian ancestry is thought to be a consequence of events that occurred during the colonial slave trade, such as forced breeding in certain US states (*123*). Catoctin individuals are expected to deviate from African group connection patterns seen among members of the US Sub-Saharan African ancestry cohort because they existed prior to many migrations and admixture events.

We investigated shared IBD connections between Catoctin and African groups and found that the largest proportion of Catoctin individuals have connections to the Kongo (36%), Wolof (22%), and Mandinka (18%) groups (Fig. S5.3). Contrastingly, the largest proportion of individuals in present-day cohorts have connections to the Igbo and Kongo groups consistent with previous studies (*22*, *122*).

This is the author's version of the work. It is posted here by permission of the AAAS for personal use, not for redistribution. The definitive version was published in Science on August 4th, 2023, DOI: 10.1126/science.ade4995.

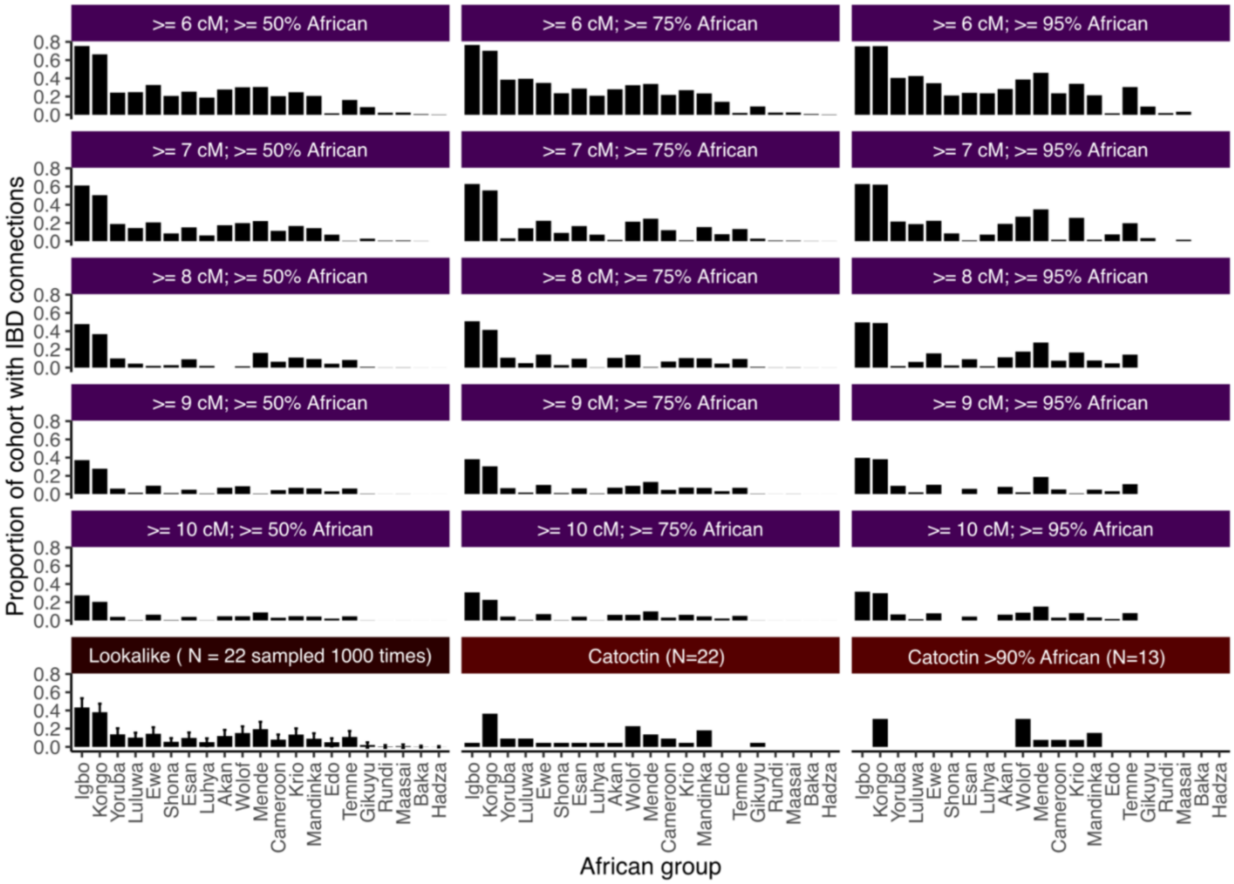

Fig. S5.3. The proportion of individuals with connections to specific African groups across all tested cohorts. The lookalike panel demonstrates the mean proportion of African group connections of 22 members of the US Sub-Saharan African ancestry cohort, sampled 1000 times, whose African Ancestry and minimum cM thresholds mimic those of the Catoctin cohort.

To test if the distribution of African connections seen in Catoctin is significantly enriched for specific African groups, we calculated the number of times the count of individuals with connections to each African group connection was matched both between Catoctin and the lookalike dataset across 1000 iterations. From this analysis, the most striking difference between the distributions is the underrepresentation of connections to the Igbo group in Catoctin individuals (p<0.001; Table S5.3). That is to say, the distribution of Igbo connections seen in the

This is the author's version of the work. It is posted here by permission of the AAAS for personal use, not for redistribution. The definitive version was published in Science on August 4th, 2023, DOI: 10.1126/science.ade4995.

Catoctin cohort (just a single connection) was observed 0 times across all subsampling analyses. Though the Catoctin group shows enrichment for the Mandinka, Kongo, and Wolof groups, it is not until the Catoctin cohort is subset to >90% African ancestry that these groups are significantly enriched.

Records from the transatlantic slave trade indicate that the majority of enslaved Africans brought to Maryland embarked from Senegambia and West Central Africa (*1*) (Table S9). Comparing these records to connections to African groups provides support that at least a portion of the Catoctin individuals had recent African ancestors brought to the US from Senegambia (where Wolof ancestors would have disembarked from) and West Central Africa (where Kongo ancestors would have disembarked from). It is more likely that the Catoctin individuals with more African ancestry and less European admixture were those with the recent ancestors embarking from the central and northwestern coasts of Africa.

This is the author's version of the work. It is posted here by permission of the AAAS for personal use, not for redistribution. The definitive version was published in Science on August 4th, 2023, DOI: 10.1126/science.ade4995.

African Group	Catoctin (N=22)		Catoctin >90% African (N=13)	
	p higher rep	*p lower rep*	*p higher rep*	*p lower rep*
Akan	0.958	0.231	0.777	0.578
Baka	1	0.964	1	0.991
Cameroon	0.571	0.743	0.256	0.945
Edo	1	0.284	1	0.535
Esan	0.909	0.337	0.681	0.718
Ewe	0.969	0.149	0.845	0.481
Gikuyu	0.404	0.902	0.183	0.993
Hadza	1	0.98	1	0.988
Igbo	1	**<0.0001**	0.998	**0.009**
Kongo	0.659	0.517	0.079	0.971
Krio	0.961	0.168	0.879	0.437
Luhya	0.729	0.671	0.441	0.933
Luluwa	0.735	0.592	0.269	0.961
Maasai	1	0.837	1	0.892
Mandinka	0.112	0.968	**0.008**	1
Mende	0.838	0.364	0.523	0.731
Rundi	1	0.89	1	0.968
Shona	1	0.24	1	0.577
Temne	1	0.067	1	0.174
Wolof	0.213	0.917	**0.027**	0.992
Yoruba	0.838	0.376	0.509	0.802

Table S5.3. Significant over- and under-representation of African groups that Catoctin (N=22) and Catoctin > 90% African ancestry (N=13) connected to. *p higher* represents the proportion of times the present-day lookalike cohort had the exact same number or fewer number of individuals with connections to each African group over 1000 iterations (lower values correspond to higher representation in Catoctin) ; proportions less than <0.05 are bolded *p lower* represents the proportion of times the present-day lookalike cohort had the exact same number or

This is the author's version of the work. It is posted here by permission of the AAAS for personal use, not for redistribution. The definitive version was published in Science on August 4th, 2023, DOI: 10.1126/science.ade4995.

greater number of individuals with connections to each African group over 1000 iterations (lower values correspond to lower representation in Catoctin); proportions less than <0.05 are bolded.

Supplementary Text S6. A method for inferring relationships and pedigrees when individuals have low coverages

S6.1 Likelihoods for low-coverage pedigrees

The most likely ways of connecting historical and present-day pedigrees were inferred using a composite likelihood approach. First, pedigrees connecting present-day 23andMe research participants were inferred using the software Bonsai (Jewett et al, 2021). We then considered all plausible ways of connecting the most recent common ancestral node in each present-day pedigree to a node on the historical pedigree (Fig. S6.1). All possible points of connection on the historical pedigree were considered, assuming outbred pedigrees, and a broad range of possible connection degrees, *d*, covering the range of realistic historical-to-present-day connecting lineage degrees.

For each possible point of connection and degree, d, we computed the composite likelihood, L_A, of the arrangement, A, as

$$L_A = \prod_{i,j} \quad \hat{L}^{g,c}_{i,j} L^{a}_{i,j} \quad , \qquad \text{(Eq. 6.1)}$$

Where $L^{a}_{i,j} = f(a_i - a_j \mid R_{i,j})$ is the probability density function of the difference in ages a_i and a_j of individuals i and j and $L^{g,c}_{i,j} = Pr(I_{i,j} \mid R_{i,j}, c)$ is the probability of the observed IBD $I_{i,j}$ between individuals i and j, given that their pairwise relationship is $R_{i,j}$ and given that the genotypes of one of the individuals were obtained from sequencing data with coverage c. $\hat{L}^{g,c}_{i,j}$ is the composite likelihood approximation of $L^{g,c}_{i,j}$ given by

This is the author's version of the work. It is posted here by permission of the AAAS for personal use, not for redistribution. The definitive version was published in Science on August 4th, 2023, DOI: 10.1126/science.ade4995.

$$\hat{L}_{i,j}^{g,c} = Pr(N_{i,j} \mid R_{i,j}, c)\, Pr(T_{i,j} \mid R_{i,j}, c), \qquad \text{(Eq. 6.2)}$$

where $N_{i,j}$ is the number of segments observed between individuals i and j and $T_{i,j}$ is the total length of IBD observed between individuals i and j. When computing the approximation $\hat{L}_{i,j}^{g} = Pr(N_{i,j} \mid R_{i,j})\, Pr(T_{i,j} \mid R_{i,j})$, we used the analytical formulas for $Pr(N_{i,j} \mid R_{i,j})$ and $Pr(T_{i,j} \mid R_{i,j})$ derived by Huff et al (*119*), but we corrected these formulas to account for the fact that the genomic data for the historical individuals was low coverage, as described in section S6.2. The pairwise age likelihoods were computed using distributions trained with 23andMe research participant data as described in Jewett et al (*43*). Age distributions were extended to arbitrarily distant relationships as follows: denote a relationship between i and j by $R_{i,j} = (u, d, n)$ using the notation of Ko and Nielsen (*124*). Here, u is the number of meioses between i and their common ancestor with j, d is the number of meioses between j and their common ancestor with i, and n is the number of common ancestors of i and j. If $E[\triangle\, a_{p,c}]$ and $var(\triangle\, a_{p,c})$ are the empirically observed mean and variance of the difference in age between a parent and child, then the age difference distribution between a pair, i and j, with relationship $R_{i,j}$ is modeled as a Gaussian random variable with mean $(u - d)E[\triangle\, a_{p,c}]$ and variance $(u + d)var(\triangle\, a_{p,c})$.

This is the author's version of the work. It is posted here by permission of the AAAS for personal use, not for redistribution. The definitive version was published in Science on August 4th, 2023, DOI: 10.1126/science.ade4995.

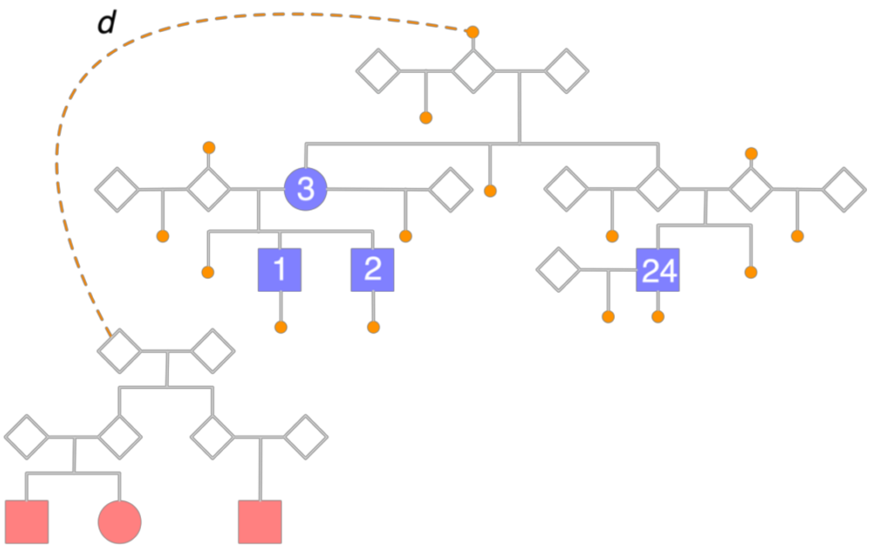

Fig S6.1: Determining the most likely way of connecting a present-day and historical pedigree. Genotyped individuals in the present-day pedigree are shaded in red. Genotyped individuals in the historical pedigree are shaded in purple. The lineage connecting the present-day and historical pedigrees is shown as a dashed orange line. All locations where this lineage can attach to the historical pedigree are shown as small orange dots. The degree of the lineage is denoted as *d*. We connected the present-day and historical pedigrees through each possible attachment point and a range of values for *d*. The composite likelihood (Equation SE6.1) was computed for each connection point and value of *d*.

S6.2: Correcting IBD probabilities for low coverage data

Here, we derive the probabilities $Pr(N_{i,j} \mid R_{i,j}, c)$ and c of the number and total length of segments shared between a pair of individuals i and j when the genotypes of one individual were obtained from low coverage imputed sequencing data with coverage c.

Low coverage imputed data introduces both false negative and false positive segments. False negative segments arise because missing genotypes or genotyping errors occur within true IBD (or identical-by-state) segments, causing them to be missed by an IBD detection algorithm. For low coverage imputed data,

This is the author's version of the work. It is posted here by permission of the AAAS for personal use, not for redistribution. The definitive version was published in Science on August 4th, 2023, DOI: 10.1126/science.ade4995.

false positive segments arise largely due to imputation, which can cause two regions to be identical-by-state over a region that is longer than the minimum threshold set by the IBD detection algorithm. Thus, when considering IBD called on genotypes inferred from imputed low coverage data, we must jointly model the IBD arising from Mendelian inheritance, as well as missing and spurious IBD arising from the low coverage data.

Huff et al (*119*) derived a formula for $Pr(N_{i,j} \mid R_{i,j})$ for the case in which the observed IBD $I_{i,j}$ between individuals i and j was the true IBD without false positive or false negative segments and they derived a joint likelihood for the total number of segments together with the set of segment lengths, allowing for a given false positive segment rate and mean false positive segment length. In contrast to the approach by Huff et al, we don't consider individual segment lengths, which can be noisy in the case of low coverage DNA. Thus, we do not include a term in the genetic likelihood for the probability of all observed segment lengths, and instead consider the total IBD length, which may be more robust to IBD inference errors such as the fragmentation of segments.

We take the alternate approach of incorporating the false negative rates into the formulas for $Pr(N_{i,j} \mid R_{i,j})$ and $Pr(T_{i,j} \mid R_{i,j}, c)$ directly. We assume that the false positive rate based on coverage is small and only exerts a significant influence on the likelihood of a pairwise relationship when the two individuals are truly unrelated. This assumption is reasonably well justified by the low observed average false positive rate of < 0.2 false positive segments per pair, shown in Fig. S6.4A. Using this assumption, we derive one set of formulas for $Pr(N_{i,j} \mid R_{i,j}, c)$ and $Pr(T_{i,j} \mid R_{i,j}, c)$ for the case in which $R_{i,j}$ denotes an unrelated pair and we derive a separate set of formulas for $Pr(N_{i,j} \mid R_{i,j}, c)$ and $Pr(T_{i,j} \mid R_{i,j}, c)$ for the case in which $R_{i,j}$ denotes a pair of truly related individuals. For the case in which i and j are unrelated, the influence of low coverage on the distributions of $N_{i,j}$ and $T_{i,j}$ comes solely from false positive segments arising from the

This is the author's version of the work. It is posted here by permission of the AAAS for personal use, not for redistribution. The definitive version was published in Science on August 4th, 2023, DOI: 10.1126/science.ade4995.

imputation process. For the case in which i and j are related, the influence of low coverage on the distributions of $N_{i,j}$ and $T_{i,j}$ comes solely from false negative segments arising from genotype error.

S6.3: The distributions of $\mathbf{N_{i,j}}$ and $\mathbf{T_{i,j}}$ for related individuals

In deriving formulas for $Pr(N_{i,j} \mid R_{i,j}, c)$ and $Pr(T_{i,j} \mid R_{i,j}, c)$, we will use two results that we demonstrate in Section S6.4. First, the false negative rate for a segment of length l – i.e., the probability that a segment of true length l is unobserved when one individual has coverage c – can be modeled as

$$Pr(O \mid c, l) \approx 1 - e^{-qcl}, \qquad \text{(Eq. 6.3)}$$

for some constant q. Second, we demonstrate that the average observed length l_o of a segment with true length l_t is approximately given by

$$E_c[l_o \mid l_t] \approx (1 - e^{-pc})(l_t - \tau) + \tau, \qquad \text{(Eq. 6.4)}$$

for some constant p, where τ is the minimum threshold length for an IBD segment to be called. Using Equations (Eq. 6.3) and (Eq. 6.4), we now derive $Pr(N_{i,j} \mid R_{i,j}, c)$ and $Pr(T_{i,j} \mid R_{i,j}, c)$.

As in Huff et al (*119*), we model the number $N_{i,j}$ of segments shared between individuals i and j as a Poisson random variable. The parameter $\lambda_{R,c}$ of the Poisson distribution is equal to the expected number of segments shared between individuals of relationship $R = (u, d, n)$ when one of the individuals has coverage c. The expected number of segments when the coverage is c is equal to the expected number of segments at full coverage, times the probability that a segment is observed between two people with relationship $R_{i,j}$ when the coverage of one of them is c. Let O_R be the event that a given segment is observed between a pair of individuals with relationship R. Then we have

This is the author's version of the work. It is posted here by permission of the AAAS for personal use, not for redistribution. The definitive version was published in Science on August 4th, 2023, DOI: 10.1126/science.ade4995.

$$\lambda_{R,c} = \lambda_R Pr(O_R |\, R, c),$$

where Huff et al (*119*) showed that

$$\lambda_R \approx n \frac{((u+d)r + 22)}{2^{(u+d)-1}} e^{-(u+d)\tau/100}, \qquad \text{(Eq. 6.5)}$$

where $r \approx 35.5$ is the expected number of meioses in the autosomal genome, 22 is the number of autosomes, and τ is the threshold in cM below which IBD segments are not considered. The probability $Pr(O_R |\, R, c)$ can be obtained by integrating over the true length of the segment:

$$Pr(O_R |\, R, c) = \int_{l\ =0}^{\infty} Pr(O \,|\, l\ \ , c) f_R(l\ \ )\, dl_t$$

$$= \int_{l\ =0}^{\infty} (1 - e^{-qcl}\ \ )\, 1_{l \geq \tau}\, f_R(l\ \ )\, dl$$

$$= \int_{l\ =\tau}^{\infty} (1 - e^{-qcl}\ \ ) \frac{(u+d)}{100} e^{-(u+d)l\ \ /100}\, dl$$

$$= e^{-(u+d)\tau/100} - \int_{l\ =\tau}^{\infty} \frac{(u+d)}{100} e^{-(\frac{u+d}{100}+qc)l}\, dl$$

$$= e^{-(u+d)\tau/100} - \frac{(u+d)}{100}(\frac{u+d}{100} + qc)^{-1}\, e^{-(\frac{u+d}{100}+qc)\tau}, \qquad \text{(Eq. 6.6)}$$

where the density $f_R(l\ \ )$ comes from Huff et al (*119*) and $1_{l \geq \tau}$ is the indicator function taking the value 1 whenever $l \geq \tau$ and 0, otherwise. In the second equality, we have assumed that the observed segment is not longer than the true segment because we are only considering false negative rates. Thus, the true segment must be longer than τ to be observed.

This is the author's version of the work. It is posted here by permission of the AAAS for personal use, not for redistribution. The definitive version was published in Science on August 4th, 2023, DOI: 10.1126/science.ade4995.

We can check the formula for $Pr(O_R|\, R, c)$ by noting that as the coverage approaches infinity, the formula for $Pr(O_R|\, R, c)$ approaches $e^{-(u+d)\tau/100}$, which is the probability that the true segment has at least length τ. As the relationship $R = (u, d, n)$ becomes increasingly distant – i.e., as $u + d$ goes to infinity – the formula for $Pr(O_R|\, R, c)$ approaches $e^{-(u+d)\tau/100}(1 - e^{-qc\tau})$, which is the probability that the true segment has length at least τ and that the segment of length τ is observed between the low coverage individuals, regardless of the relationship.

To find the expected total IBD length $T_{R,c}$ between two individuals separated by relationship $R = (u, d, n)$ when one individual is full coverage and the other individual has coverage c, let $\mu_{R,c}$ be the mean segment length observed between the two individuals. Then we can approximate $E[T_{R,c}]$ by the product of $\mu_{R,c}$ and the expected number of segments $\lambda_{R,c}$:

$$E[T_{R,c}] \approx \mu_{R,c}\lambda_{R,c}. \qquad \text{(Eq. 6.7)}$$

Using the relationship $E_c[l_o \mid l_t] \approx (1 - e^{-pc})(l_t - \tau) + \tau$ from Equation (Eq. 6.4) we find that

$$\begin{aligned} \mu_{R,c} &= (1 - e^{-pc})(E_R[L_t] - \tau) + \tau \\ &= (1 - e^{-pc})(\frac{100}{u+d} - \tau) + \tau, \end{aligned} \qquad \text{(Eq. 6.8)}$$

where the formula $E_R[L_t] = \frac{100}{u+d}$ comes from the result that segments between two individuals of relationship $R = (u, d, n)$ have expected length $\frac{100}{u+d}$ when expressed in centimorgans (*119*).

To derive an approximation of the variance of the observed total IBD $T_{R,c}$, we make the simplifying assumption that all variability in $T_{R,c}$ arises from the lengths of the true segments T_R. In other words, we

This is the author's version of the work. It is posted here by permission of the AAAS for personal use, not for redistribution. The definitive version was published in Science on August 4th, 2023, DOI: 10.1126/science.ade4995.

assume that $l_o \approx (1 - e^{-pc})(l_t - \tau) + \tau$, so that $T_{R,c} \approx (1 - e^{-pc})(T_R - N\tau) + N\tau$, where N is the number of segments. Thus, we have

$$var(T_{R,c}) \approx (1 - e^{-pc})^2 var(T_R). \qquad \text{(Eq. 6.9)}$$

An approximate formula for $var(T_R)$ was derived in Jewett et al (*43*). For two related individuals with relationship R, we model the total IBD as a Gaussian random variable with mean $E[T_{R,c}]$ and variance $var(T_{R,c})$.

S6.4: The distributions of $\mathbf{N}_{i,j}$ and $\mathbf{T}_{i,j}$ for unrelated individuals

When individuals i and j are unrelated, we assume that the observed segments between them arise entirely from false positive segments due to imputation or other sources. In Section S6.5, we demonstrate that the expected number of false positive segments when one individual is high coverage and the other individual has low coverage c can be modeled as

$$E[N_{i,j}] \approx \gamma_1/c \qquad \text{(Eq. 6.10)}$$

for some constant γ_1, and we show that $var[N_{i,j}] \approx \gamma_1/c$ for the same constant γ_1, suggesting that $\boldsymbol{N_{i,j}}$ is reasonably modeled by a Poisson distribution. Thus, for two unrelated individuals i and j, we model $\boldsymbol{N_{i,j}}$ as a Poisson random variable with parameter γ_1/c.

In Section S6.5, we also demonstrate that the expected total false positive IBD between two unrelated individuals has mean

$$E[T_{i,j}] \approx \gamma_2/c \qquad \text{(Eq. 6.11)}$$

This is the author's version of the work. It is posted here by permission of the AAAS for personal use, not for redistribution. The definitive version was published in Science on August 4th, 2023, DOI: 10.1126/science.ade4995.

and that the variance is approximately

$$var(T_{i,j}) \approx \gamma_3/c \qquad \text{(Eq. 6.12)}$$

for constants γ_2 and γ_3. Thus, for two unrelated individuals, we model the total IBD $T_{i,j}$ as a Gaussian random variable with mean γ_2/c and variance γ_3/c.

Section S6.5: False positive and false negative rates of IBD

Using the same data set described in Supplementary Text S3, we computed true-positive, false-positive, true-negative, and false-negative rates as a function of coverage, as well as the relationship between the true and observed segment lengths.

In Equation (Eq. 6.6), we used a simple and integrable expression for the per-segment true positive rate $Pr(O \mid c, l)$ as a function of the true segment length and coverage. The form of this equation comes from the empirical true-positive rate as a function of coverage (Fig. S6.2). By minimizing the mean squared error

This is the author's version of the work. It is posted here by permission of the AAAS for personal use, not for redistribution. The definitive version was published in Science on August 4th, 2023, DOI: 10.1126/science.ade4995.

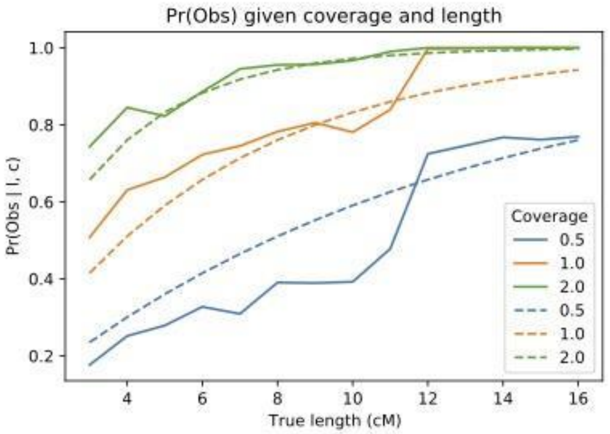

Fig S6.2: True-positive rate of observing an IBD segment of a given length for several different coverages. Solid curves show empirical values. Dashed curves show fitted values using Equation (SE6.3).

between the empirical and fitted values in Equation (Eq. 6.3), we found that a value of q = 0.179 provided a good fit to the empirical data.

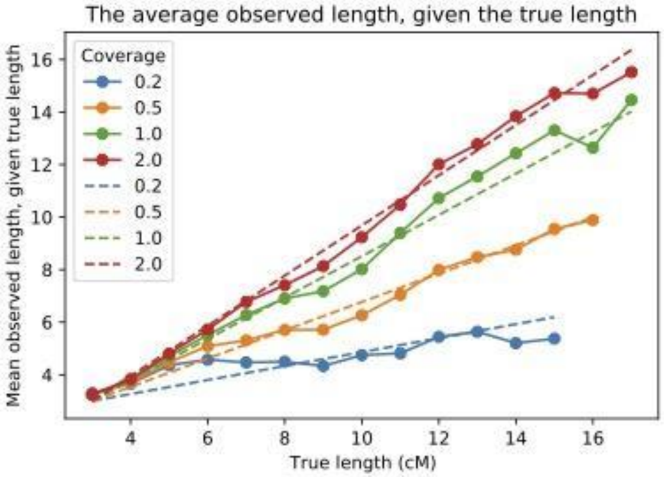

Fig S6.3: Empirically-observed mean segment lengths as a function of the true segment length. Shown by coverage. Solid curves correspond to the empirical data. Dashed curves are the fitted values.

This is the author's version of the work. It is posted here by permission of the AAAS for personal use, not for redistribution. The definitive version was published in Science on August 4th, 2023, DOI: 10.1126/science.ade4995.

The form of Equation (Eq. 6.4) also comes from the empirical data. We observed that the mean observed length was approximately linear in the true length, with a constant that depended on the coverage. We found that the relationship in Equation (Eq. 6.4) captured the empirical relationship. By minimizing the mean squared error between the predicted and empirical mean observed lengths using Equation (Eq. 6.4), we found that a value of p = 1.79 provided a good fit for the data (Fig. S6.3).

This is the author's version of the work. It is posted here by permission of the AAAS for personal use, not for redistribution. The definitive version was published in Science on August 4th, 2023, DOI: 10.1126/science.ade4995.

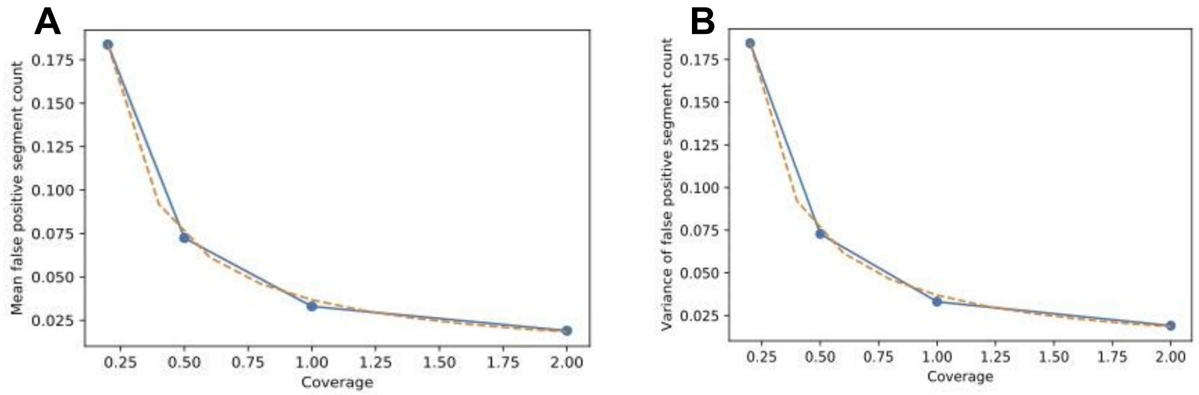

Figure S6.4: Mean (A) and variance (B) in the number of false-positive segments observed between a pair of individuals as a function of the coverage of one of the individuals. Solid curves show empirical values, dashed curves minimize the mean squared error between empirical and fitted values. Both fitted curves are 0.036/c.

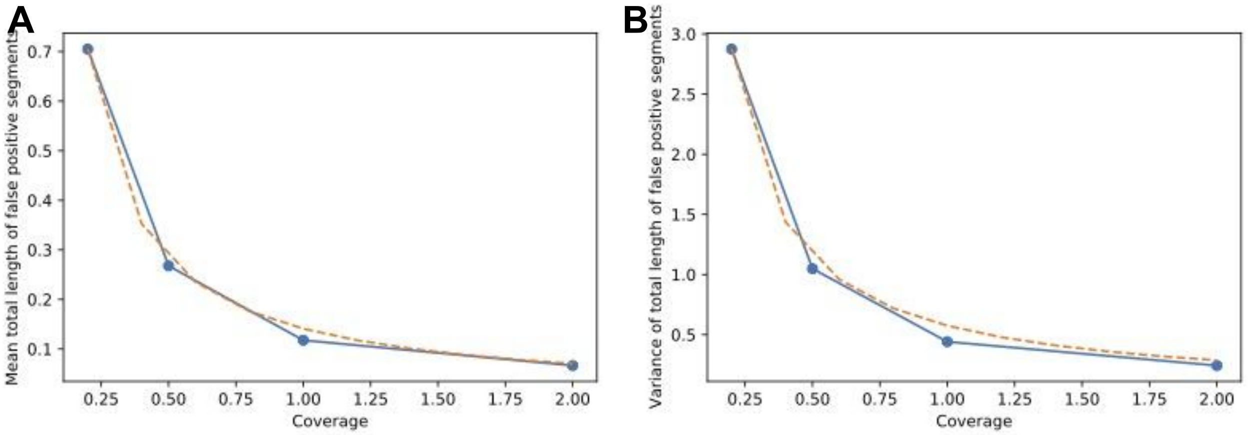

Figure S6.5: **Mean (A) and variance (B) in the total observed length of false-positive IBD between a pair of individuals, one of whom has coverage c.** Solid curves indicate empirical values and dashed curves indicate fitted values.

We found that the number of observed false-positive segments as a function of coverage is well modeled by Equation (Eq. 6.10). Furthermore, from Figs. S6.4A and S6.4B, it can be seen that both the mean and variance are well approximated by γ_1/c, for the same value of $\gamma_1 = 0.036$. Thus, the expected number of false-positive segments can be modeled by a Poisson random variable with parameter γ_1/c.

This is the author's version of the work. It is posted here by permission of the AAAS for personal use, not for redistribution. The definitive version was published in Science on August 4th, 2023, DOI: 10.1126/science.ade4995.

Finally, the expected total length of false-positive IBD is well approximated by Equation (Eq. 6.11) and its variance is well approximated by Equation (Eq. 6.12), as can be seen in Figs S6.5A and S6.5B. By minimizing the mean squared error between the empirical and predicted values, we found that values of $\gamma_2 = 0.141$ and $\gamma_3 = 0.575$ provided a good fit.

S6.6: Pedigree inference

Present-day pedigrees were inferred among individuals sharing at least a threshold amount of IBD with a historical individual in a given Catoctin family. The threshold was the one chosen to minimize false positive IBD sharing, given in Section S3.2 in Supplementary Text S3. Present-day family sets were first created by grouping individuals who shared at least 200 cM of IBD with at least one other person in the group. The individual in each group with the highest average sharing to all other group members was chosen as the focal individual and the pedigree of the group was inferred using Bonsai (*43*). We then identified the common ancestor of the present-day pedigree and exhaustively considered all positions to which the lineage extending from the common ancestor could attach in the historical family's pedigree (Fig. S6.1). Positions were either lineages extending up from an unsampled individual or down from any individual and the possible partners of the individual. Lineages extending directly down from individuals in Fig. S6.1 are short-hand for lineages extending down from the individual and a partner.

For each position in the historical family, we considered lineages extending from the most recent common ancestor of the present-day pedigree to the position on the historical pedigree. We considered degrees ranging from 0 to 15 degrees, where 0 degrees indicated that the common ancestor of the present-day pedigree was an individual in the historical pedigree. The likelihood of the attachment point and degree was computed using Equation (Eq. 6.1).

The probability that a present-day pedigree attached to a given position on the historical pedigree was computed by assuming that the prior probability of attachment to any point on the historical family's

This is the author's version of the work. It is posted here by permission of the AAAS for personal use, not for redistribution. The definitive version was published in Science on August 4th, 2023, DOI: 10.1126/science.ade4995.

pedigree was uniform across all attachment points. Let D denote the data (IBD segments and ages) and let A denote the arrangement (degree of relationship and point of connection on the historical pedigree). Then we have

$$Pr(A \mid D) = \frac{Pr(D|A)\, Pr(A)}{Pr(D)} \propto Pr(D|A) = L_A \qquad \text{(Eq. 6.13)}$$

where L_A is given in Equation (Eq. 6.1). Thus $Pr(A \mid D)$ can be computed by normalizing the values of L_A. When computing Equation (Eq. 6.1), we took the age of each historical individual to be their estimated age at burial, plus the time difference between the present year and their date of burial (Table S1).

The probability of any pedigree connecting through a given arrangement A is then

$$Pr(A) = \sum_{ped} \quad Pr(A|D_{ped})Pr(ped) = \frac{1}{P}\sum_{ped} \quad Pr(A \mid D_{ped}), \qquad \text{(Eq. 6.14)}$$

where *ped* indicates a particular present-day pedigree, D_{ped} denotes the data for the pedigree, and P is the number of present-day pedigrees. The probabilities $Pr(A)$ are the values shown in Fig. 6 in the main text.

S6.7: Ancestries of pedigrees

The ancestry of each present-day individual connected to a historical family was computed using the Ancestry Composition local ancestry inference method (*42*) and global ancestry proportions were aggregated with a confidence threshold of 51%. We computed the admixture fraction f_p^A for population p on lineage A as

$$f_p^A = \sum_{i=1}^{N_A} \quad Pr(p \mid i)\, Pr(i) = \frac{1}{N_A}\sum_{i=1}^{N_A} \quad Pr(p \mid i), \qquad \text{(Eq. 6.15)}$$

This is the author's version of the work. It is posted here by permission of the AAAS for personal use, not for redistribution. The definitive version was published in Science on August 4th, 2023, DOI: 10.1126/science.ade4995.

where N_A is the number of individuals whose most likely connection is through arrangement A and $Pr(p|i)$ is the admixture fraction for population p of individual i. These are the values in the pie charts shown in Fig. 6.

S6.8: Resampling test of present-day pedigrees with American ancestry related to Catoctin Family A

The ancestries of pedigrees attached through different lineages is informative about the accuracy of the method for connecting present-day and historical pedigrees through different arrangements. Ancestry information was not used when computing the probabilities of arrangements and can therefore be used as a check. In particular, for Family A, we know that individual *f* must have had a significant amount Indigenous American ancestry because their children, 1 and 2, have Indigenous American ancestry that is not observed in their parent, 3 (Fig. 2). We do not observe high proportions of Indigenous American ancestry in individual 24 in Catoctin Family A.

This high Indigenous American ancestry in individual *f* allows us to test whether connections of present-day pedigrees to Family A through individual *f* are consistent with their known recent Indigenous American ancestry. In particular, we expect that individual *f* has collateral relatives shared through their Indigenous American ancestors. We expect that such relatives would be enriched among the set of pedigrees related to Catoctin Family A that have particularly high Indigenous American ancestry.

We considered present-day pedigrees whose sampled individuals had at least twice as much inferred Indigenous American ancestry as African ancestry. Of 149 present-day pedigrees that satisfied this criterion, all but one pedigree had a most likely connection through individual *f*. To evaluate whether this result was statistically significant, we considered the mean of the 149 log likelihoods of connecting through individual *f*. We repeatedly sampled 149 pedigrees from among the set of pedigrees with

This is the author's version of the work. It is posted here by permission of the AAAS for personal use, not for redistribution. The definitive version was published in Science on August 4th, 2023, DOI: 10.1126/science.ade4995.

$f_{American}/f_{African} < 2$ and computed the mean log likelihood of attaching through individual *f*. Out of 1,000,000 samples of 149 such pedigrees, we found that no sample had a mean log likelihood as high as the mean log likelihood in the high American ancestry pedigrees. This yields a p-value for the resampling test of $p < 10^{-6}$.

This is the author's version of the work. It is posted here by permission of the AAAS for personal use, not for redistribution. The definitive version was published in Science on August 4th, 2023, DOI: 10.1126/science.ade4995.

Supplementary Figs

Y-Haplogroup Distribution

Haplogroup A1b1
Burial 4

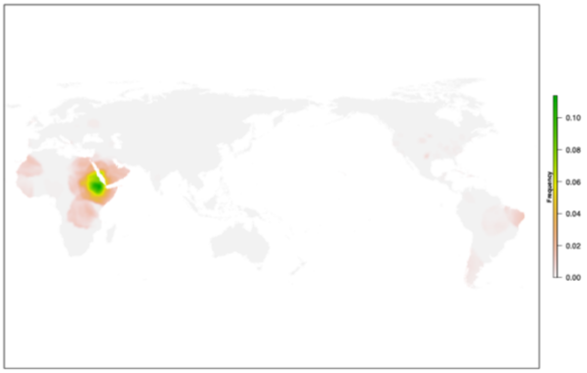

Haplogroup E1b1a1a1a
Burial 8 (E1b1a1a1a) & Burial 15 (E1b1a1a1a2a1a)

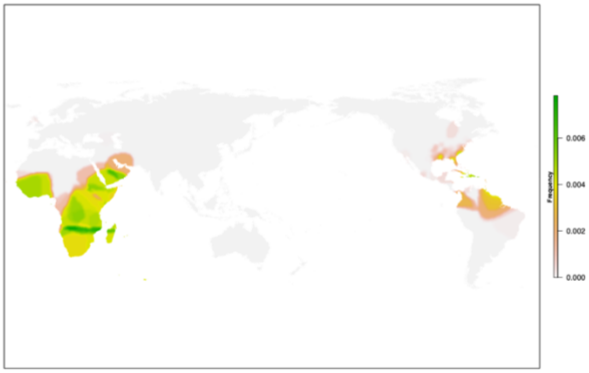

Haplogroup E1b1a1
Burial 26 (E1b1a1~)

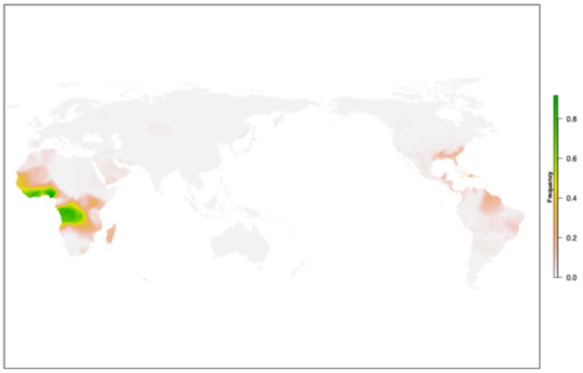

Haplogroup E1b1a1a1c1a
Burials 17 & 24 (Both E1b1a1a1a1c1a1)

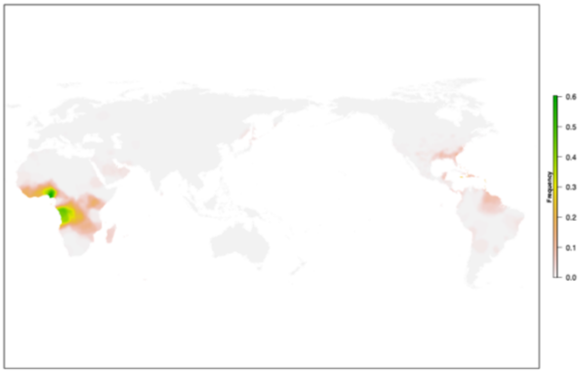

Haplogroup E1b1a1a1 (E-M180)
Burial 5

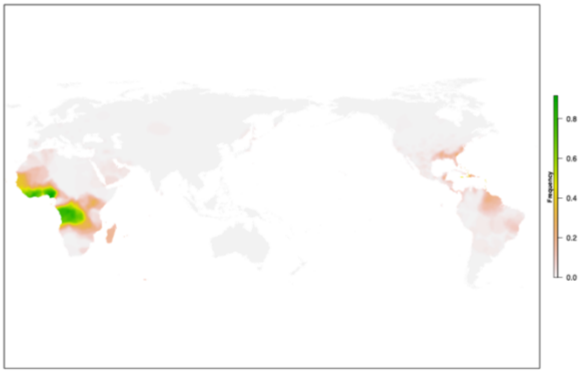

Haplogroup E1b1a1a1c1a1
Burial 22 (E1b1a1a1a1c1a1a3a1d1)

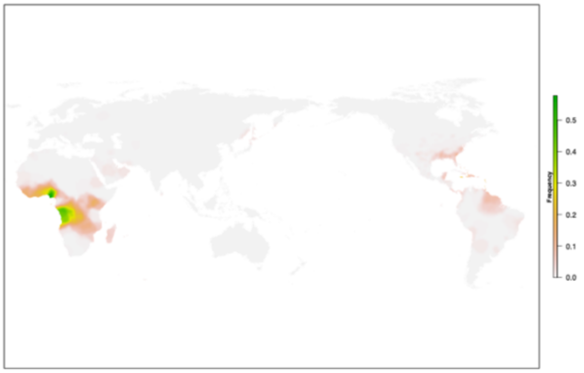

This is the author's version of the work. It is posted here by permission of the AAAS for personal use, not for redistribution. The definitive version was published in Science on August 4th, 2023, DOI: 10.1126/science.ade4995.

Haplogroup E1b1a1a1c1b
Burials 1, 2 (E1b1a1a1a1c1b1) & 6 (E1b1a1a1a1c1b2a)

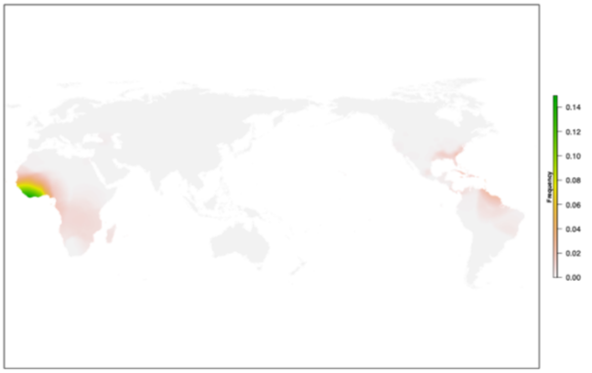

Haplogroup E1b1a1a1c2c
Burial 33

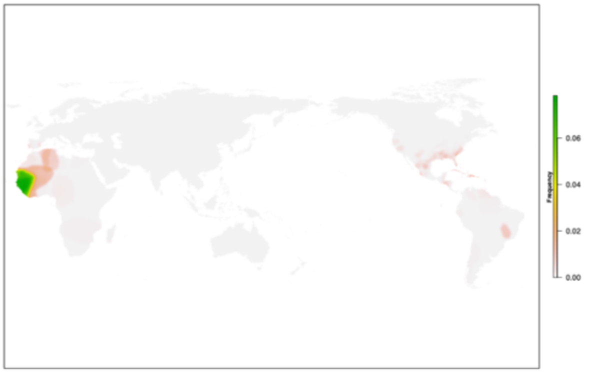

Haplogroup E2b
Burial 13

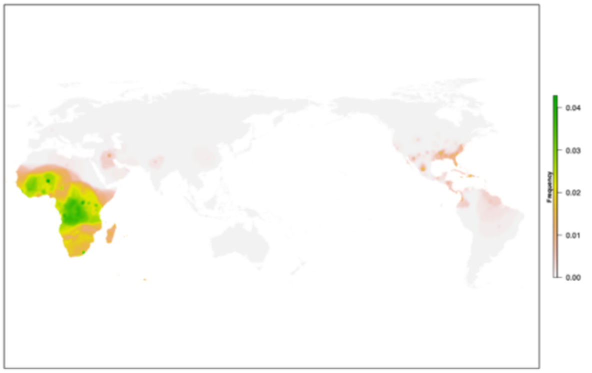

Haplogroup R1a1a1
Burial 32

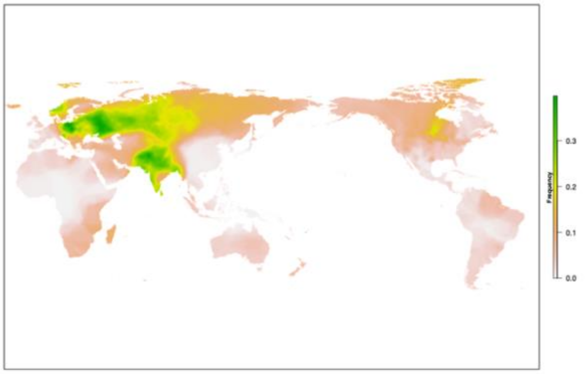

Haplogroup R1b1a
Burials 10 (R1b1a1b1a1a2c1a1h2a~) & 34 (R1b1a1a2a1a2c1)

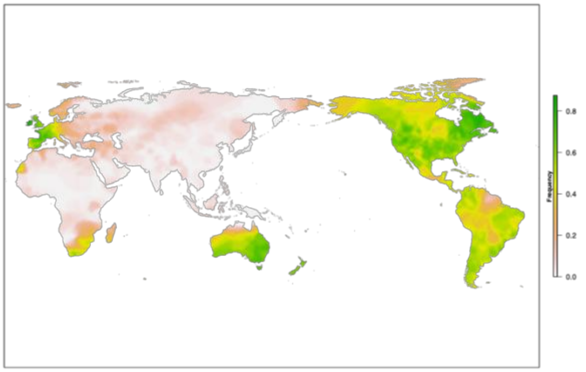

Fig. S1. Geographic distribution of the observed Y haplogroup frequencies in the 23andMe database using Kriging interpolation. For each haplogroup, the Catoctin individuals who have been assigned that haplogroup are indicated. In cases where the Catoctin individuals have been assigned a haplogroup that is a subclade of the displayed haplogroup, the full assigned haplogroup is reported in parentheses.

This is the author's version of the work. It is posted here by permission of the AAAS for personal use, not for redistribution. The definitive version was published in Science on August 4th, 2023, DOI: 10.1126/science.ade4995.

mt-Haplogroup Distributions

Haplogroup J1b1a
Burial 32 (J1b1a1a)

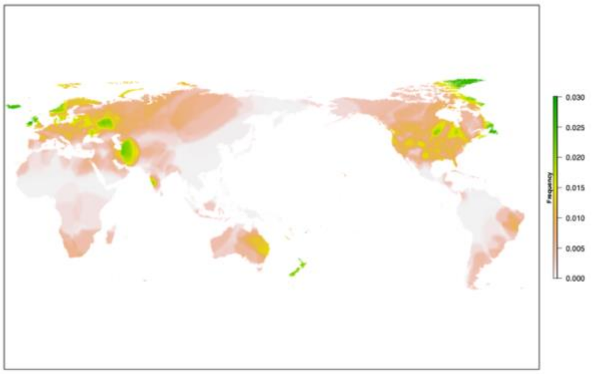

Haplogroup L2a1a1
Burial 6

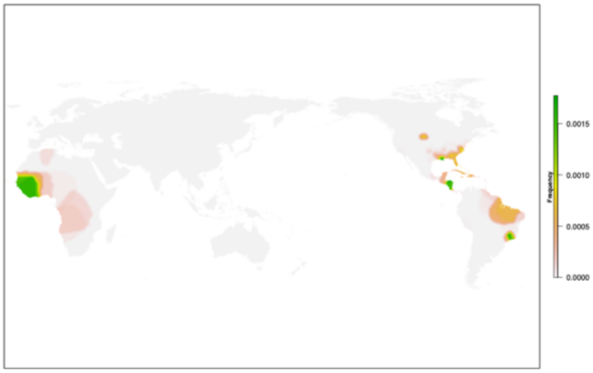

Haplogroup L0a1b1a
Burial 4

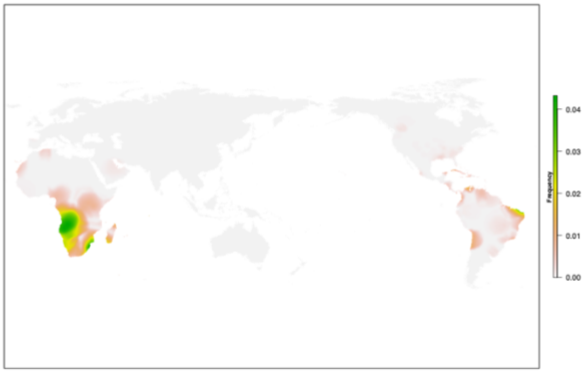

Haplogroup L2b1a3
Burials 11, 12, 13, & 28

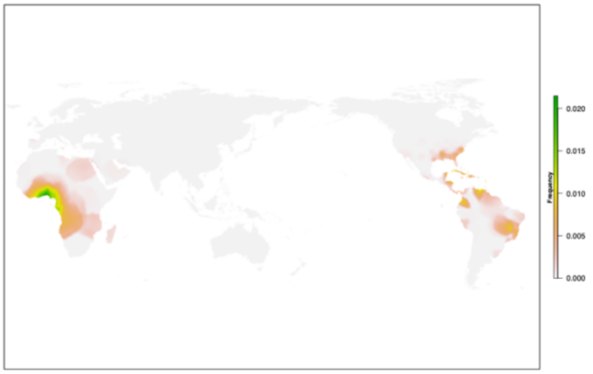

Haplogroup L2a1
Burials 15, 18, 23 (all L2a1+143+16189 (16192)+@16309) & 33 (L2a1+143+@16309)

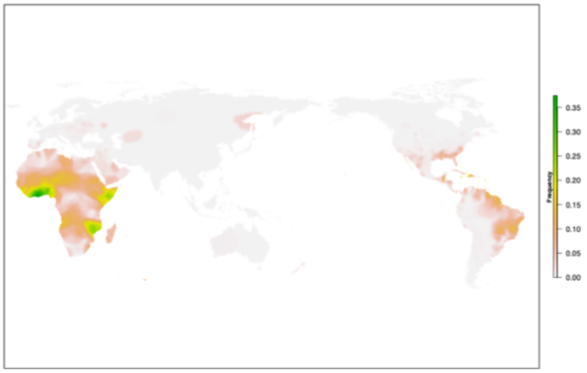

Haplogroup L2c
Burial 26

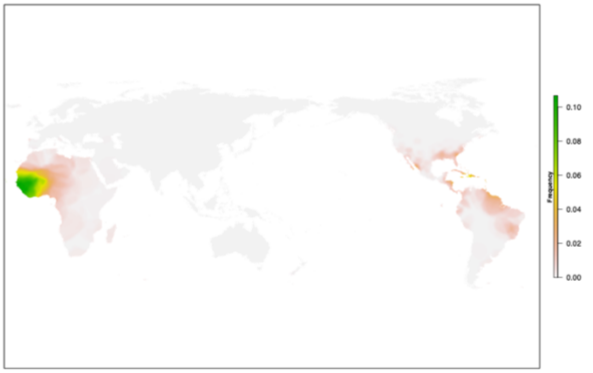

This is the author's version of the work. It is posted here by permission of the AAAS for personal use, not for redistribution. The definitive version was published in Science on August 4th, 2023, DOI: 10.1126/science.ade4995.

Haplogroup L3d1b3
Burial 22

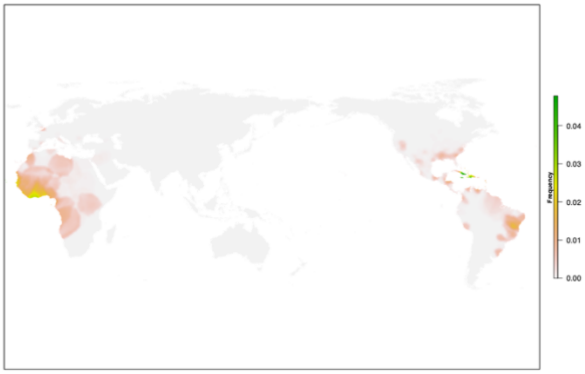

Haplogroup L3e2
Burials 17 & 19

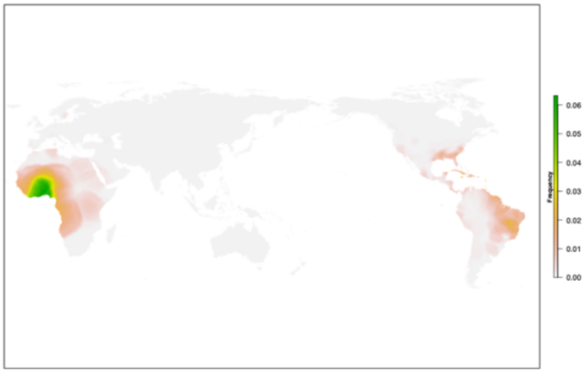

Haplogroup L3e1
Burials 5, 34, and 35

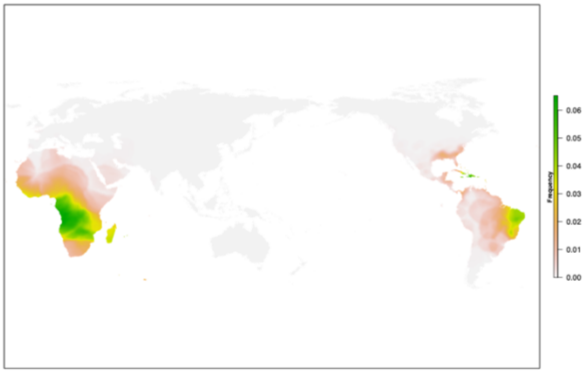

Haplogroup L3e2a1b1
Burials 1, 2, 3, 8 and 24

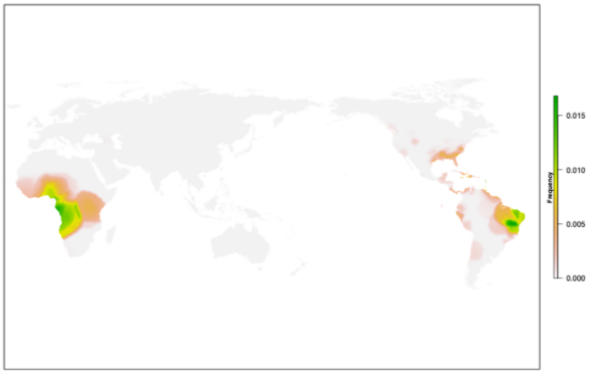

Haplogroup L3e1a1a
Burial 7

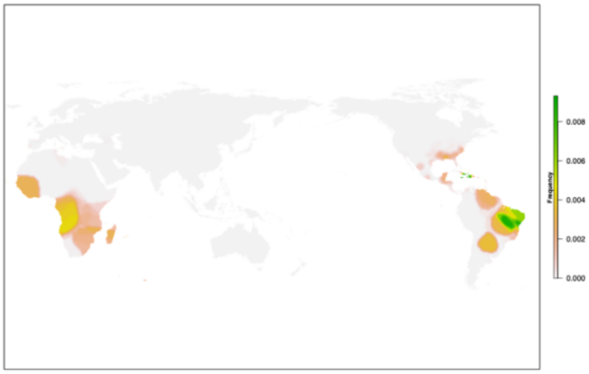

Haplogroup L3f1b1
Burial 9 (L3f1b1a)

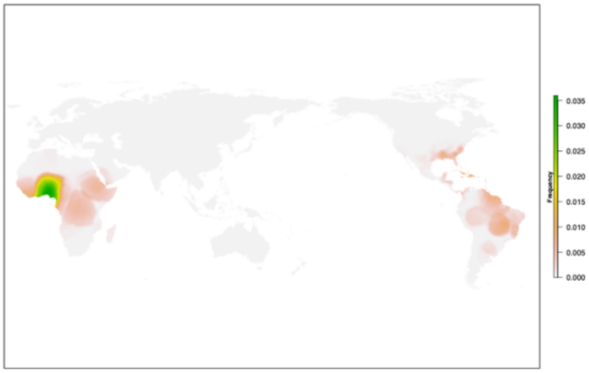

This is the author's version of the work. It is posted here by permission of the AAAS for personal use, not for redistribution. The definitive version was published in Science on August 4th, 2023, DOI: 10.1126/science.ade4995.

Haplogroup L3f1b3
Burial 10

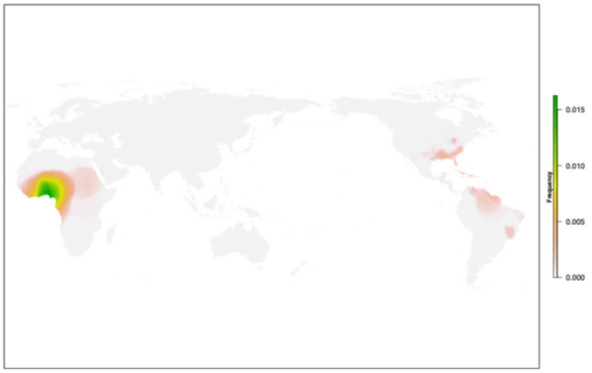

Haplogroup L4b2b1
Burial 14

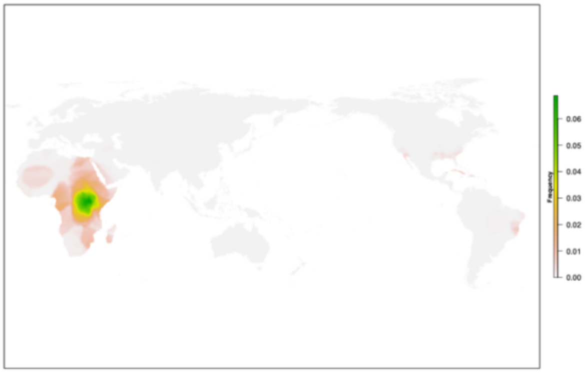

Fig. S2. Geographic distribution of the observed mt haplogroup frequencies in the 23andMe database using Kriging interpolation. For each haplogroup, the Catoctin individuals who have been assigned that haplogroup are indicated. In cases where the Catoctin individuals have been assigned a haplogroup that is a subclade of the displayed haplogroup, the full assigned haplogroup is reported in parentheses.

This is the author's version of the work. It is posted here by permission of the AAAS for personal use, not for redistribution. The definitive version was published in Science on August 4th, 2023, DOI: 10.1126/science.ade4995.

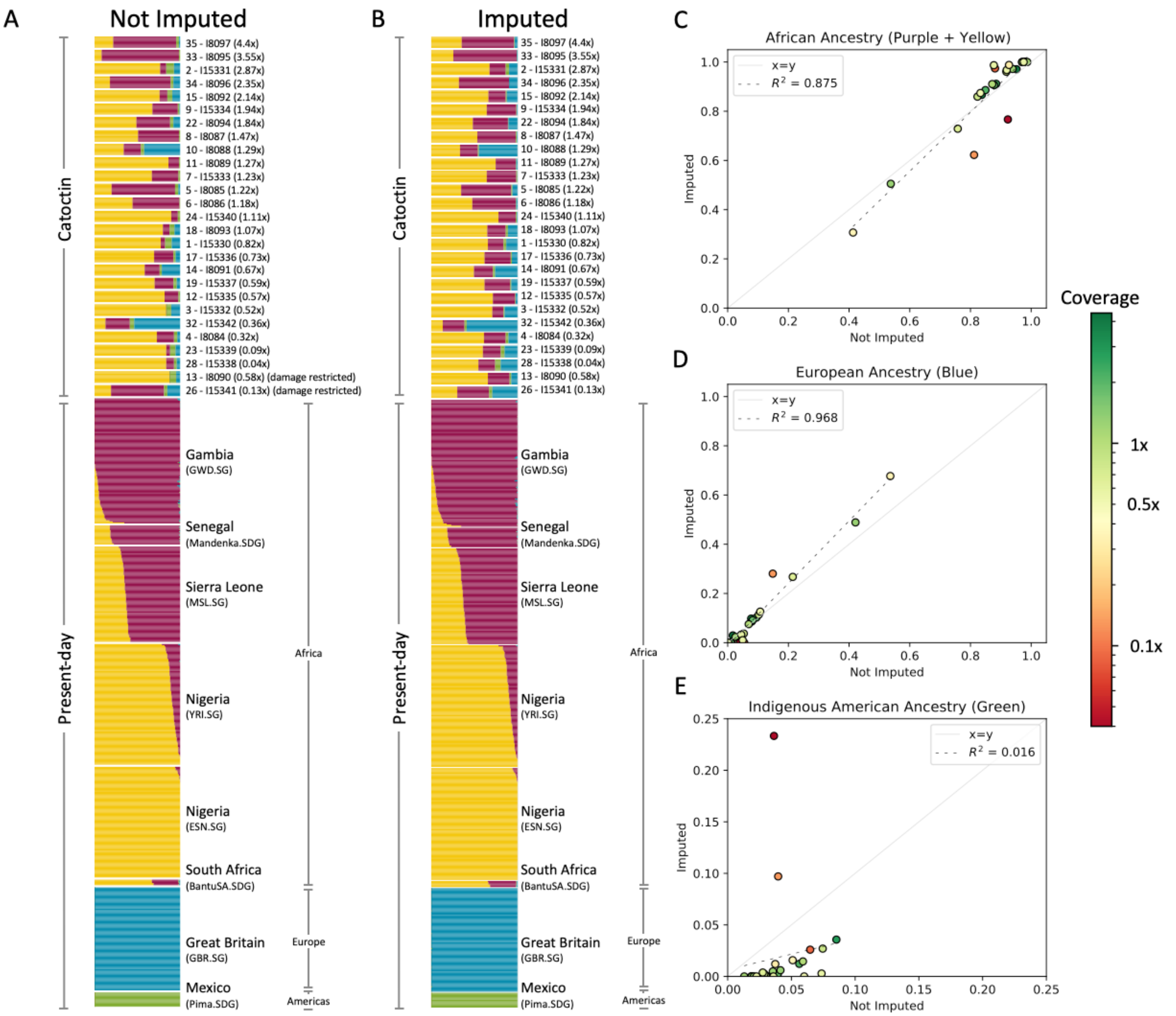

Fig. S3. ADMIXTURE analysis of 531 present-day individuals drawn from the public dataset from Africa (GWD.SG, Mandenka.SDG, MSL.SG, YRI.SG, ESN.SG and BantuSA.SDG), Europe (GBR.SD) and the Americas (Pima.SDG) and not imputed (a) or imputed (b) versions of the 27 Catoctin individuals with K = 4 ancestral components. The Catoctin individuals are ordered from highest to lowest coverage, placing the two individuals who are damage restricted in the non-imputed dataset last. Scatterplots demonstrate the difference in the amount of ancestry assigned to African (c), European (d) and Indigenous American (e) sources when calculated using the not imputed versus imputed datasets. The biggest deviations from a 1:1 ratio (x=y) occur among the lowest coverage individuals (shown in red) for all cases, particularly those with <0.5x coverage.

This is the author's version of the work. It is posted here by permission of the AAAS for personal use, not for redistribution. The definitive version was published in Science on August 4th, 2023, DOI: 10.1126/science.ade4995.

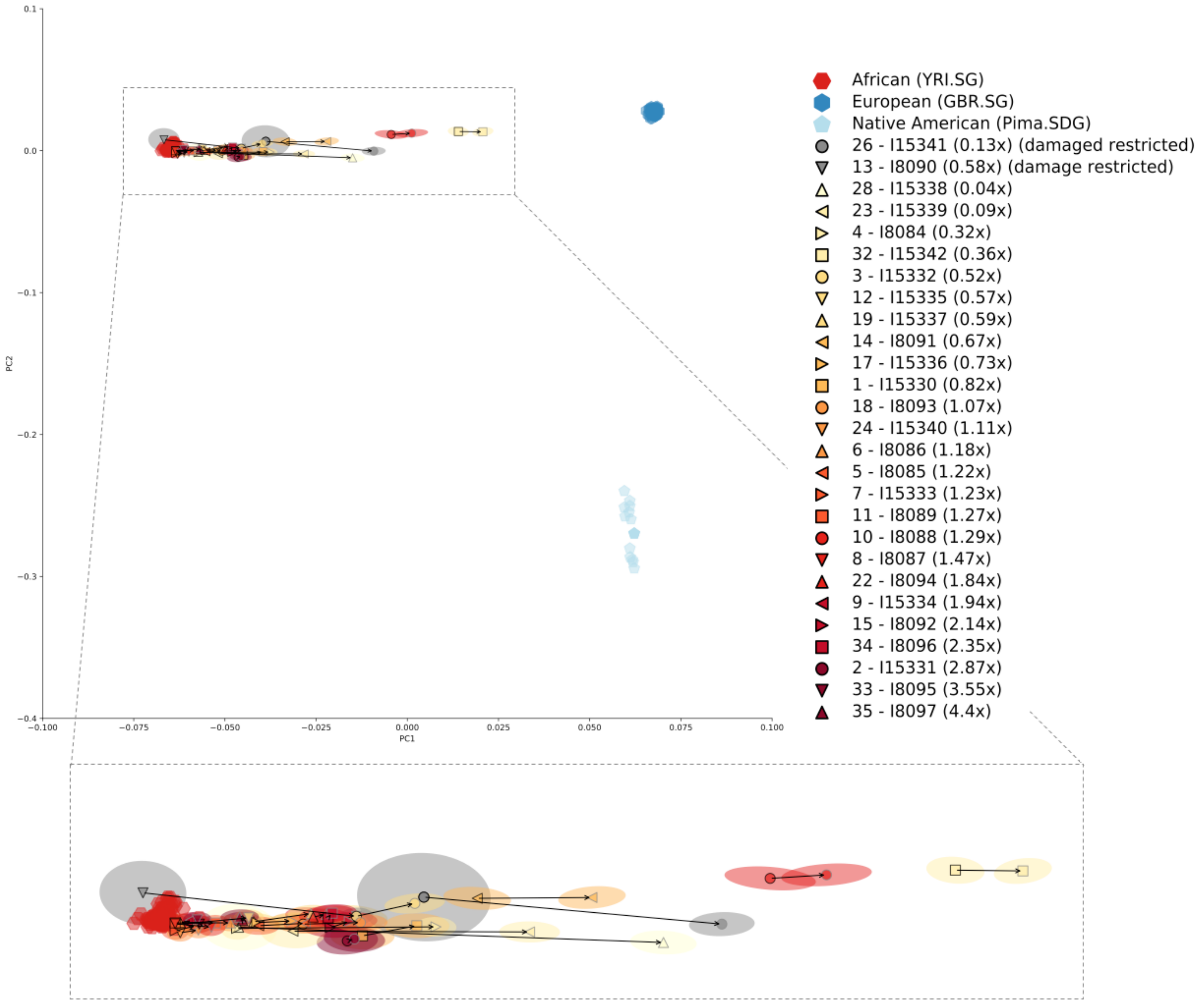

Fig. S4. African American Ancestry PCA. A principal component analysis of 213 present-day individuals from representative European (GBR.SG), African (YRI.SG) and Indigenous American (Pima.SDG) populations drawn from the public dataset. We projected the 27 Catoctin individuals onto the resulting PCA plot using non-imputed (black outline) and imputed (gray outline) versions of the Catoctin datasets. For each individual, a black arrow shows the change in position between the non-imputed and imputed versions of the data (pointing from the non-imputed data point towards the imputed data point). Ovals around each marker show the 95% confidence interval for the position of the marker. A zoomed-in view of the portion of the figure indicated with dotted lines is shown at the bottom of the figure. The position of the lowest

This is the author's version of the work. It is posted here by permission of the AAAS for personal use, not for redistribution. The definitive version was published in Science on August 4th, 2023, DOI: 10.1126/science.ade4995.

coverage and/or damage restricted Catoctin individuals changed the most dramatically between the non-imputed and imputed datasets.

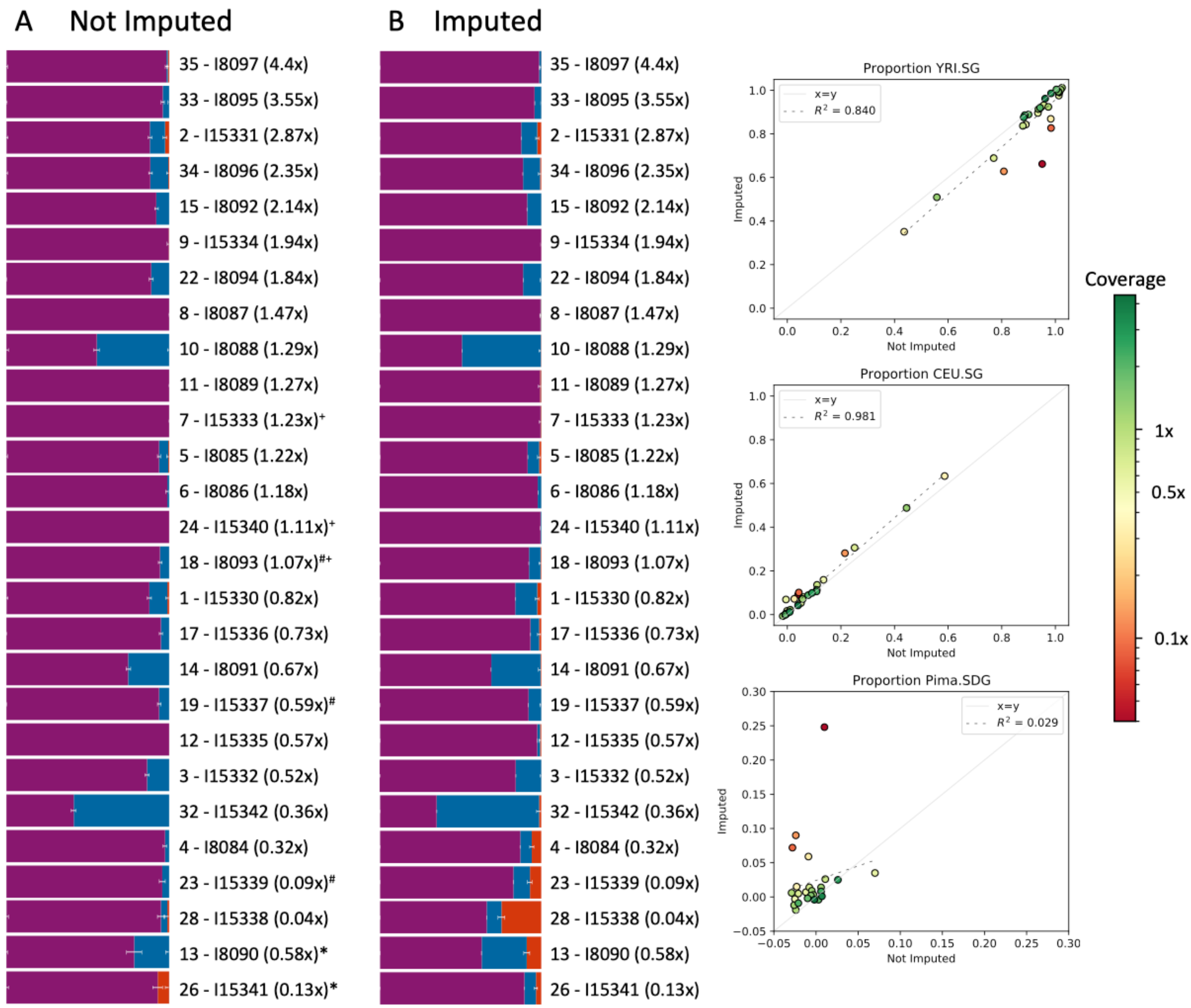

Fig. S5. qpADM estimates of African, European and Indigenous American Ancestry Proportions. The proportion of ancestry assigned to each Catoctin individual from representative African (YRI.SG), European (GBR.SG) and Indigenous American (Pima.SDG) populations drawn from the public dataset by the tool qpAdm using the not imputed (a) and imputed (b) datasets. Error bars indicate 1 standard error. Hash symbols (#) indicate models with p-values <0.01 and plus signs (+) indicate models with ancestry proportion estimates that fall more than 3 standard errors outside the range of 0-1. Asterisks (*) indicate damage restricted data. In panels c-e, scatterplots demonstrate the difference in the amount of ancestry assigned to YRI.SG (c), CEU.SG (d) and Pima.SDG (e) sources when calculated using the not imputed versus imputed datasets. The biggest deviations from a 1:1 ratio (x=y) occur among the lowest coverage individuals (shown in red) for all cases, particularly those with <0.5x coverage.

This is the author's version of the work. It is posted here by permission of the AAAS for personal use, not for redistribution. The definitive version was published in Science on August 4th, 2023, DOI: 10.1126/science.ade4995.

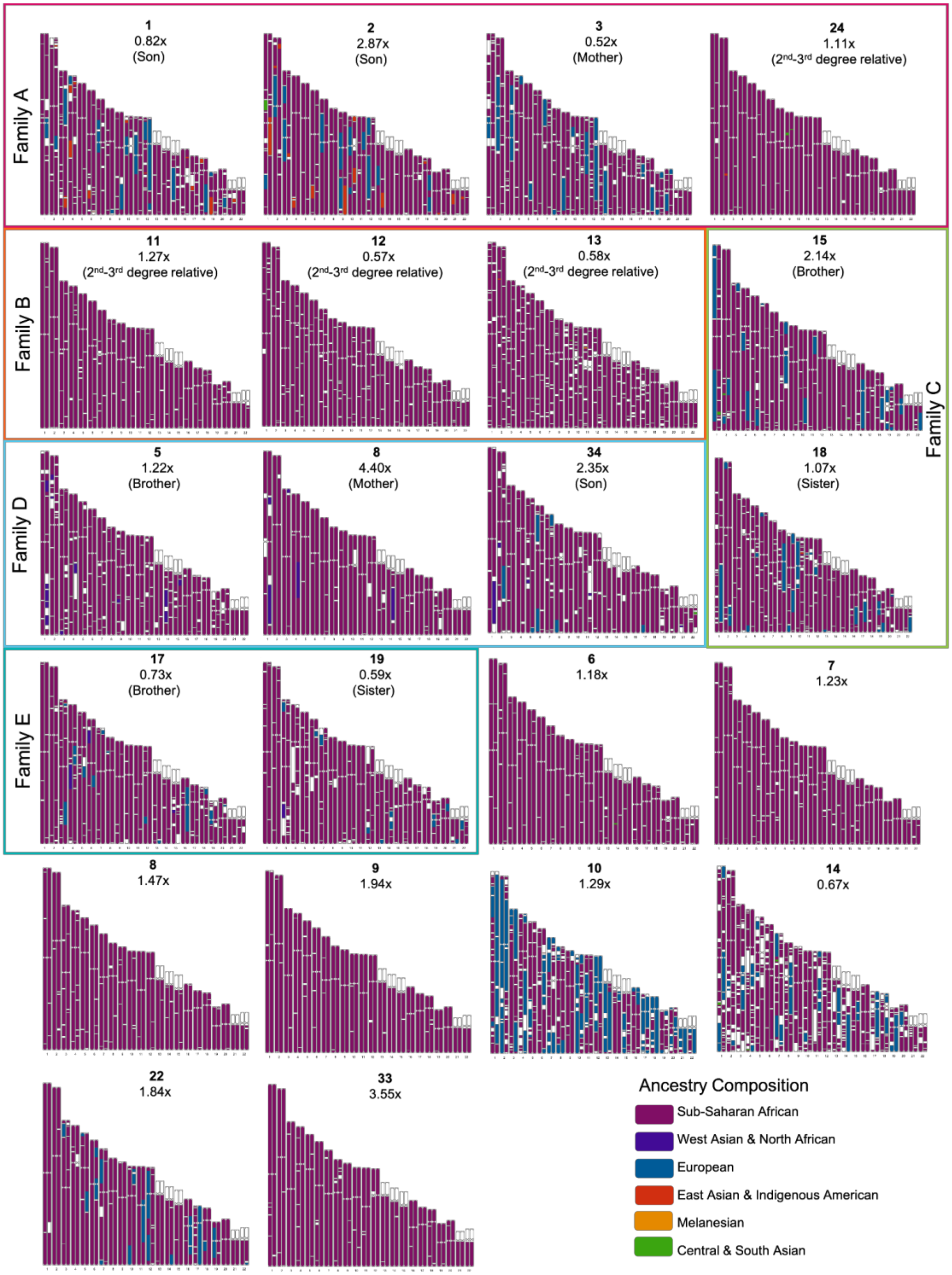

Fig. S6. Chromosome paintings showing the ancestry assigned to portions of the autosomal chromosomes for the 22 Catoctin individuals with >0.5x coverage. Catoctin individuals are grouped together into their assigned genetic families when possible. Across the genome, ancestry is assigned to one of six ancestry components defined using 23andMe reference populations: Sub-Saharan African (purple), West Asian & North African (dark blue), European (dark teal), East Asian & Indigenous American (red), Melanesian (orange), and Central & South Asian (green). Portions of the genome that could not be assigned to any of these components are shown in white.

This is the author's version of the work. It is posted here by permission of the AAAS for personal use, not for redistribution. The definitive version was published in Science on August 4th, 2023, DOI: 10.1126/science.ade4995.

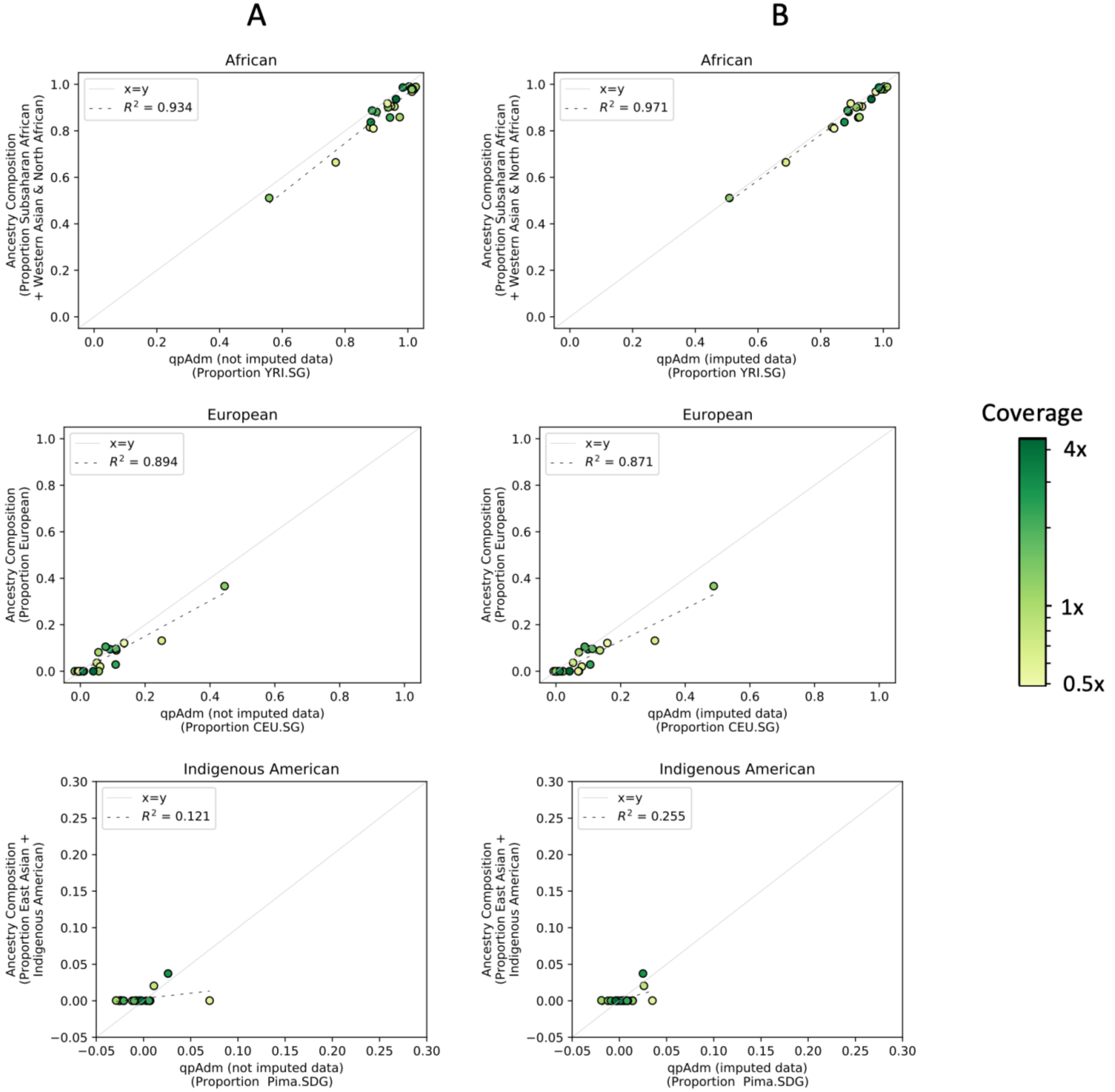

Fig. S7. Correlation between Ancestry Composition and qpAdm assignments. Plots showing the proportion of ancestry assigned to African (top), European (middle) and Indigenous American (bottom) sources by qpAdm (using the public dataset) and Ancestry Composition (using 23andMe reference populations) for each Catoctin individual (blue dots) with >0.5x coverage when calculated using the not imputed (a) and imputed (b) datasets.

This is the author's version of the work. It is posted here by permission of the AAAS for personal use, not for redistribution. The definitive version was published in Science on August 4th, 2023, DOI: 10.1126/science.ade4995.

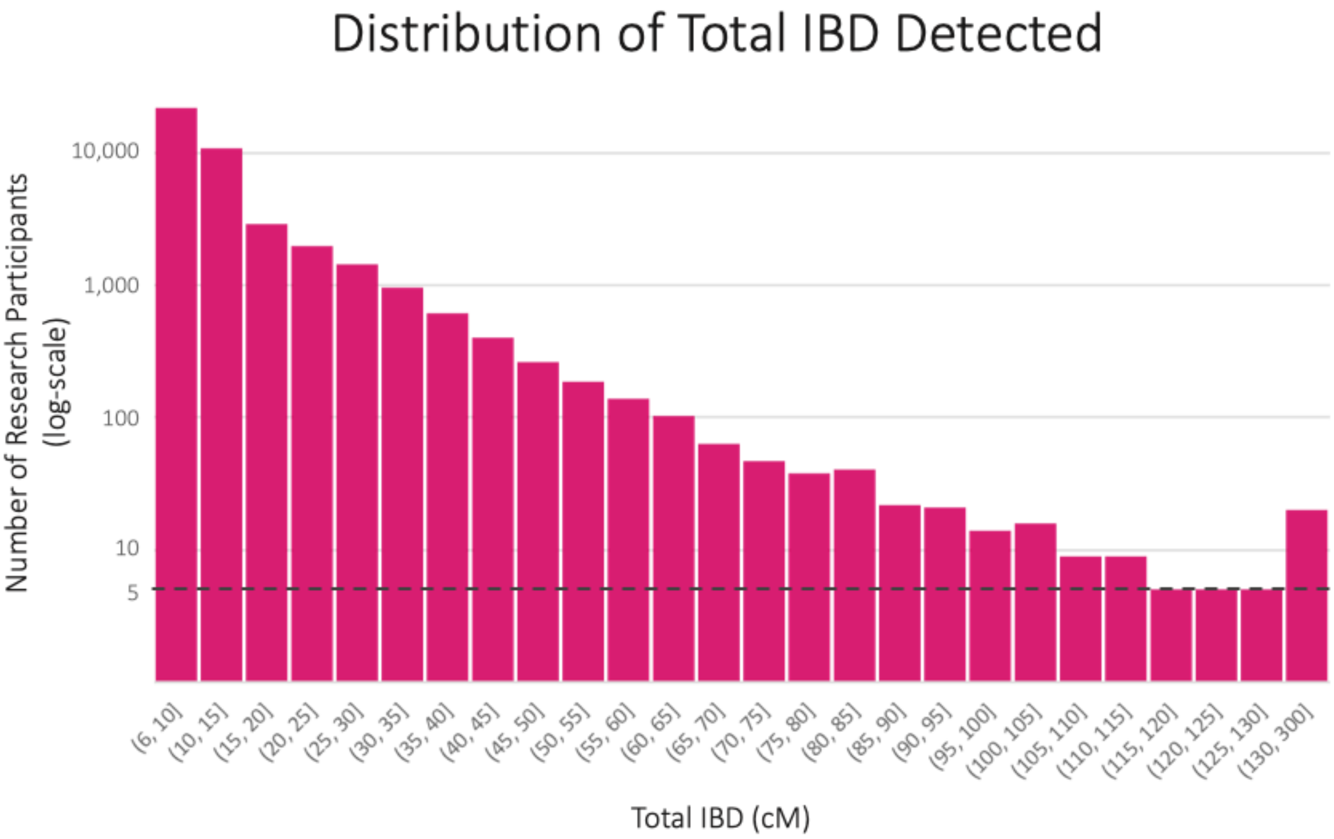

Fig. S8. Histogram of total IBD detected among 23andMe research participants who share IBD with one or more Catoctin individuals. For research participants who share IBD with multiple Catoctin individuals, the larger amount of total IBD is reported. Bin sizes were selected to include a minimum of 5 research participants.

This is the author's version of the work. It is posted here by permission of the AAAS for personal use, not for redistribution. The definitive version was published in Science on August 4th, 2023, DOI: 10.1126/science.ade4995.

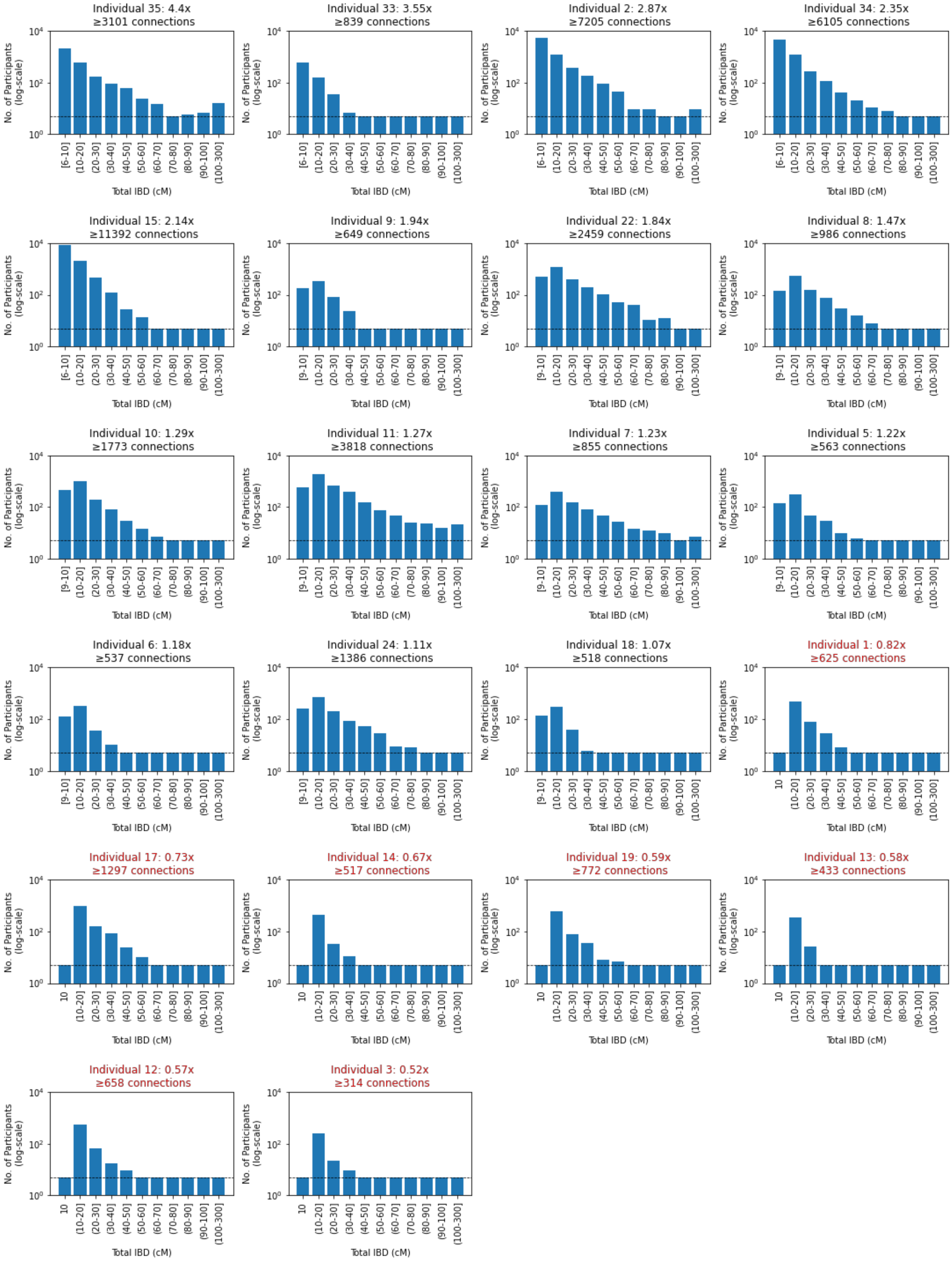

Fig. S9. Histogram of total IBD shared with each Catoctin individual. Bins with ≤5 associated 23andMe research participants are reported as 5, indicated by the black, dotted horizontal line. A minimum segment length threshold was applied based on coverage using the following thresholds: ≥2x: 6cM, 1-2x: 9 cM, 0.5-1x: 10 cM. Catoctin individuals with <1x coverage are labeled in red to indicate that these results should be interpreted with caution. The total number of connections shared with each Catoctin individual is indicated above each plot, using the same masking approach for bins with ≥5 connections.

This is the author's version of the work. It is posted here by permission of the AAAS for personal use, not for redistribution. The definitive version was published in Science on August 4th, 2023, DOI: 10.1126/science.ade4995.

Burial 35
5 Connections

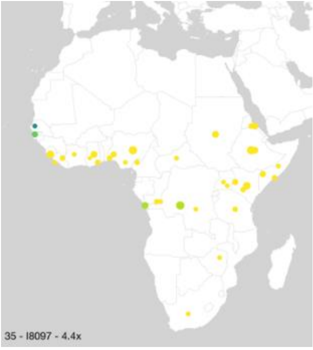

Burial 33
0 Connections

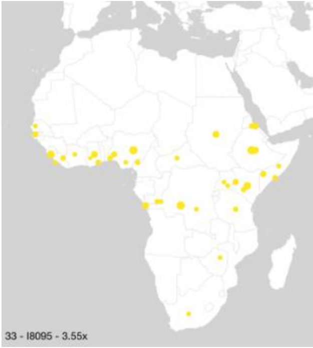

Burial 2
15 Connections

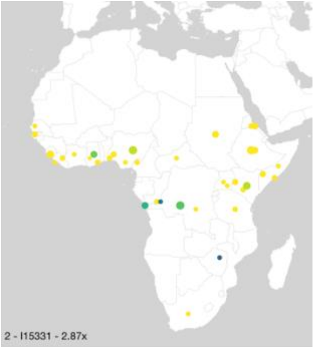

Burial 34
16 Connections

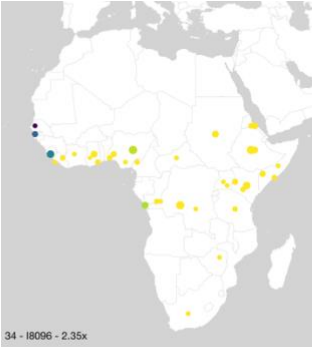

Burial 15
2 Connections

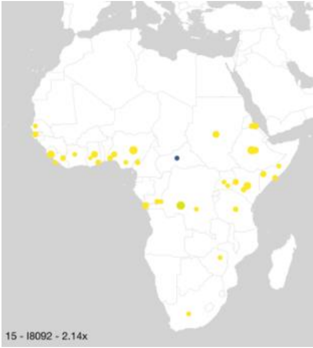

Burial 9
1 Connection

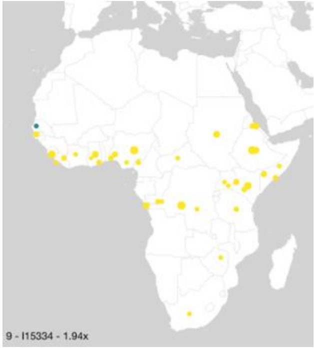

Burial 22
2 Connections

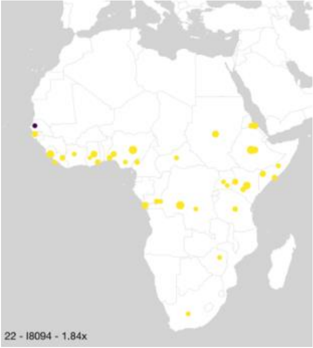

Burial 8
0 Connections

Burial 10
0 Connections

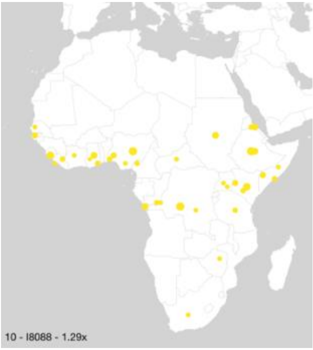

This is the author's version of the work. It is posted here by permission of the AAAS for personal use, not for redistribution. The definitive version was published in Science on August 4th, 2023, DOI: 10.1126/science.ade4995.

Burial 11
1 Connection

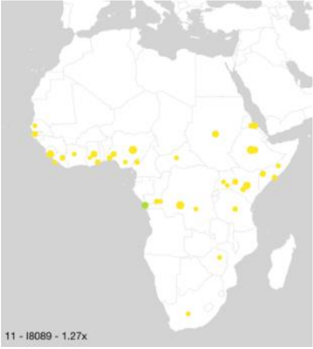

Burial 7
3 Connections

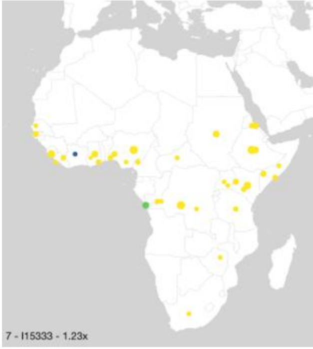

Burial 5
0 Connections

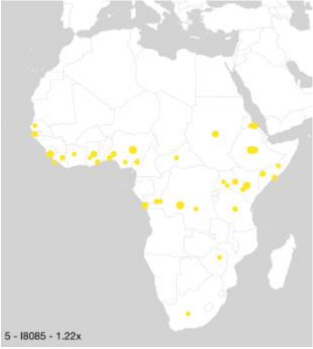

Burial 6
3 Connections

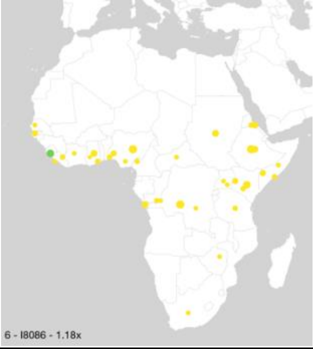

Burial 24
1 Connection

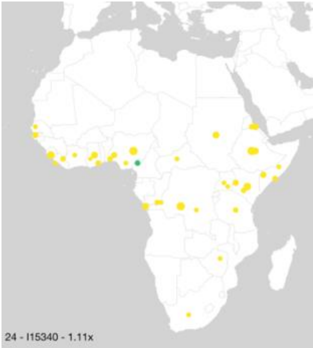

Burial 18
0 Connections

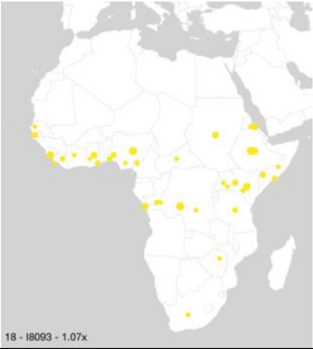

Burial 1
1 Connection

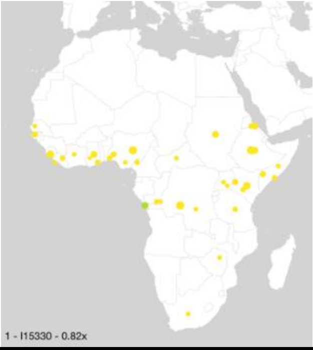

Burial 17
0 Connections

Burial 14
0 Connections

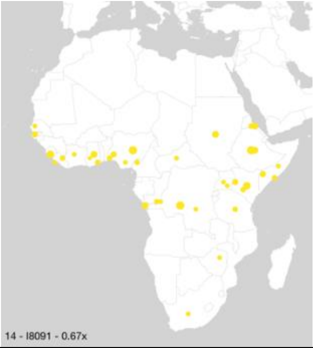

Burial 19
0 Connections

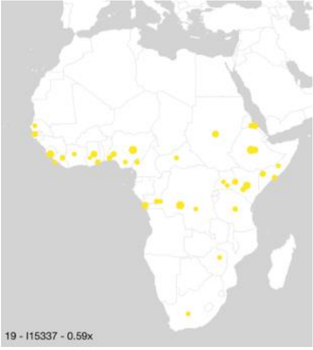

Burial 13
1 Connection

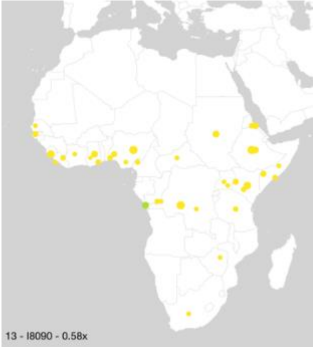

Burial 12
0 Connections

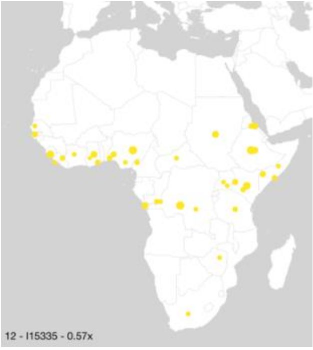

This is the author's version of the work. It is posted here by permission of the AAAS for personal use, not for redistribution. The definitive version was published in Science on August 4th, 2023, DOI: 10.1126/science.ade4995.

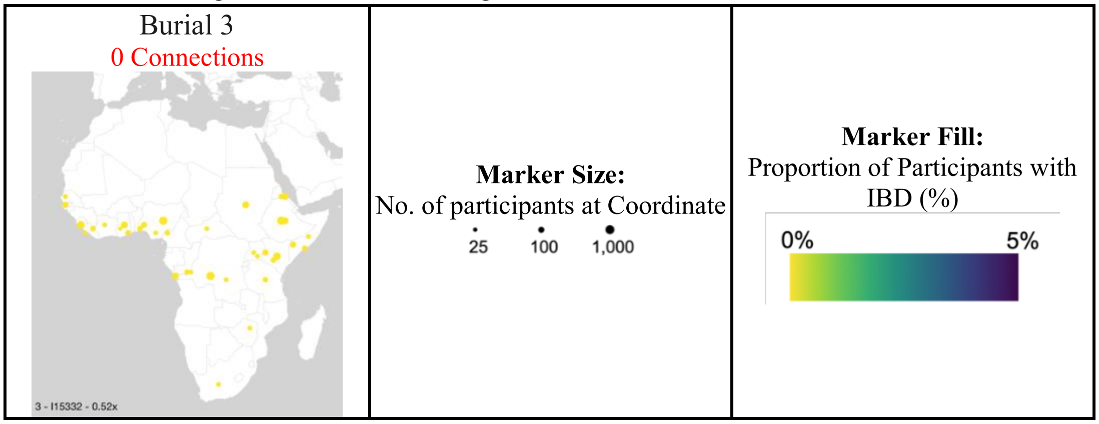

Fig. S10. Genetic connections to each of the Catoctin individuals among African participants. The proportion of 23andMe participants with ≥95% Sub-Saharan African ancestry in Africa who share IBD with each Catoctin individual with >0.5x coverage. Geographic coordinates are rounded to the nearest integer, and only coordinates that have at least 25 associated participants are shown. To further protect participant privacy, we randomly downsample to include results for only 80% of participants. The size of the marker corresponds to the number of participants associated with each site, while the color indicates the proportion of participants who share IBD with the Catoctin individuals. No participants share ≥30 cM of IBD with one or more Catoctin individuals, so no marker outlines are shown. The total number of IBD connections observed is reported above each image, when 0 connections are observed, this number is reported in red.

This is the author's version of the work. It is posted here by permission of the AAAS for personal use, not for redistribution. The definitive version was published in Science on August 4th, 2023, DOI: 10.1126/science.ade4995.

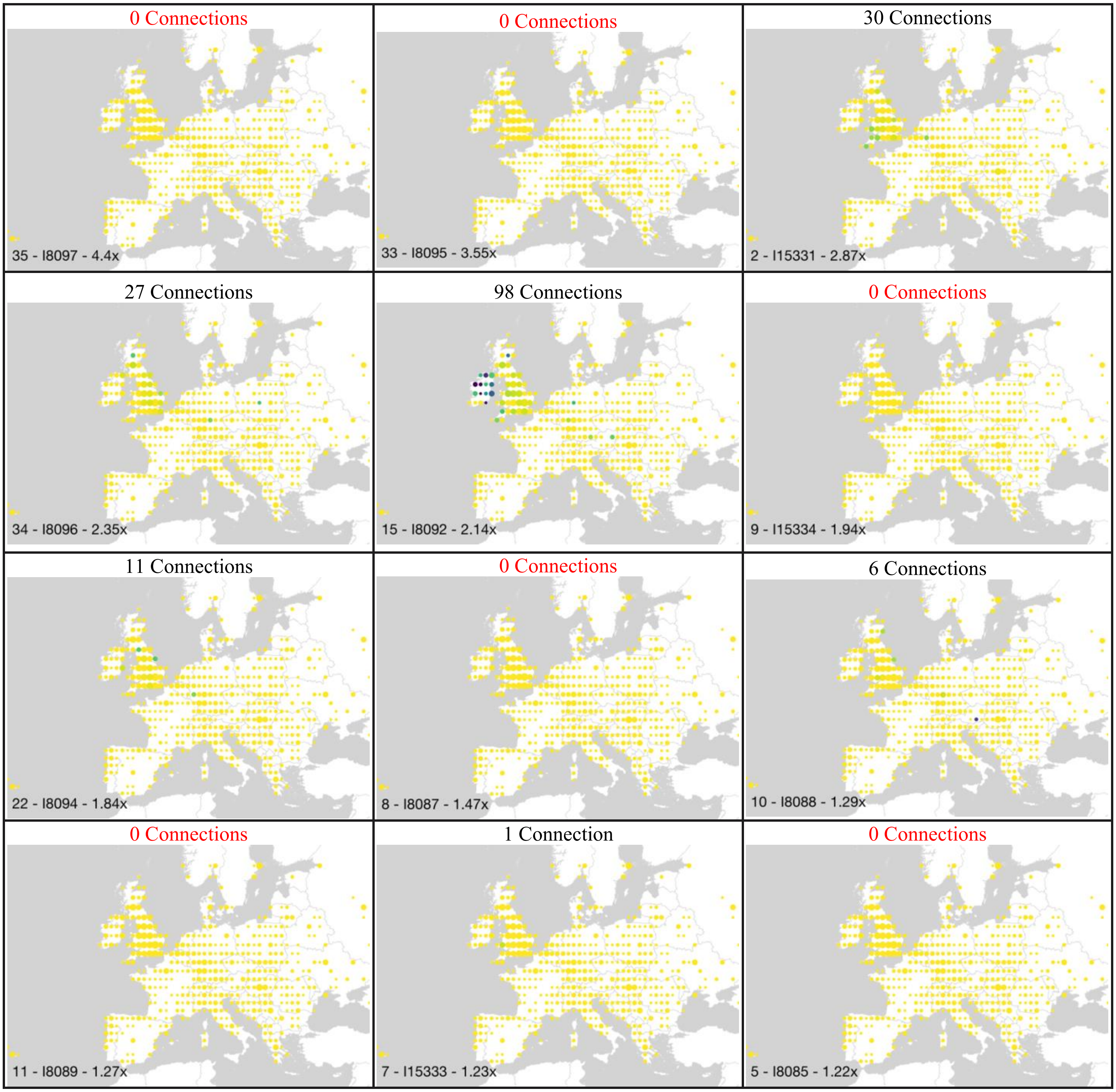

This is the author's version of the work. It is posted here by permission of the AAAS for personal use, not for redistribution. The definitive version was published in Science on August 4th, 2023, DOI: 10.1126/science.ade4995.

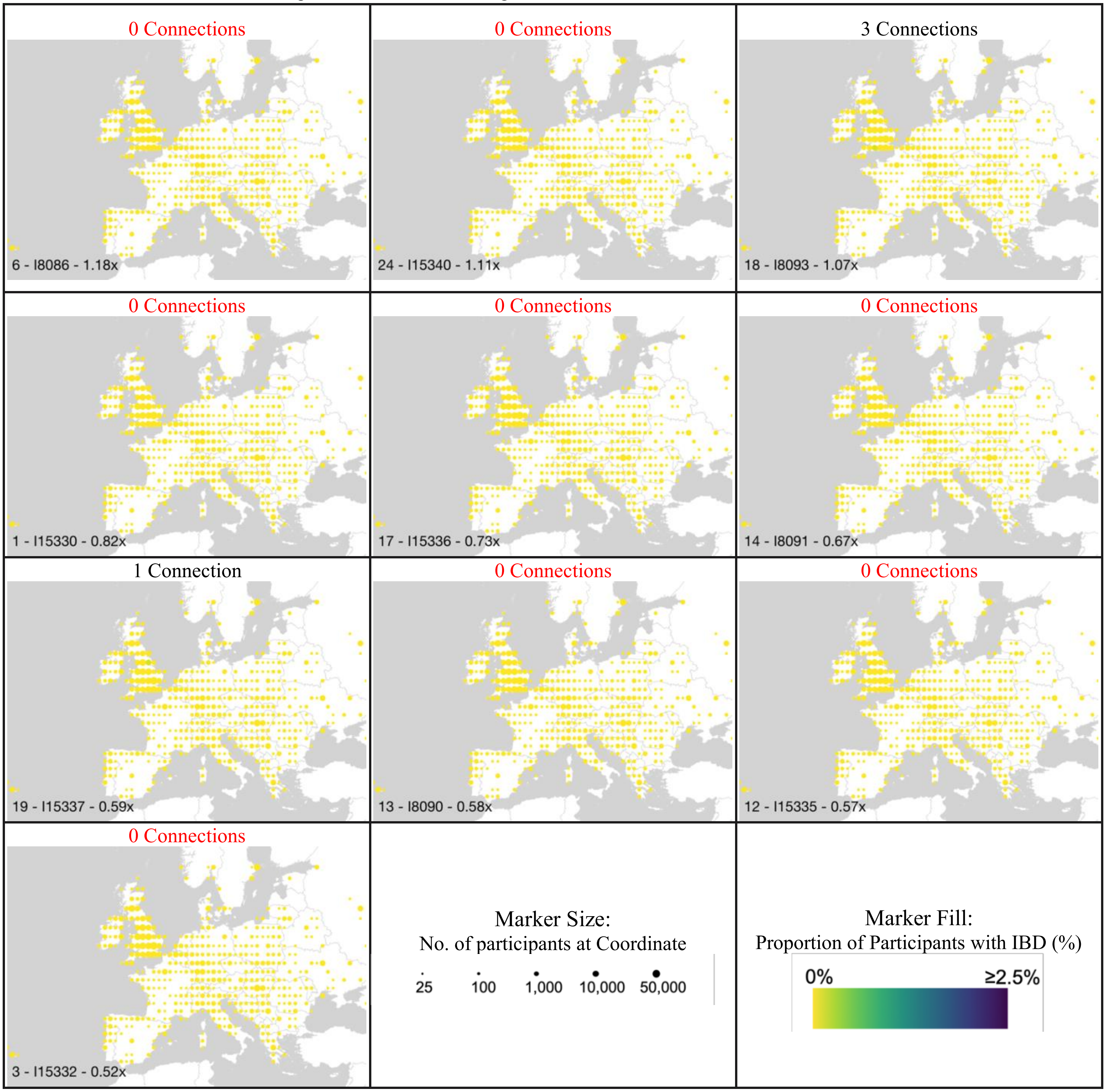

Fig. S11. Genetic connections to each of the Catoctin individuals among European participants. The proportion of 23andMe participants with ≥99% European ancestry in western and central Europe who share IBD with each Catoctin individual with >0.5x coverage. Geographic coordinates are rounded to the nearest integer, and only coordinates that have at least 25 associated participants are shown. To further protect participant privacy, we randomly downsample to include results for only 80% of participants. The size of the marker corresponds to the number of participants associated with each site, while the color indicates the proportion of participants who share IBD with the Catoctin individuals. No participants share ≥30 cM of IBD with one or more Catoctin individuals, so no marker outlines are shown. The total number of IBD connections observed is reported above each image, when 0 connections are observed, this number is reported in red.

This is the author's version of the work. It is posted here by permission of the AAAS for personal use, not for redistribution. The definitive version was published in Science on August 4th, 2023, DOI: 10.1126/science.ade4995.

**A.**<br>All US Participants	**B.**<br>US Participants with ≥5% Sub-Saharan African ancestry	**C.**<br>US participants with ≥ 99% European Ancestry
290 Connections<br>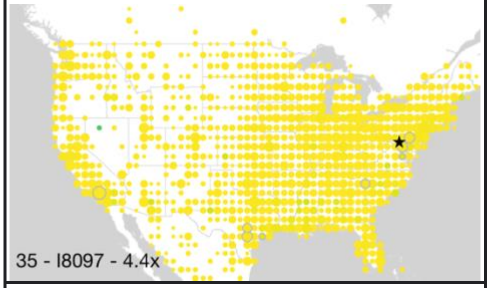<br>35 - I8097 - 4.4x	261 Connections<br>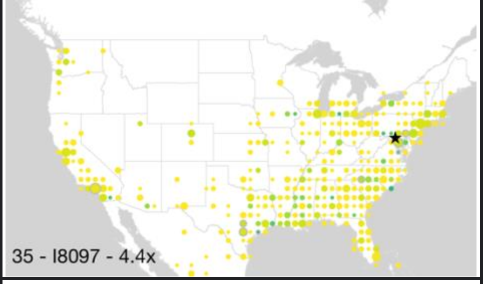<br>35 - I8097 - 4.4x	3 Connections<br>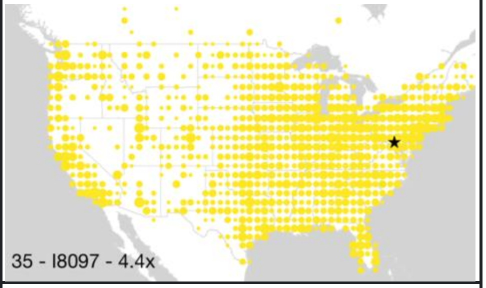<br>35 - I8097 - 4.4x
99 Connections<br>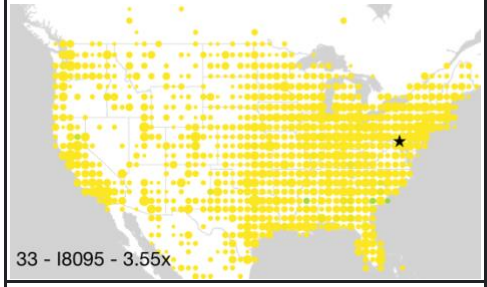<br>33 - I8095 - 3.55x	89 Connections<br>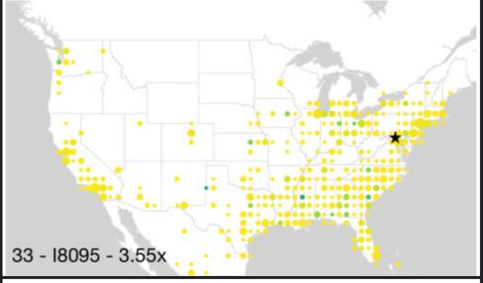<br>33 - I8095 - 3.55x	0 Connections<br>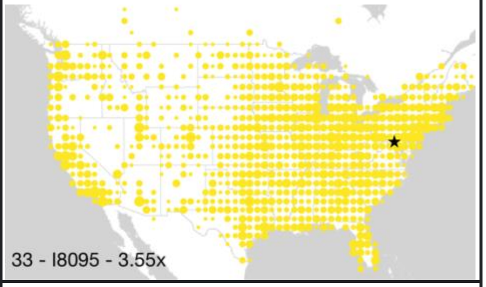<br>33 - I8095 - 3.55x
835 Connections<br>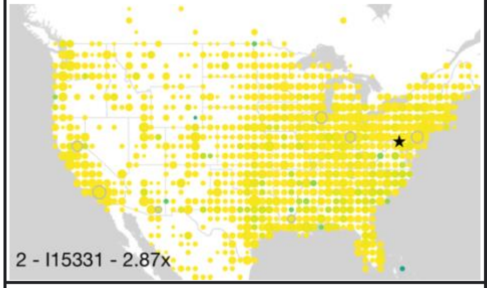<br>2 - I15331 - 2.87x	367 Connections<br>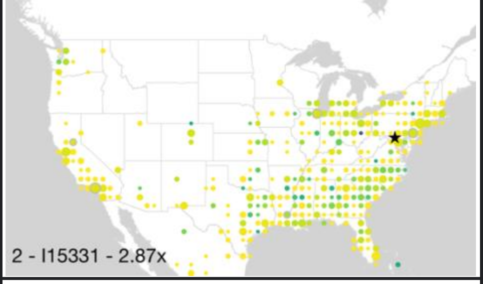<br>2 - I15331 - 2.87x	325 Connections<br>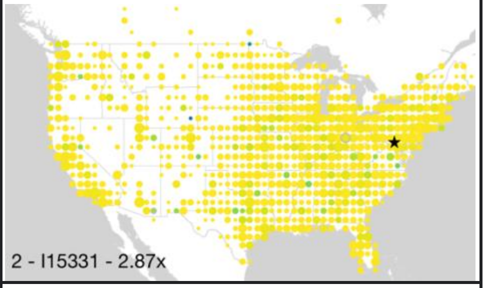<br>2 - I15331 - 2.87x
650 Connections<br>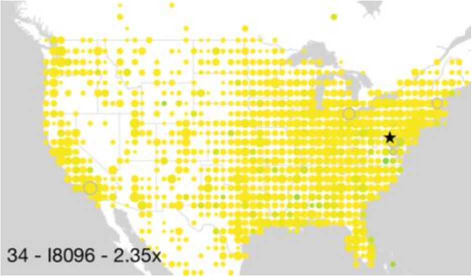<br>34 - I8096 - 2.35x	327 Connections<br>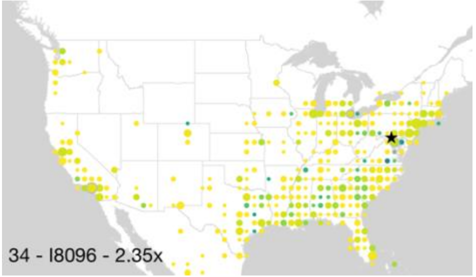<br>34 - I8096 - 2.35x	226 Connections<br>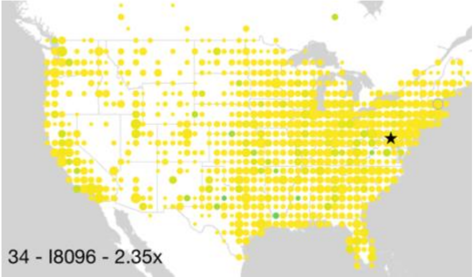<br>34 - I8096 - 2.35x

This is the author's version of the work. It is posted here by permission of the AAAS for personal use, not for redistribution. The definitive version was published in Science on August 4th, 2023, DOI: 10.1126/science.ade4995.

1,217 Connections	242 Connections	774 Connections
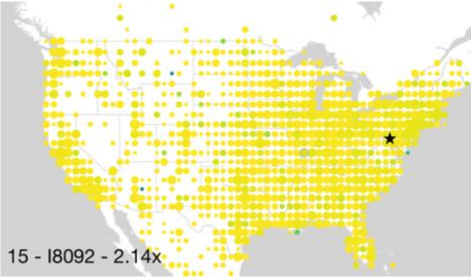	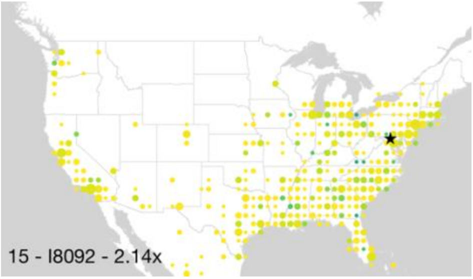	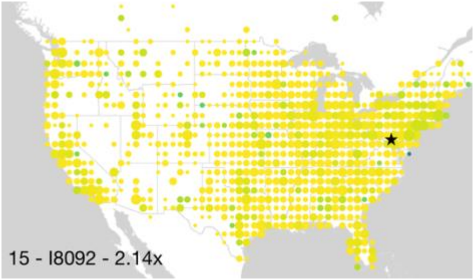
63 Connections	54 Connections	0 Connections
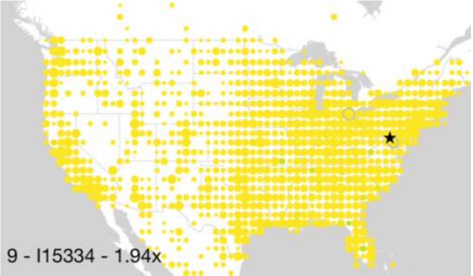	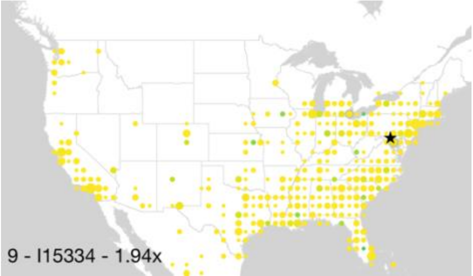	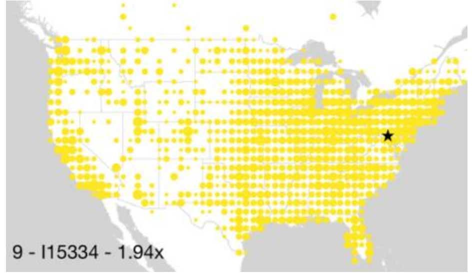
274 Connections	154 Connections	83 Connections
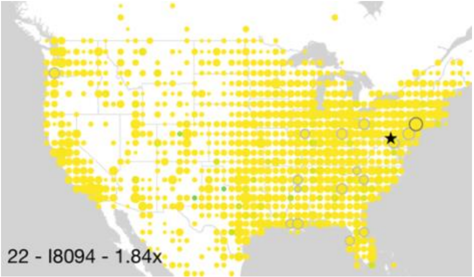	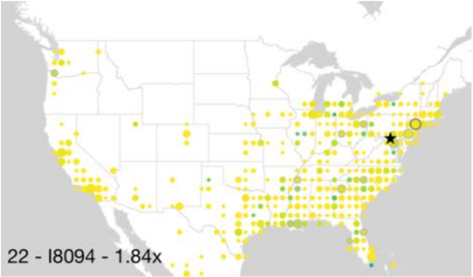	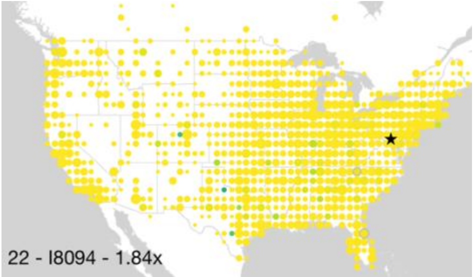
103 Connections	95 Connections	0 Connections
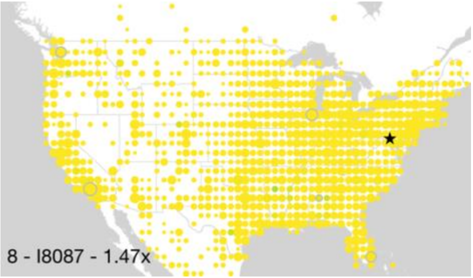	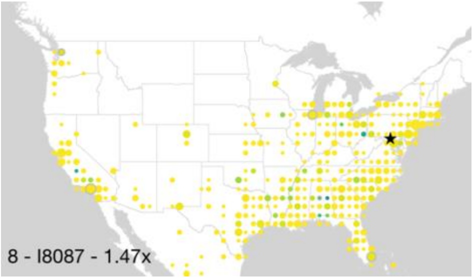	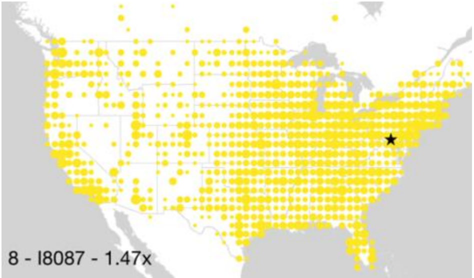
188 Connections	70 Connections	83 Connections
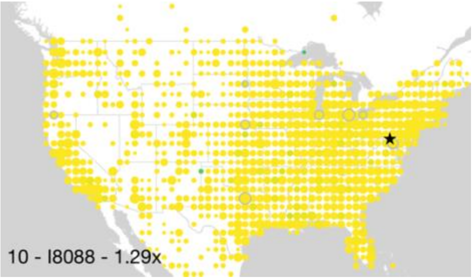	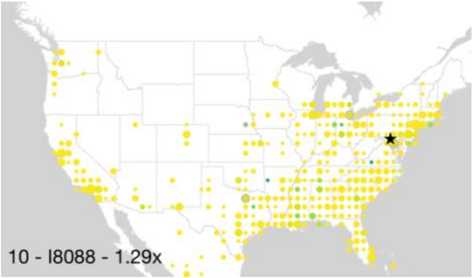	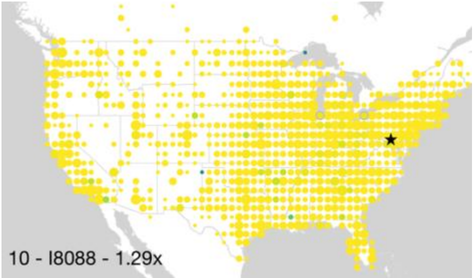
427 Connections	399 Connections	1 Connection

This is the author's version of the work. It is posted here by permission of the AAAS for personal use, not for redistribution. The definitive version was published in Science on August 4th, 2023, DOI: 10.1126/science.ade4995.

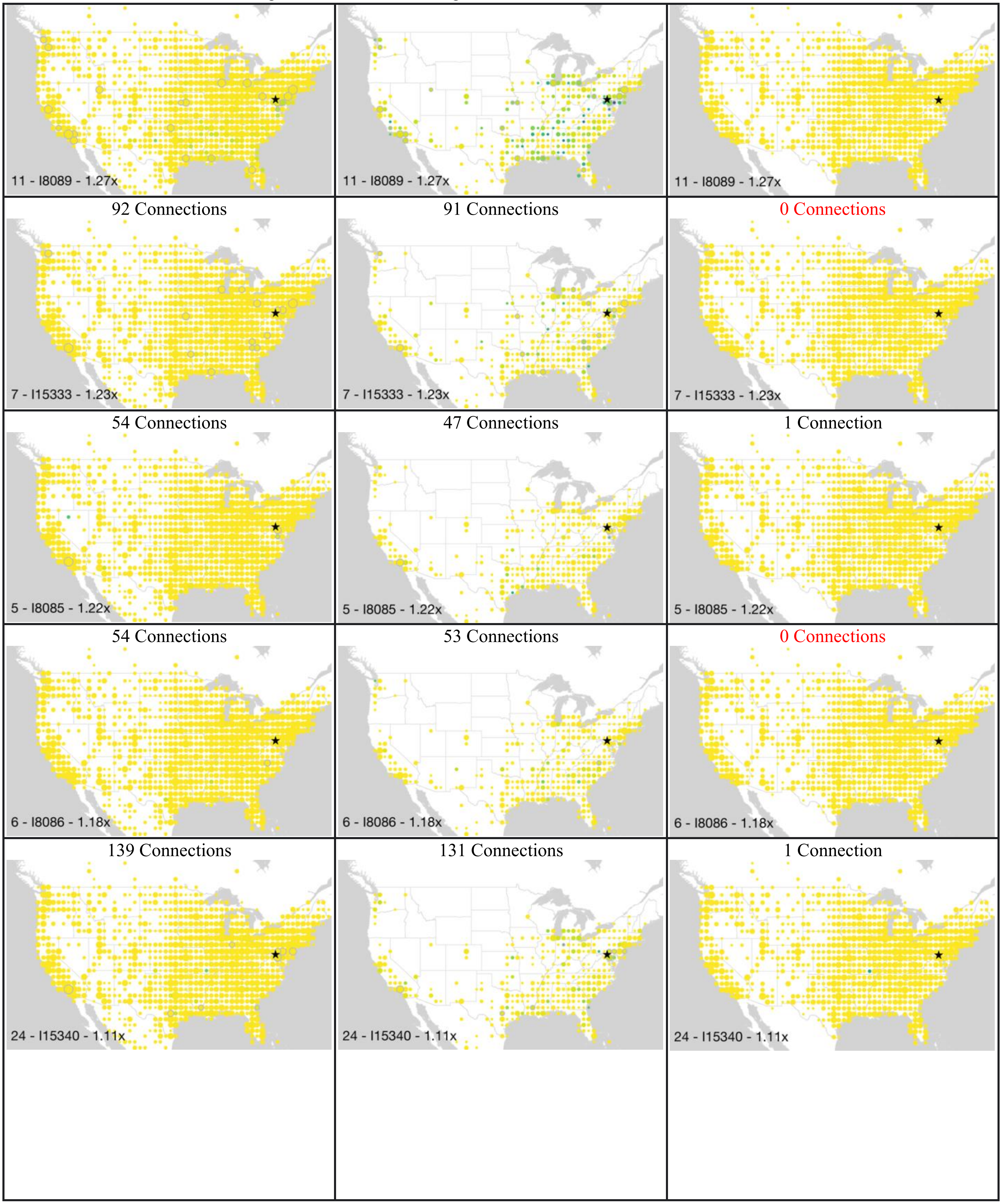

This is the author's version of the work. It is posted here by permission of the AAAS for personal use, not for redistribution. The definitive version was published in Science on August 4th, 2023, DOI: 10.1126/science.ade4995.

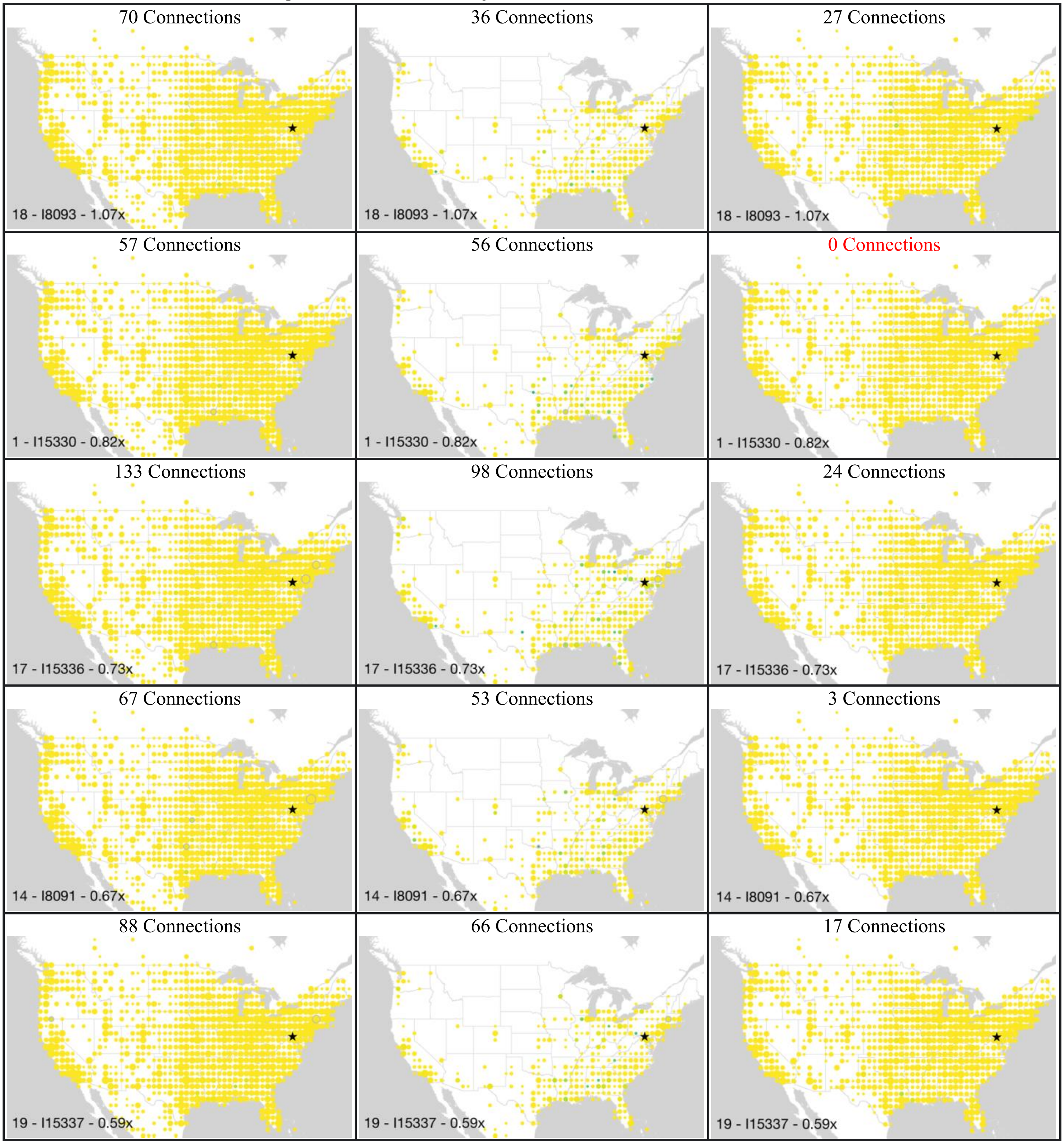

This is the author's version of the work. It is posted here by permission of the AAAS for personal use, not for redistribution. The definitive version was published in Science on August 4th, 2023, DOI: 10.1126/science.ade4995.

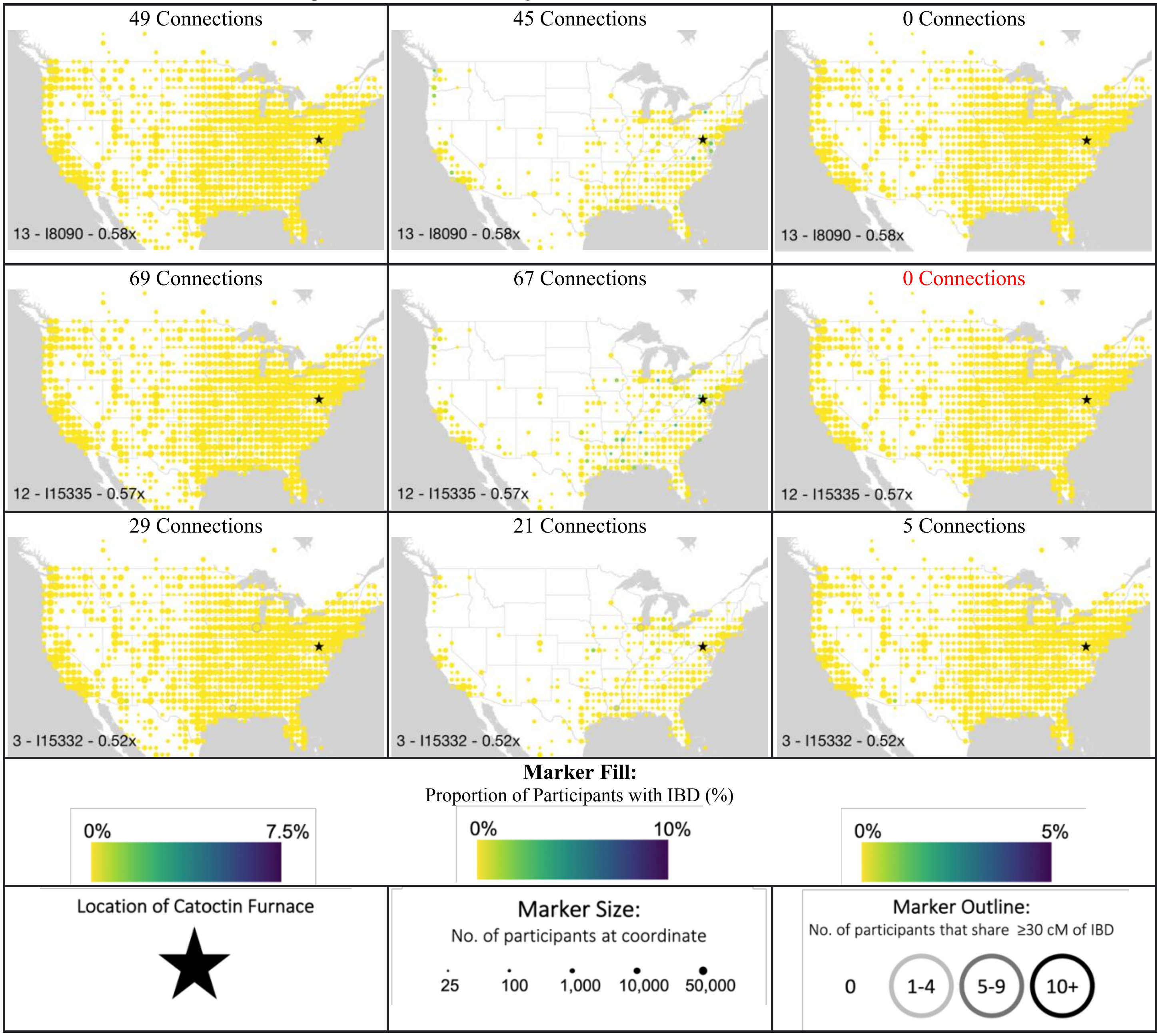

Fig. S12. Geographic distribution of distant and close relatives of each of the Catoctin individuals in the United States. The proportion of 23andMe participants at given geographic coordinates in the contiguous United States who share IBD with each of the 22 Catoctin individuals (one individual per row). Geographic coordinates are rounded to the nearest integer, and only coordinates that have at least 25 associated participants are shown. To further protect participant privacy, we randomly downsample to include results for only 80% of participants. The size of the marker corresponds to the number of participants associated with each site, while the color indicates the proportion of participants who share IBD with the Catoctin individuals. Marker outlines indicate the number of participants at each coordinate who share ≥30 cM of IBD with the specified Catoctin individual. Column A (left) reports results for all participants, column B (middle) shows participants with ≥5% Sub-Saharan African ancestry and column C (right) ≥99% European ancestry. The total number of IBD connections observed is reported above each image, when 0 connections are observed, this number is reported in red.

This is the author's version of the work. It is posted here by permission of the AAAS for personal use, not for redistribution. The definitive version was published in Science on August 4th, 2023, DOI: 10.1126/science.ade4995.

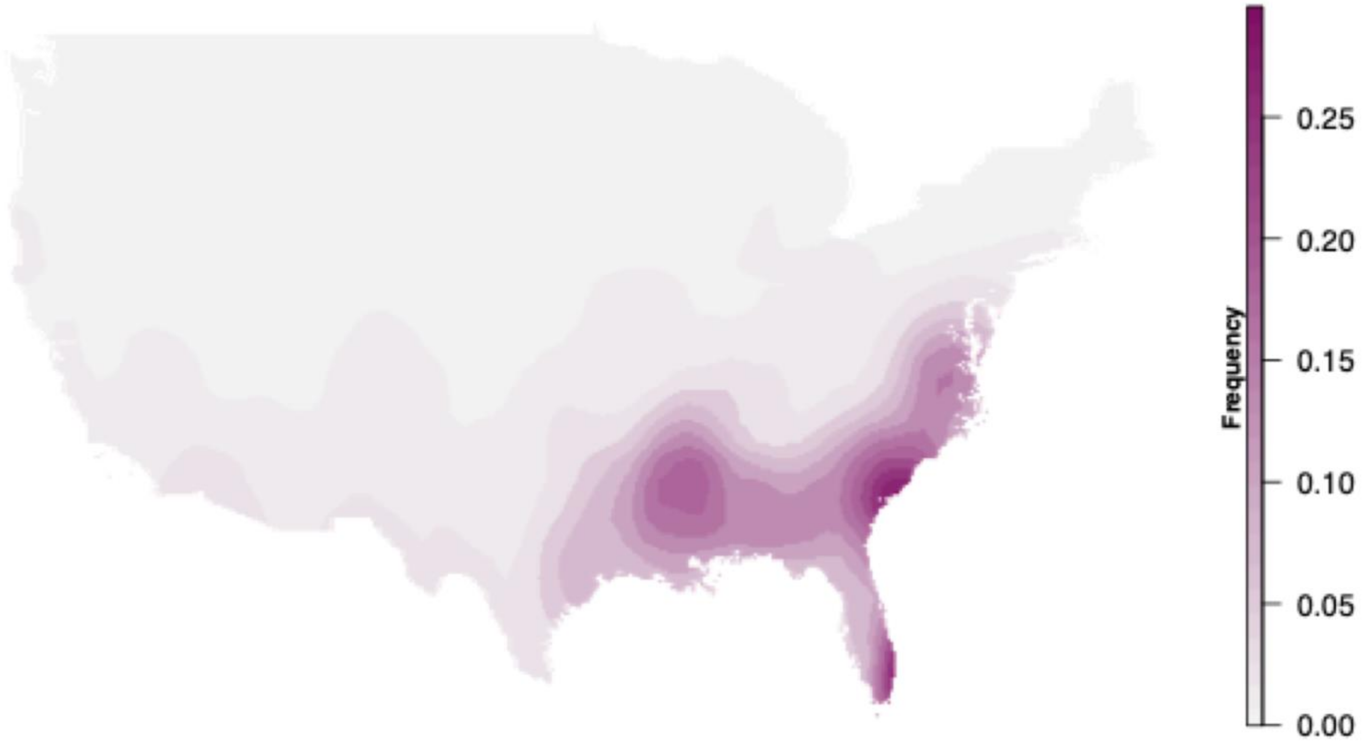

Fig. S13. Geographic distribution of 23andMe research participants with Sub-Saharan African ancestry in the contiguous United States. Predicted African ancestry proportions in the US using participants' grandparent birth locations. Prediction surfaces were generated using Kriging interpolation on the mean proportion of African ancestry at each unique geographic coordinate.

This is the author's version of the work. It is posted here by permission of the AAAS for personal use, not for redistribution. The definitive version was published in Science on August 4th, 2023, DOI: 10.1126/science.ade4995.

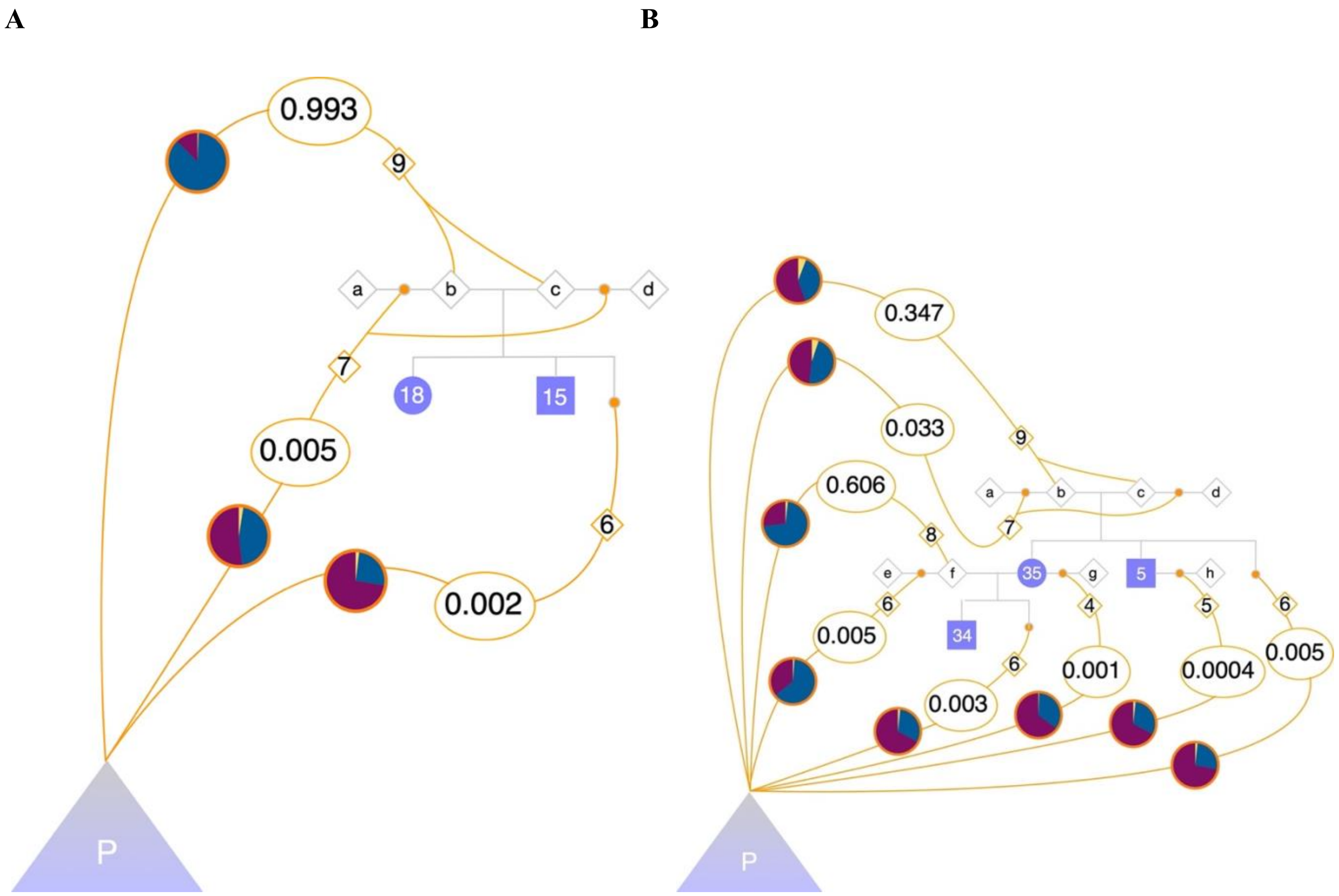

Fig. S14. Connections between Catoctin Family C and D and present-day pedigrees. The pedigree for Catoctin Family C (panel A) and Family D (panel B) is shown with blue-shaded individuals connected by grey lines. Open gray diamonds indicate un-genotyped individuals in each Catoctin family. The large purple triangle represents all present-day pedigrees composed of 23andMe research participants, and the probability distribution of how these pedigrees connect to the historical pedigree is inferred. Orange lines represent lineages connecting a present-day pedigree to the historical pedigree. Numbers in ovals give the probability that a present-day pedigree attached to a given point on the historical pedigree, scaled to a percentage. Numbers in diamonds indicate the average degree of a lineage connecting to a particular point. Pie charts show the average European (blue), African (red), and Indigenous American (yellow) admixture proportions of individuals in pedigrees whose most likely point of connection was through the lineage.

This is the author's version of the work. It is posted here by permission of the AAAS for personal use, not for redistribution. The definitive version was published in Science on August 4th, 2023, DOI: 10.1126/science.ade4995.

Sickle Cell Anemia/Trait (*rs334* or *i3003137*)

A T

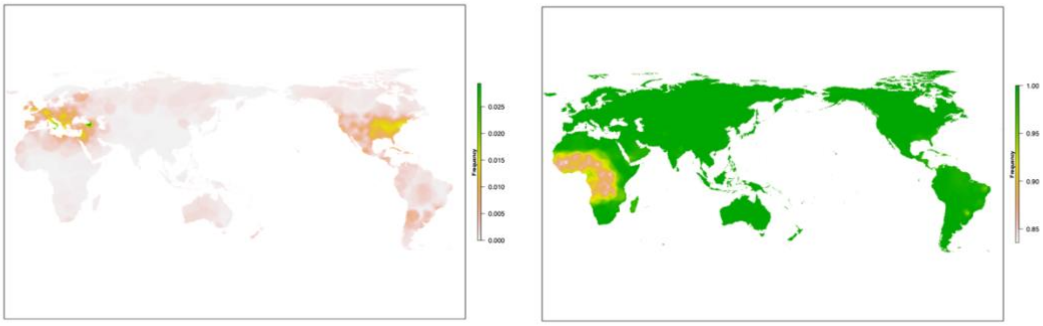

increased Risk of G6PD Deficiency (*rs1050828*)

T C

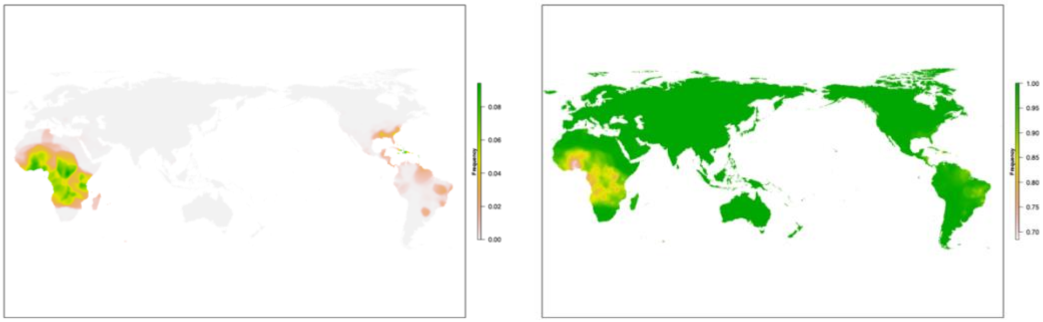

Fig. S15. Geographic distribution of biologically significant alleles. Heatmaps showing the geographic distribution of biologically significant alleles among 23andMe research participants using Kriging interpolation. For each phenotypically important SNP, the effect allele is shown in the left panel.

This is the author's version of the work. It is posted here by permission of the AAAS for personal use, not for redistribution. The definitive version was published in Science on August 4th, 2023, DOI: 10.1126/science.ade4995.

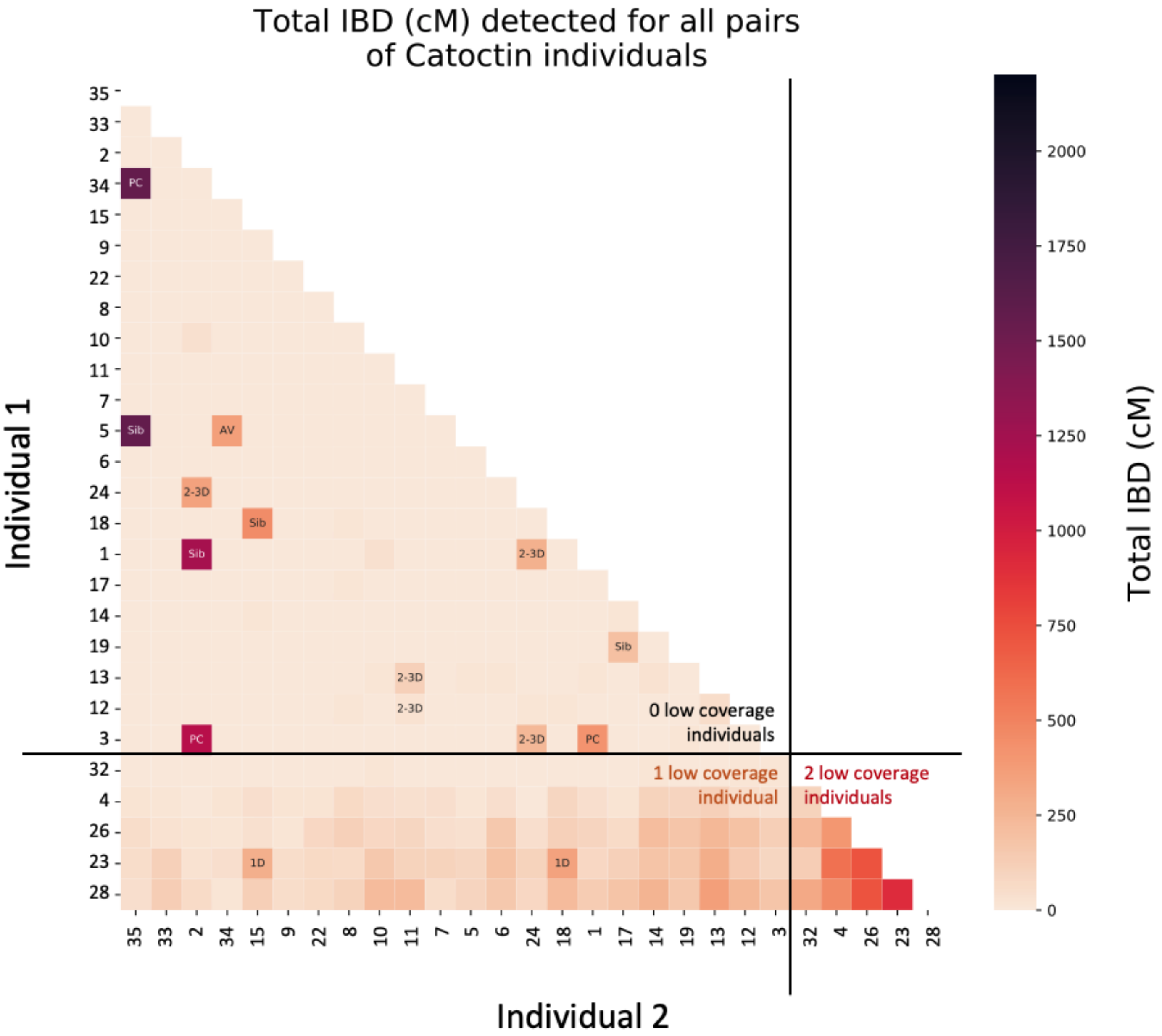

Fig. S16. Heatmap of total IBD measured between all pairs of Catoctin individuals. Individuals sorted from highest (top) to lowest (bottom) coverage. Comparisons between individuals with <0.5x coverage are likely to have high rates of false positive IBD, therefore black lines are used to separate individuals with >0.5x coverage from those with <0.5x coverage. Comparisons in the upper left quadrant contain the least false positive IBD as they involve no low coverage individuals, while comparisons in the lower right quadrant are expected to have very high rates of false positive IBD as they involve 2 low coverage individuals. Genetic relatives that were independently identified using an alternative method not based on imputation and IBD analysis (*80*) are annotated as follows: PC: Parent-Child; Sib: Sibling; AV: avuncular; 1D: 1st degree relative ; 2-3D: 2nd to 3rd degree relative. We do not observe high amounts of IBD among any pairs of individuals that were not already identified as genetic relatives using the allele frequency-based method, which can detect relatives as distant as the 3rd degree, suggesting that while close genetic relationships are common among the Catoctin individuals, more distant relationships are less common.

This is the author's version of the work. It is posted here by permission of the AAAS for personal use, not for redistribution. The definitive version was published in Science on August 4th, 2023, DOI: 10.1126/science.ade4995.

References 74 - 124

74. R. Pinhasi, D. M. Fernandes, K. Sirak, O. Cheronet, Isolating the human cochlea to generate bone powder for ancient DNA analysis. *Nature Protocols*. **14**, 1194–1205 (2019).

75. R. Pinhasi, D. Fernandes, K. Sirak, M. Novak, S. Connell, S. Alpaslan-Roodenberg, F. Gerritsen, V. Moiseyev, A. Gromov, P. Raczky, A. Anders, M. Pietrusewsky, G. Rollefson, M. Jovanovic, H. Trinhhoang, G. Bar-Oz, M. Oxenham, H. Matsumura, M. Hofreiter, Optimal Ancient DNA Yields from the Inner Ear Part of the Human Petrous Bone. *PLOS ONE*. **10**, e0129102 (2015).

76. I. Mathieson, S. Alpaslan-Roodenberg, C. Posth, A. Szécsényi-Nagy, N. Rohland, S. Mallick, I. Olalde, N. Broomandkhoshbacht, F. Candilio, O. Cheronet, D. Fernandes, M. Ferry, B. Gamarra, G. G. Fortes, W. Haak, E. Harney, E. Jones, D. Keating, B. Krause-Kyora, I. Kucukkalipci, M. Michel, A. Mittnik, K. Nägele, M. Novak, J. Oppenheimer, N. Patterson, S. Pfrengle, K. Sirak, K. Stewardson, S. Vai, S. Alexandrov, K. W. Alt, R. Andreescu, D. Antonović, A. Ash, N. Atanassova, K. Bacvarov, M. B. Gusztáv, H. Bocherens, M. Bolus, A. Boroneanţ, Y. Boyadzhiev, A. Budnik, J. Burmaz, S. Chohadzhiev, N. J. Conard, R. Cottiaux, M. Čuka, C. Cupillard, D. G. Drucker, N. Elenski, M. Francken, B. Galabova, G. Ganetsovski, B. Gély, T. Hajdu, V. Handzhyiska, K. Harvati, T. Higham, S. Iliev, I. Janković, I. Karavanić, D. J. Kennett, D. Komšo, A. Kozak, D. Labuda, M. Lari, C. Lazar, M. Leppek, K. Leshtakov, D. L. Vetro, D. Los, I. Lozanov, M. Malina, F. Martini, K. McSweeney, H. Meller, M. Menđušić, P. Mirea, V. Moiseyev, V. Petrova, T. D. Price, A. Simalcsik, L. Sineo, M. Šlaus, V. Slavchev, P. Stanev, A. Starović, T. Szeniczey, S. Talamo, M. Teschler-Nicola, C. Thevenet, I. Valchev, F. Valentin, S. Vasilyev, F. Veljanovska, S. Venelinova, E. Veselovskaya, B. Viola, C. Virag, J. Zaninović, S. Zäuner, P. W. Stockhammer, G. Catalano, R. Krauß, D. Caramelli, G. Zariņa, B. Gaydarska, M. Lillie, A. G. Nikitin, I. Potekhina, A. Papathanasiou, D. Borić, C. Bonsall, J. Krause, R. Pinhasi, D. Reich, The genomic history of southeastern Europe. *Nature*. **555**, 197–203 (2018).

77. D. M. Behar, M. Van Oven, S. Rosset, M. Metspalu, E.-L. Loogväli, N. M. Silva, T. Kivisild, A. Torroni, R. Villems, A "Copernican" reassessment of the human mitochondrial DNA tree from its root. *The American Journal of Human Genetics*. **90**, 675–684 (2012).

78. H. Li, R. Durbin, Fast and accurate long-read alignment with Burrows–Wheeler transform. *Bioinformatics*. **26**, 589–595 (2010).

79. P. Skoglund, B. H. Northoff, M. V. Shunkov, A. P. Derevianko, S. Pääbo, J. Krause, M. Jakobsson, Separating endogenous ancient DNA from modern day contamination in a Siberian Neandertal. *Proceedings of the National Academy of Sciences*. **111**, 2229–2234 (2014).

80. I. Olalde, S. Mallick, N. Patterson, N. Rohland, V. Villalba-Mouco, M. Silva, K. Dulias, C. J. Edwards, F. Gandini, M. Pala, P. Soares, M. Ferrando-Bernal, N. Adamski, N. Broomandkhoshbacht, O. Cheronet, B. J. Culleton, D. Fernandes, A. M. Lawson, M. Mah, J. Oppenheimer, K. Stewardson, Z. Zhang, J. M. J. Arenas, I. J. T. Moyano, D. C. Salazar-García, P. Castanyer, M. Santos, J. Tremoleda, M. Lozano, P. G. Borja, J. Fernández-Eraso, J. A. Mujika-Alustiza, C. Barroso, F. J. Bermúdez, E. V. Mínguez, J. Burch, N. Coromina, D. Vivó, A. Cebrià, J. M. Fullola, O. García-Puchol, J. I. Morales, F. X. Oms, T. Majó, J. M. Vergès, A. Díaz-Carvajal, I. Ollich-Castanyer, F. J. López-Cachero, A. M. Silva, C. Alonso-Fernández, G. D. de Castro, J. J. Echevarría, A. Moreno-Márquez, G. P. Berlanga, P. Ramos-García, J. Ramos-Muñoz, E. V. Vila, G. A. Arzo, Á. E. Arroyo, K. T. Lillios, J. Mack, J. Velasco-Vázquez, A. Waterman, L. B. de L. Enrich, M. B. Sánchez, B. Agustí, F. Codina, G. de Prado, A. Estalrrich, Á. F. Flores, C. Finlayson, G. Finlayson, S. Finlayson, F. Giles-Guzmán, A. Rosas, V. B. González, G. G. Atiénzar, M. S. H. Pérez, A. Llanos, Y. C. Marco, I. C. Beneyto, D. López-Serrano, M. S. Tormo, A. C. Valera, C. Blasco, C. Liesau, P. Ríos, J. Daura, M. J. de P. Michó, A. A. Diez-Castillo, R. F. Fernández, J. F. Farré, R. Garrido-Pena, V. S. Gonçalves, E. Guerra-Doce, A. M. Herrero-Corral, J. Juan-Cabanilles, D. López-Reyes, S. B. McClure, M. M. Pérez, A. O. Foix, M. S. Borràs, A. C. Sousa, J. M. V. Encinas, D. J. Kennett, M. B. Richards, K. W. Alt, W. Haak, R. Pinhasi, C. Lalueza-Fox, D. Reich, The genomic history of the Iberian Peninsula over the past 8000 years. *Science*. **363**, 1230–1234 (2019).

81. É. Harney, N. Patterson, D. Reich, J. Wakeley, Assessing the performance of qpAdm: a statistical tool for studying population admixture. *Genetics*. **217** (2021), doi:10.1093/genetics/iyaa045.

This is the author's version of the work. It is posted here by permission of the AAAS for personal use, not for redistribution. The definitive version was published in Science on August 4th, 2023, DOI: 10.1126/science.ade4995.

82. D. H. Alexander, J. Novembre, K. Lange, Fast model-based estimation of ancestry in unrelated individuals. *Genome Res.* **19**, 1655–1664 (2009).
83. N. Patterson, A. L. Price, D. Reich, Population Structure and Eigenanalysis. *PLOS Genetics*. **2**, e190 (2006).
84. V. M. Narasimhan, N. Patterson, P. Moorjani, N. Rohland, R. Bernardos, S. Mallick, I. Lazaridis, N. Nakatsuka, I. Olalde, M. Lipson, A. M. Kim, L. M. Olivieri, A. Coppa, M. Vidale, J. Mallory, V. Moiseyev, E. Kitov, J. Monge, N. Adamski, N. Alex, N. Broomandkhoshbacht, F. Candilio, K. Callan, O. Cheronet, B. J. Culleton, M. Ferry, D. Fernandes, S. Freilich, B. Gamarra, D. Gaudio, M. Hajdinjak, É. Harney, T. K. Harper, D. Keating, A. M. Lawson, M. Mah, K. Mandl, M. Michel, M. Novak, J. Oppenheimer, N. Rai, K. Sirak, V. Slon, K. Stewardson, F. Zalzala, Z. Zhang, G. Akhatov, A. N. Bagashev, A. Bagnera, B. Baitanayev, J. Bendezu-Sarmiento, A. A. Bissembaev, G. L. Bonora, T. T. Chargynov, T. Chikisheva, P. K. Dashkovskiy, A. Derevianko, M. Dobeš, K. Douka, N. Dubova, M. N. Duisengali, D. Enshin, A. Epimakhov, A. V. Fribus, D. Fuller, A. Goryachev, A. Gromov, S. P. Grushin, B. Hanks, M. Judd, E. Kazizov, A. Khokhlov, A. P. Krygin, E. Kupriyanova, P. Kuznetsov, D. Luiselli, F. Maksudov, A. M. Mamedov, T. B. Mamirov, C. Meiklejohn, D. C. Merrett, R. Micheli, O. Mochalov, S. Mustafokulov, A. Nayak, D. Pettener, R. Potts, D. Razhev, M. Rykun, S. Sarno, T. M. Savenkova, K. Sikhymbaeva, S. M. Slepchenko, O. A. Soltobaev, N. Stepanova, S. Svyatko, K. Tabaldiev, M. Teschler-Nicola, A. A. Tishkin, V. V. Tkachev, S. Vasilyev, P. Velemínský, D. Voyakin, A. Yermolayeva, M. Zahir, V. S. Zubkov, A. Zubova, V. S. Shinde, C. Lalueza-Fox, M. Meyer, D. Anthony, N. Boivin, K. Thangaraj, D. J. Kennett, M. Frachetti, R. Pinhasi, D. Reich, The formation of human populations in South and Central Asia. *Science*. **365** (2019), doi:10.1126/science.aat7487.
85. L. Van Dorp, S. Lowes, J. L. Weigel, N. Ansari-Pour, S. López, J. Mendoza-Revilla, J. A. Robinson, J. Henrich, M. G. Thomas, N. Nunn, Genetic legacy of state centralization in the Kuba Kingdom of the Democratic Republic of the Congo. *Proceedings of the National Academy of Sciences*. **116**, 593–598 (2019).
86. B. A. Jackson, J. L. Wilson, S. Kirbah, S. S. Sidney, J. Rosenberger, L. Bassie, J. A. Alie, D. C. McLean, W. T. Garvey, B. Ely, Mitochondrial DNA genetic diversity among four ethnic groups in Sierra Leone. *American journal of physical anthropology*. **128**, 156–163 (2005).
87. B. M. Henn, C. R. Gignoux, M. Jobin, J. M. Granka, J. Macpherson, J. M. Kidd, L. Rodríguez-Botigué, S. Ramachandran, L. Hon, A. Brisbin, Hunter-gatherer genomic diversity suggests a southern African origin for modern humans. *Proceedings of the National Academy of Sciences*. **108**, 5154–5162 (2011).
88. V. D. Blondel, J.-L. Guillaume, R. Lambiotte, E. Lefebvre, Fast unfolding of communities in large networks. *Journal of statistical mechanics: theory and experiment*. **2008**, P10008 (2008).
89. M. Bastian, S. Heymann, M. Jacomy, "Gephi: an open source software for exploring and manipulating networks" in (2009).
90. Enslaved faces from African American cemetery in Catoctin, Maryland recreated - The Washington Post, (available at https://www.washingtonpost.com/history/2021/07/09/african-american-cemetery-catoctin-enslaved-faces/).
91. A. Roberts, Historical Society unveils facial reconstructions of African-Americans once enslaved in Thurmont. *The Frederick News-Post*, (available at https://www.fredericknewspost.com/news/economy_and_business/tourism/historical-society-unveils-facial-reconstructions-of-african-americans-once-enslaved-in-thurmont/article_57a8171b-f3e2-52c8-bb8d-a6fc6d189ef2.html).
92. S. M. Fullerton, S. S.-J. Lee, Secondary uses and the governance of de-identified data: Lessons from the human genome diversity panel. *BMC Medical Ethics*. **12**, 16 (2011).
93. D. B. Resnik, The Human Genome Diversity Project: Ethical Problems and Solutions. *Politics and the Life Sciences*. **18**, 15–23 (1999).
94. J. O'Connell, T. Yun, M. Moreno, H. Li, N. Litterman, A. Kolesnikov, E. Noblin, P.-C. Chang, A. Shastri, E. H. Dorfman, S. Shringarpure, A. Auton, A. Carroll, C. Y. McLean, A population-specific reference panel for improved genotype imputation in African Americans. *Commun Biol.* **4**, 1–9 (2021).
95. E. R. Ebel, F. Reis, D. A. Petrov, S. Beleza, Historical trends and new surveillance of Plasmodium falciparum drug resistance markers in Angola. *Malaria Journal*. **20**, 175 (2021).
96. L. Sweeney, k-ANONYMITY: A MODEL FOR PROTECTING PRIVACY. *Int. J. Unc. Fuzz. Knowl. Based Syst.* **10**, 557–570 (2002).
97. E. Birney, M. Inouye, J. Raff, A. Rutherford, A. Scally, The language of race, ethnicity, and ancestry in human

This is the author's version of the work. It is posted here by permission of the AAAS for personal use, not for redistribution. The definitive version was published in Science on August 4th, 2023, DOI: 10.1126/science.ade4995.
genetic research (2021), , doi:10.48550/arXiv.2106.10041.
98. J. K. Wagner, J.-H. Yu, J. O. Ifekwunigwe, T. M. Harrell, M. J. Bamshad, C. D. Royal, Anthropologists' views on race, ancestry, and genetics. *American Journal of Physical Anthropology*. **162**, 318–327 (2017).
99. I. Mathieson, A. Scally, What is ancestry? *PLOS Genetics*. **16**, e1008624 (2020).
100. C. Agyemang, R. Bhopal, M. Bruijnzeels, Negro, Black, Black African, African Caribbean, African American or what? Labelling African origin populations in the health arena in the 21st century. *Journal of Epidemiology & Community Health*. **59**, 1014–1018 (2005).
101. AP says it will capitalize Black but not white | AP News, (available at https://apnews.com/article/entertainment-cultures-race-and-ethnicity-us-news-ap-top-news-7e36c00c5af0436abc09e051261fff1f).
102. G. Coop, Genetic similarity versus genetic ancestry groups as sample descriptors in human genetics (2023), , doi:10.48550/arXiv.2207.11595.
103. A. C. F. Lewis, S. J. Molina, P. S. Appelbaum, B. Dauda, A. Di Rienzo, A. Fuentes, S. M. Fullerton, N. A. Garrison, N. Ghosh, E. M. Hammonds, D. S. Jones, E. E. Kenny, P. Kraft, S. S.-J. Lee, M. Mauro, J. Novembre, A. Panofsky, M. Sohail, B. M. Neale, D. S. Allen, Getting genetic ancestry right for science and society. *Science*. **376**, 250–252 (2022).
104. M. Miyagi, E. M. Guthman, S. D.-K. Sun, Transgender rights rely on inclusive language. *Science*. **374**, 1568–1569 (2021).
105. J. Brück, Ancient DNA, kinship and relational identities in Bronze Age Britain. *Antiquity*. **95**, 228–237 (2021).
106. S. S. Ebenesersdóttir, M. Sandoval-Velasco, E. D. Gunnarsdóttir, A. Jagadeesan, V. B. Guðmundsdóttir, E. L. Thordardóttir, M. S. Einarsdóttir, K. H. S. Moore, Á. Sigurðsson, D. N. Magnúsdóttir, H. Jónsson, S. Snorradóttir, E. Hovig, P. Møller, I. Kockum, T. Olsson, L. Alfredsson, T. F. Hansen, T. Werge, G. L. Cavalleri, E. Gilbert, C. Lalueza-Fox, J. W. Walser, S. Kristjánsdóttir, S. Gopalakrishnan, L. Árnadóttir, Ó. Þ. Magnússon, M. T. P. Gilbert, K. Stefánsson, A. Helgason, Ancient genomes from Iceland reveal the making of a human population. *Science*. **360**, 1028–1032 (2018).
107. I. Olalde, S. Brace, M. E. Allentoft, I. Armit, K. Kristiansen, T. Booth, N. Rohland, S. Mallick, A. Szécsényi-Nagy, A. Mittnik, E. Altena, M. Lipson, I. Lazaridis, T. K. Harper, N. Patterson, N. Broomandkhoshbacht, Y. Diekmann, Z. Faltyskova, D. Fernandes, M. Ferry, E. Harney, P. de Knijff, M. Michel, J. Oppenheimer, K. Stewardson, A. Barclay, K. W. Alt, C. Liesau, P. Ríos, C. Blasco, J. V. Miguel, R. M. García, A. A. Fernández, E. Bánffy, M. Bernabò-Brea, D. Billoin, C. Bonsall, L. Bonsall, T. Allen, L. Büster, S. Carver, L. C. Navarro, O. E. Craig, G. T. Cook, B. Cunliffe, A. Denaire, K. E. Dinwiddy, N. Dodwell, M. Ernée, C. Evans, M. Kuchařík, J. F. Farré, C. Fowler, M. Gazenbeek, R. G. Pena, M. Haber-Uriarte, E. Haduch, G. Hey, N. Jowett, T. Knowles, K. Massy, S. Pfrengle, P. Lefranc, O. Lemercier, A. Lefebvre, C. H. Martínez, V. G. Olmo, A. B. Ramírez, J. L. Maurandi, T. Majó, J. I. McKinley, K. McSweeney, B. G. Mende, A. Modi, G. Kulcsár, V. Kiss, A. Czene, R. Patay, A. Endrődi, K. Köhler, T. Hajdu, T. Szeniczey, J. Dani, Z. Bernert, M. Hoole, O. Cheronet, D. Keating, P. Velemínský, M. Dobeš, F. Candilio, F. Brown, R. F. Fernández, A.-M. Herrero-Corral, S. Tusa, E. Carnieri, L. Lentini, A. Valenti, A. Zanini, C. Waddington, G. Delibes, E. Guerra-Doce, B. Neil, M. Brittain, M. Luke, R. Mortimer, J. Desideri, M. Besse, G. Brücken, M. Furmanek, A. Hałuszko, M. Mackiewicz, A. Rapiński, S. Leach, I. Soriano, K. T. Lillios, J. L. Cardoso, M. P. Pearson, P. Włodarczak, T. D. Price, P. Prieto, P.-J. Rey, R. Risch, M. A. Rojo Guerra, A. Schmitt, J. Serralongue, A. M. Silva, V. Smrčka, L. Vergnaud, J. Zilhão, D. Caramelli, T. Higham, M. G. Thomas, D. J. Kennett, H. Fokkens, V. Heyd, A. Sheridan, K.-G. Sjögren, P. W. Stockhammer, J. Krause, R. Pinhasi, W. Haak, I. Barnes, C. Lalueza-Fox, D. Reich, The Beaker phenomenon and the genomic transformation of northwest Europe. *Nature*. **555**, 190–196 (2018).
108. "Allen Ancient Genome Diversity Project / John Templeton Ancient DNA Atlas."
109. I. Lazaridis, D. Nadel, G. Rollefson, D. C. Merrett, N. Rohland, S. Mallick, D. Fernandes, M. Novak, B. Gamarra, K. Sirak, S. Connell, K. Stewardson, E. Harney, Q. Fu, G. Gonzalez-Fortes, E. R. Jones, S. A. Roodenberg, G. Lengyel, F. Bocquentin, B. Gasparian, J. M. Monge, M. Gregg, V. Eshed, A.-S. Mizrahi, C. Meiklejohn, F. Gerritsen, L. Bejenaru, M. Blüher, A. Campbell, G. Cavalleri, D. Comas, P. Froguel, E. Gilbert, S. M. Kerr, P. Kovacs, J. Krause, D. McGettigan, M. Merrigan, D. A. Merriwether, S. O'Reilly, M. B. Richards, O. Semino, M. Shamoon-Pour, G. Stefanescu, M. Stumvoll, A. Tönjes, A. Torroni, J. F. Wilson, L. Yengo, N. A. Hovhannisyan, N. Patterson, R. Pinhasi, D. Reich, Genomic insights into the origin of farming in the ancient Near East. *Nature*. **536**, 419–424 (2016).

This is the author's version of the work. It is posted here by permission of the AAAS for personal use, not for redistribution. The definitive version was published in Science on August 4th, 2023, DOI: 10.1126/science.ade4995.

110. M. G. Llorente, E. R. Jones, A. Eriksson, V. Siska, K. W. Arthur, J. W. Arthur, M. C. Curtis, J. T. Stock, M. Coltorti, P. Pieruccini, S. Stretton, F. Brock, T. Higham, Y. Park, M. Hofreiter, D. G. Bradley, J. Bhak, R. Pinhasi, A. Manica, Ancient Ethiopian genome reveals extensive Eurasian admixture in Eastern Africa. *Science* (2015), doi:10.1126/science.aad2879.

111. P. de B. Damgaard, R. Martiniano, J. Kamm, J. V. Moreno-Mayar, G. Kroonen, M. Peyrot, G. Barjamovic, S. Rasmussen, C. Zacho, N. Baimukhanov, V. Zaibert, V. Merz, A. Biddanda, I. Merz, V. Loman, V. Evdokimov, E. Usmanova, B. Hemphill, A. Seguin-Orlando, F. E. Yediay, I. Ullah, K.-G. Sjögren, K. H. Iversen, J. Choin, C. de la Fuente, M. Ilardo, H. Schroeder, V. Moiseyev, A. Gromov, A. Polyakov, S. Omura, S. Y. Senyurt, H. Ahmad, C. McKenzie, A. Margaryan, A. Hameed, A. Samad, N. Gul, M. H. Khokhar, O. I. Goriunova, V. I. Bazaliiskii, J. Novembre, A. W. Weber, L. Orlando, M. E. Allentoft, R. Nielsen, K. Kristiansen, M. Sikora, A. K. Outram, R. Durbin, E. Willerslev, The first horse herders and the impact of early Bronze Age steppe expansions into Asia. *Science* (2018), doi:10.1126/science.aar7711.

112. C. Gamba, E. R. Jones, M. D. Teasdale, R. L. McLaughlin, G. Gonzalez-Fortes, V. Mattiangeli, L. Domboróczki, I. Kővári, I. Pap, A. Anders, A. Whittle, J. Dani, P. Raczky, T. F. G. Higham, M. Hofreiter, D. G. Bradley, R. Pinhasi, Genome flux and stasis in a five millennium transect of European prehistory. *Nat Commun*. **5**, 5257 (2014).

113. D. Gokhman, E. Lavi, K. Prüfer, M. F. Fraga, J. A. Riancho, J. Kelso, S. Pääbo, E. Meshorer, L. Carmel, Reconstructing the DNA Methylation Maps of the Neandertal and the Denisovan. *Science*. **344**, 523–527 (2014).

114. T. Günther, H. Malmström, E. M. Svensson, A. Omrak, F. Sánchez-Quinto, G. M. Kılınç, M. Krzewińska, G. Eriksson, M. Fraser, H. Edlund, A. R. Munters, A. Coutinho, L. G. Simões, M. Vicente, A. Sjölander, B. J. Sellevold, R. Jørgensen, P. Claes, M. D. Shriver, C. Valdiosera, M. G. Netea, J. Apel, K. Lidén, B. Skar, J. Storå, A. Götherström, M. Jakobsson, Population genomics of Mesolithic Scandinavia: Investigating early postglacial migration routes and high-latitude adaptation. *PLOS Biology*. **16**, e2003703 (2018).

115. M. Ferrando-Bernal, C. Morcillo-Suarez, T. de-Dios, P. Gelabert, S. Civit, A. Díaz-Carvajal, I. Ollich-Castanyer, M. E. Allentoft, S. Valverde, C. Lalueza-Fox, Mapping co-ancestry connections between the genome of a Medieval individual and modern Europeans. *Scientific Reports*. **10**, 6843 (2020).

116. A. Margaryan, D. J. Lawson, M. Sikora, F. Racimo, S. Rasmussen, I. Moltke, L. M. Cassidy, E. Jørsboe, A. Ingason, M. W. Pedersen, T. Korneliussen, H. Wilhelmson, M. M. Buś, P. de Barros Damgaard, R. Martiniano, G. Renaud, C. Bhérer, J. V. Moreno-Mayar, A. K. Fotakis, M. Allen, R. Allmäe, M. Molak, E. Cappellini, G. Scorrano, H. McColl, A. Buzhilova, A. Fox, A. Albrechtsen, B. Schütz, B. Skar, C. Arcini, C. Falys, C. H. Jonson, D. Błaszczyk, D. Pezhemsky, G. Turner-Walker, H. Gestsdóttir, I. Lundstrøm, I. Gustin, I. Mainland, I. Potekhina, I. M. Muntoni, J. Cheng, J. Stenderup, J. Ma, J. Gibson, J. Peets, J. Gustafsson, K. H. Iversen, L. Simpson, L. Strand, L. Loe, M. Sikora, M. Florek, M. Vretemark, M. Redknap, M. Bajka, T. Pushkina, M. Søvsø, N. Grigoreva, T. Christensen, O. Kastholm, O. Uldum, P. Favia, P. Holck, S. Sten, S. V. Arge, S. Ellingvåg, V. Moiseyev, W. Bogdanowicz, Y. Magnusson, L. Orlando, P. Pentz, M. D. Jessen, A. Pedersen, M. Collard, D. G. Bradley, M. L. Jørkov, J. Arneborg, N. Lynnerup, N. Price, M. T. P. Gilbert, M. E. Allentoft, J. Bill, S. M. Sindbæk, L. Hedeager, K. Kristiansen, R. Nielsen, T. Werge, E. Willerslev, Population genomics of the Viking world. *Nature*. **585**, 390–396 (2020).

117. M. Sikora, V. V. Pitulko, V. C. Sousa, M. E. Allentoft, L. Vinner, S. Rasmussen, A. Margaryan, P. de Barros Damgaard, C. de la Fuente, G. Renaud, M. A. Yang, Q. Fu, I. Dupanloup, K. Giampoudakis, D. Nogués-Bravo, C. Rahbek, G. Kroonen, M. Peyrot, H. McColl, S. V. Vasilyev, E. Veselovskaya, M. Gerasimova, E. Y. Pavlova, V. G. Chasnyk, P. A. Nikolskiy, A. V. Gromov, V. I. Khartanovich, V. Moiseyev, P. S. Grebenyuk, A. Y. Fedorchenko, A. I. Lebedintsev, S. B. Slobodin, B. A. Malyarchuk, R. Martiniano, M. Meldgaard, L. Arppe, J. U. Palo, T. Sundell, K. Mannermaa, M. Putkonen, V. Alexandersen, C. Primeau, N. Baimukhanov, R. S. Malhi, K.-G. Sjögren, K. Kristiansen, A. Wessman, A. Sajantila, M. M. Lahr, R. Durbin, R. Nielsen, D. J. Meltzer, L. Excoffier, E. Willerslev, The population history of northeastern Siberia since the Pleistocene. *Nature*. **570**, 182–188 (2019).

118. T. Kivisild, L. Saag, R. Hui, S. A. Biagini, V. Pankratov, E. D'Atanasio, L. Pagani, L. Saag, S. Rootsi, R. Mägi, E. Metspalu, H. Valk, M. Malve, K. Irdt, T. Reisberg, A. Solnik, C. L. Scheib, D. N. Seidman, A. L. Williams, K. Tambets, M. Metspalu, Patterns of genetic connectedness between modern and medieval Estonian genomes reveal the origins of a major ancestry component of the Finnish population. *The American Journal of Human Genetics*. **108**, 1792–1806 (2021).

119. C. D. Huff, D. J. Witherspoon, T. S. Simonson, J. Xing, W. S. Watkins, Y. Zhang, T. M. Tuohy, D. W. Neklason, R.

This is the author's version of the work. It is posted here by permission of the AAAS for personal use, not for redistribution. The definitive version was published in Science on August 4th, 2023, DOI: 10.1126/science.ade4995.
W. Burt, S. L. Guthery, S. R. Woodward, L. B. Jorde, Maximum-likelihood estimation of recent shared ancestry (ERSA). *Genome Res.* **21**, 768–774 (2011).

120. A. THOMAS, M. H. SKOLNICK, C. M. LEWIS, Genomic mismatch scanning in pedigrees. *Mathematical Medicine and Biology: A Journal of the IMA*. **11**, 1–16 (1994).

121. A. W. Wohns, Y. Wong, B. Jeffery, A. Akbari, S. Mallick, R. Pinhasi, N. Patterson, D. Reich, J. Kelleher, G. McVean, A unified genealogy of modern and ancient genomes (2021), p. 2021.02.16.431497, , doi:10.1101/2021.02.16.431497.

122. M. H. Gouveia, V. Borda, T. P. Leal, R. G. Moreira, A. W. Bergen, F. S. G. Kehdy, I. Alvim, M. M. Aquino, G. S. Araujo, N. M. Araujo, V. Furlan, R. Liboredo, M. Machado, W. C. S. Magalhaes, L. A. Michelin, M. R. Rodrigues, F. Rodrigues-Soares, H. P. Sant Anna, M. L. Santolalla, M. O. Scliar, G. Soares-Souza, R. Zamudio, C. Zolini, M. C. Bortolini, M. Dean, R. H. Gilman, H. Guio, J. Rocha, A. C. Pereira, M. L. Barreto, B. L. Horta, M. F. Lima-Costa, S. M. Mbulaiteye, S. J. Chanock, S. A. Tishkoff, M. Yeager, E. Tarazona-Santos, Origins, Admixture Dynamics, and Homogenization of the African Gene Pool in the Americas. *Molecular Biology and Evolution*. **37**, 1647–1656 (2020).

123. F. L. C. Jackson, So many Nigerians: why is Nigeria overrepresented as the ancestral genetic homeland of Legacy African North Americans? *The American Journal of Human Genetics*. **108**, 202–208 (2021).

124. A. Ko, R. Nielsen, Composite likelihood method for inferring local pedigrees. *PLOS Genetics*. **13**, e1006963 (2017).
